# Routine Vaccination Coverage in Northern Nigeria: Results from 40 District-Level Cluster Surveys, 2014-2015

**DOI:** 10.1371/journal.pone.0167835

**Published:** 2016-12-09

**Authors:** Rajni Gunnala, Ikechukwu U. Ogbuanu, Oluwasegun J. Adegoke, Heather M. Scobie, Belinda V. Uba, Kathleen A. Wannemuehler, Alicia Ruiz, Hashim Elmousaad, Chima J. Ohuabunwo, Mahmud Mustafa, Patrick Nguku, Ndadilnasiya Endie Waziri, John F. Vertefeuille

**Affiliations:** 1 U.S. Centers for Disease Control and Prevention, Global Immunization Division, Atlanta, Georgia, United States of America; 2 Nigeria National Stop Transmission of Polio, Abuja, Nigeria; 3 National Primary Health Care Development Agency, Abuja, Nigeria; 4 Nigeria Field Epidemiology and Laboratory Training Program, Abuja, Nigeria; Public Health England, UNITED KINGDOM

## Abstract

**Background:**

Despite recent success towards controlling poliovirus transmission, Nigeria has struggled to achieve uniformly high routine vaccination coverage. A lack of reliable vaccination coverage data at the operational level makes it challenging to target program improvement. To reliably estimate vaccination coverage, we conducted district-level vaccine coverage surveys using a pre-existing infrastructure of polio technical staff in northern Nigeria.

**Methods:**

Household-level cluster surveys were conducted in 40 polio high risk districts of Nigeria during 2014–2015. Global positioning system technology and intensive supervision by a pool of qualified technical staff were used to ensure high survey quality. Vaccination status of children aged 12–23 months was documented based on vaccination card or caretaker’s recall. District-level coverage estimates were calculated using survey methods.

**Results:**

Data from 7,815 children across 40 districts were analyzed. District-level coverage with the third dose of diphtheria-pertussis-tetanus vaccine (DPT3) ranged widely from 1–63%, with all districts having DPT3 coverage below the target of 80%. Median coverage across all districts for each of eight vaccine doses (1 Bacille Calmette-Guérin dose, 3 DPT doses, 3 oral poliovirus vaccine doses, and 1 measles vaccine dose) was <50%. DPT3 coverage by survey was substantially lower (range: 28%–139%) than the 2013 administrative coverage reported among children aged <12 months. Common reported reasons for non-vaccination included lack of knowledge about vaccines and vaccination services (50%) and factors related to access to routine immunization services (15%).

**Conclusions:**

Survey results highlighted vaccine coverage gaps that were systematically underestimated by administrative reporting across 40 polio high risk districts in northern Nigeria. Given the limitations of administrative coverage data, our approach to conducting quality district-level coverage surveys and providing data to assess and remediate issues contributing to poor vaccination coverage could serve as an example in countries with sub-optimal vaccination coverage, similar to Nigeria.

## Introduction

Vaccine preventable diseases cause significant mortality among children aged under 5 years [1]. In 2012, the World Health Assembly endorsed the Global Vaccine Action Plan, which includes an aim of ensuring delivery of universal access to immunization with associated targets of reaching 90% national vaccination coverage and at least 80% vaccination coverage in every district [2]. To support this goal and to respond to the 2012 World Health Assembly’s declaration of polio eradication as a public health emergency, Nigeria is working to achieve polio-free status [3], and continues to strengthen routine immunization (RI) service delivery to achieve high and equitable coverage [4].

In Nigeria, the Expanded Program on Immunization (EPI) was launched in 1979 and provides routine vaccinations to all children aged <12 months and pregnant women [5]. Core vaccines provided to infants as part of EPI include Bacille Calmette-Guérin vaccine (BCG), diphtheria-pertussis-tetanus vaccine (DPT), oral poliovirus vaccine (OPV), and measles vaccine. Though centrally managed by the National Primary Health Care Development Agency (NPHCDA) of the Federal Ministry of Health (FMOH), RI services are organized and implemented at subnational levels, including states and Local Government Areas (LGAs), which are equivalent to districts. Given the variation in sociodemographic characteristics of the population over a large number of states (36 plus the Federal Capital Territory, or FCT) and LGAs (774), RI service delivery must be tailored to suit local needs and assure high, uniform vaccination coverage.

During 2010–2014, national coverage with the third dose of DPT vaccine (DPT3), a key indicator of RI program performance, i.e., the percentage of children aged <12 months reported to have received the vaccine dose, ranged from 57%–74% by administrative reporting [6]. Multiple coverage surveys suggest even lower vaccination coverage than reported by administrative data and demonstrate that national estimates can often mask subnational immunity gaps at state and LGA levels [7]. Data from the 2013 National Demographic and Health Survey (DHS) show that while the national estimate for DPT3 coverage was 38%, state-level coverage ranged from 3%–76% across 20 states in the northern part of the country [8].

A lack of reliable vaccination coverage data at the operational-level (i.e., LGA), makes it challenging to monitor and remediate RI service delivery to achieve uniformly high vaccination coverage. To fill this information gap, we used a pre-existing infrastructure of polio technical field staff to conduct RI coverage surveys in 40 polio high-risk LGAs across eight states in northern Nigeria during 2014–2015. The overall survey objectives of providing LGA-level vaccine coverage estimates and RI service delivery information to LGA immunization staff for use in planning and program improvement were successfully achieved.

## Materials and Methods

### Survey Setting

The National Stop Transmission of Polio (NSTOP) program was collaboratively established in 2012 by the NPHCDA, the Nigeria Field Epidemiology and Laboratory Training Program (NFELTP) and U.S. Centers for Disease Control and Prevention (CDC), to place staff at national, state and LGA levels to strengthen RI service delivery with the goal of aiding polio eradication efforts. Senior NSTOP staff placed at the central and state level are graduates of the 2-year NFELTP program, including classroom training on polio and RI services and supervisory experience during OPV supplemental immunization activities (SIAs) [9]. At the LGA level, 100 NSTOP staff were hired in 100 LGAs deemed to be at high risk for polio based on the CDC-Global Good risk analysis algorithm [10]. The LGA-level coverage surveys provided an opportunity to strengthen capacity of staff and obtain data about RI service delivery performance. As neither NSTOP nor NFELTP staff are involved with service delivery at the health facility or community levels, they provided a pool of independent, qualified field staff and a supervisory structure that was well-suited for implementing high quality coverage surveys within a short timeframe.

### Survey Population

The target population was children aged 12–23 months living in 100 polio high-risk LGAs of northern Nigeria where NSTOP staff had been assigned. Forty high-risk LGAs were selected for the survey using the following algorithm. We excluded the insecure states of Borno and Yobe, and also Kano state, where ongoing LGA-level coverage surveys were being conducted (Dale Rhoda, KANRICS Task Force, and Biostat Global Consulting, personal communication). In the remaining states, all LGAs were included except in states with more than six total polio high-risk LGAs, where three LGAs with the highest administrative DPT3 coverage from 2013 and three LGAs with the lowest DPT3 coverage were chosen. The exception was Kaduna state, where two additional LGAs were included for the survey pilot phase. Thus, included LGAs were from Bauchi (which had four polio high risk LGAs out of the 20 total LGAs in the state), Jigawa (six out of 27), Kaduna (eight out of 23), Katsina (six out of 34), Kebbi (three out of 21), Sokoto (six out of 23), and Zamfara (six out of 14) states, and the FCT (one).

### Survey Design

We conducted a two-stage household-level cluster survey in each LGA using the 2005 World Health Organization (WHO) survey methodology [11]. The sampling frame of primary sampling units (clusters) was a list of enumeration areas (EA) obtained from the National Population Commission (NPC). In the first stage, 30 clusters in each LGA were systematically sampled probability proportional to estimated size, using 2006 census data. PDF maps for selected clusters were obtained from NPC. In the second stage, a standardized protocol was used to select households, defined as groups of persons living and eating together under the same roof. To reduce potential selection bias in the field, pre-assignment of random start points for each cluster was done at the central level. From the start point, teams followed a standardized path through the cluster (moving clockwise and turning right at every opportunity). All housing structures along the path were visited to determine if they contained eligible children 12–23 months of age. Only one eligible child per household was included in the survey; if multiple eligible children lived in the household, one child was selected using a random number table. Teams continued visiting households until they enrolled a total of seven households with eligible children within each cluster. Households that could not be contacted or who declined to participate were replaced. The expected sample size for each LGA survey was 210 children (8,400 in 40 LGAs), based on an expected coverage of 50%, desired precision of +/- 10% with 95% confidence intervals, and a design effect of 2.0 [11]. This survey was approved by the National Health Research Ethics Committee of Nigeria and was determined to be a non-research, public health program evaluation by the Human Research Protection Office of the U.S. CDC, according to its human subjects’ procedures.

### Survey Implementation

Data collection tools were tested in March 2014. Implementation of data collection followed in three phases: a pilot phase was conducted in two LGAs in Kaduna (May 2014), followed by Phase 1 (October 2014) and Phase 2 (January 2015) in 19 LGAs each that were delayed due to human resource limitations secondary to ongoing Ebola outbreak response in Nigeria [12]. A central command center for the survey was established in Abuja which included a NSTOP central coordinator, NSTOP data managers, and a GPS specialist. Senior supervisors were central and state-level NSTOP staff that supervised multiple LGAs. Three survey teams were allocated per LGA, with each team consisting of one team supervisor (mostly NFELTP residents and several staff from NPC, FMOH, and NPHCDA) and two interviewers (who were recruited locally and required to have a university degree, be fluent in English and Hausa, and preferably have experience with SIAs). An excess number of interviewers was recruited within the respective LGA and trained on the survey, with final selection for field work dependent on their performance during the training and post-training evaluation.

During the pilot phase, senior and team supervisors were trained centrally by CDC staff and central NSTOP staff over 3 days in Abuja, followed by training of interviewers over 2 days in Kaduna. Lessons learned from the pilot phase were used to adapt survey implementation for subsequent phases: the variable skill-level of LGA level staff required a robust supervisory structure for high-quality survey implementation; long distances and difficult terrain between clusters led to reduction in the number of clusters assigned to teams per day; and GPS navigation was so useful in helping survey teams locate assigned clusters that a full time GPS specialist was hired to be part of the control center team in Abuja for phases 1 and 2. In October 2014, prior to phases 1 and 2, a 3-day refresher training of trainers was conducted in Abuja. This was followed by a 2-day cascade training for interviewers in each LGA conducted by master trainers who had participated in the previous phase.

Each survey team completed 10 clusters within 5 days (an average of two clusters per team per day), for a total of 30 clusters completed by three teams per LGA. Clusters found to lack settlements, contain fewer than seven eligible children, or be inaccessible due to security risk were not replaced or combined with other clusters. Households were visited up to two times in attempts to enroll eligible children, and reasons for non-response were documented. After obtaining verbal consent, the pre-tested, standardized paper questionnaire (S1 Appendix) was administered to the caretaker (or a knowledgeable adult, if the mother was unavailable) of the child in the selected household. Questionnaires were written in both English and Hausa, and interviews were conducted in the appropriate language for each household. Questions included socio-demographic information, RI vaccination history, awareness of RI service opportunities, use of RI services (any vaccination at health clinic or health outreach program), and reasons for non-vaccination. Vaccination status was first assessed based on caretaker’s recall, and second, based on vaccination card.

A high supervisory ratio (i.e., one team supervisor per one interview team, and one senior supervisor per three interview teams) was employed to ensure survey implementation was of the highest quality. Standardized monitoring forms were used by all supervisors in the field to assess adherence to survey protocol and data quality. To reduce selection bias of households in the field, each team was equipped with an Android™ device loaded with base maps and global positioning system (GPS) coordinates of their pre-assigned cluster start points for offline navigation. GPS coordinates were also taken at the beginning and end of fieldwork in each cluster to verify that the cluster was visited.

### Data Analysis

Data from completed paper forms were singly entered into an electronic database in Abuja using Census and Survey Processing System (CSPro) software (Version 5.0, U.S. Census Bureau). Both data cleaning and all data management were conducted using CSPro and SAS (Version 9.3, SAS Institute, Cary, North Carolina). Data cleaning included reviewing of the completeness and validity of data entered, making necessary corrections based on review of paper questionnaires, and excluding ineligible children based on date of birth (birthdates within 4 weeks of the eligible dates were allowed to account for the possibility of inaccurate documentation). For each phase, data entry and cleaning began during the survey period, and was completed immediately after field work ended; data analysis was completed at the end of the pilot phase, and then after Phase 2.

Within each LGA, data were analyzed using SAS to account for the cluster sampling, but the sample was assumed to be self-weighting. Vaccination coverage estimates and 95% confidence intervals (CIs) were calculated; modified Wilson (score) CIs are reported for estimates ≥80% and ≤20% and Wald CIs are reported for all others. Graphics were produced in R v3.2 [13]. Since LGAs were selected purposively within states, LGA data were not pooled to obtain state-level estimates. To provide a succinct summary of results, the LGA-level coverage median and range across all LGAs are presented. All coverage estimates represent a combination of data from vaccine cards and caretakers’ recall. OPV and measles coverage did not include vaccine doses given during SIAs. “Complete vaccination coverage” was defined in accordance with NPHCDA policy as receipt of the following eight vaccine doses: BCG, OPV 1/2/3, DPT 1/2/3, and measles. Reasons for not receiving vaccinations through RI services were grouped into categories of similar response choices. As part of our analysis, we compared DPT3 coverage estimates from our surveys of children aged 12–23 months during October 2014 and January 2015 to administrative DPT3 vaccination coverage for 2013 among children aged <12 months reported through the District Vaccine Data Management Tool (DVD-MT) at WHO.

## Results

The pilot phase was conducted in two LGAs in Kaduna state in May 2014. Phase 1 was conducted in October 2014 and included survey implementation in 19 LGAs in four states (Katsina, Jigawa, Sokoto and the FCT); and Phase 2 was conducted in January 2015 in 19 LGAs in four additional states (Bauchi, Kaduna, Kebbi, and Zamfara). Of the 1,200 clusters visited during the surveys in 40 LGAs, 26 clusters (2%) were excluded due to local security issues, and 5 clusters (<1%) were excluded because they lacked household structures (settlement had been relocated post-cartography). In addition, 43 clusters (4%) were included which had fewer than the target of seven households with eligible children (number of eligible children ranged 1–6; median of 5 per cluster). The average number of respondents per cluster in each LGA is described in S2 Appendix. Clusters reported to have fewer than seven eligible children were accepted only after verification of number of household structures by central staff using GPS information and satellite imagery. After excluding children based on age ineligibility (190 children), 7,815 children from 1,169 clusters in 40 LGA surveys were included in the analysis. A total of 573 households had more than one eligible child, which required survey teams to use a random number table to select one child for inclusion in the survey. Revisits were conducted to 74 households found to have no caretaker at home during the initial visit.

As shown in Table 1, the median percentage of male children was 49% (range: 43%–59%). For the highest level of education among mothers/caretakers and heads of households, Quranic schooling had the highest median percentage (57% and 48%, respectively). Residence in urban or rural areas varied widely within each LGA, but the median percentage of households residing in rural areas was 82% compared to 22% for urban areas across all LGAs.

**Table 1:** Range of socio-demographic profiles across 40 local government areas (LGAs) in Northern Nigeria–Routine Immunization Coverage Survey, 2014–2015; N = 7815

	RANGE (%)		MEDIAN (%)
**Sex of child**			
Male	43–59		49
Female	42–57		51
**Number of children in household**			
1	11–44		19
2–4	37–66		50
5+	9–41		29
**Highest level of education for mother/caretaker**			
None	1–60		11
Primary	1–25		11
Secondary	1–54		7
Post-secondary	1–36		2
Quranic schools	2–93		57
**Highest level of education of the head of household**			
None	1–26		4
Primary	1–23		9
Secondary	3–44		17
Post-secondary	2–51		15
Quranic schools	2–89		48
**Location of residence**			
Rural	17–100		82
Urban	3–83		22

**Fig 1:**
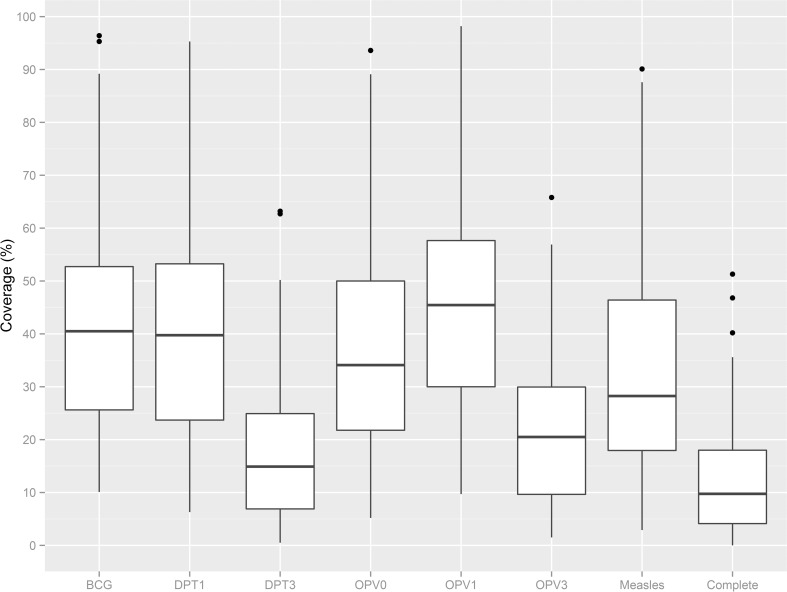
Box-and-whisker plots of routine immunization coverage by vaccination card and recall, across 40 local government areas (LGAs)–Northern Nigeria, 2014–2015. Estimates of vaccine coverage do not include OPV or measles doses given during supplemental immunization activities. Complete vaccination coverage is defined as receiving eight antigens [Bacille Calmette-Guérin (BCG), Oral Poliovirus (OPV) 1/2/3, Diphtheria-pertussis-tetanus (DPT) 1/2/3, and measles). For boxplot interpretation: the dark line is the median; “hinges” are the top and bottom of the box. The upper and lower "hinges" correspond to the first and third quartiles (25^th^ and 75^th^ percentiles). The upper and lower whiskers represent +/- 1.5 * IQR, where IQR is the inter-quartile range, or distance between the first and third quartiles. Data beyond the end of the whiskers are outliers and plotted as points (as specified by Tukey).

**Fig 2:**
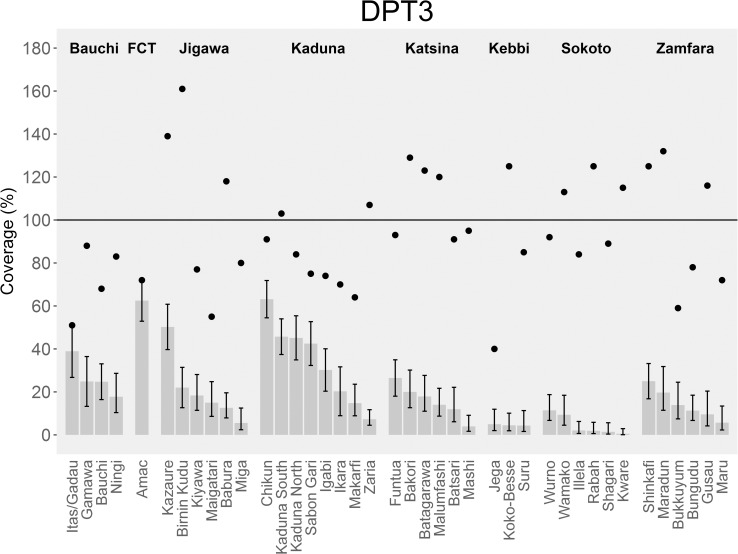
Coverage estimates with third dose of diphtheria-pertussis-tetanus vaccine (DPT3) across 40 local government areas (LGAs), by state–Northern Nigeria, 2014–2015. LGAs are grouped by state to illustrate variability in coverage across LGAs within the same state. This data is not representative of state-level coverage since LGAs were purposefully selected. Black vertical lines depict 95% confidence intervals. For comparison, administrative coverage for each LGA (source: World Health Organization, Nigeria; DVD-MT data, 2013) is represented with a black dot.

Coverage estimates of routine vaccinations ranged widely across all LGAs (Fig 1 and S3 Appendix). Median coverage for all vaccines, as well as the proportion of children with complete vaccination was below 50%. Median OPV1 coverage was highest at 44% (range: 10%–98%), and median DPT3 coverage was lowest at 14% (range: 1%-–63%). Coverage estimates also ranged widely between LGAs within the same state, as shown for DPT3 (Fig 2 and S3 Appendix).

The reported use of RI services also varied widely; by LGA, median use was 47%, with a range of 13% to 98% (Table 2). Among those who ever used RI services, the median proportion of mothers/caretakers who reported receiving a vaccine card was 87%; among those who reported receiving a card, the median proportion with available cards was 45%. For children not receiving all the recommended vaccinations through RI services, the highest median responses were reasons attributed to lack of knowledge or education (50%), followed by mother forgetting or being too busy (16%), and suboptimal access to RI services (15%) (Table 2).

**Table 2:** Reported access to routine immunization (RI) services and reasons for non-vaccination, across 40 local government areas (LGAs)–Northern Nigeria, 2014–2015.

**Access to routine immunization (RI) services, including vaccination card**	**RANGE (%)**	**MEDIAN (%)**
Reported use of RI services^1^	13–98	47
Reported receipt of a vaccination card ^2^	40–98	87
Card availability^3^	20–75	45
**Primary reasons for not receiving all vaccinations**^**4**^ **through RI services**	**RANGE (%)**	**MEDIAN (%)**
Knowledge/ education^5^	12–78	50
Mother forgot/ too busy/ plan to do it later	2–37	16
Access to RI services^6^	1–36	15
Concurrent illness/ potential side effects^7^	1–32	9
Don’t know/ missing response	2–26	4

^1^denominator = all eligible children; ‘RI services’ defined as any vaccination at health clinic or health outreach program

^2^denominator = ever use of RI

^3^denominator = ever received vaccine card; required interviewer to see card

^4^asked of all participants who responded ‘no’ to any RI and ‘no’ to receiving all vaccinations; questionnaire responses combined into categories; participants required to provide a single response.

^5^ included the following response choices: unaware of place/time of vaccination, unaware of need for vaccination, did not know needed other vaccines, feel vaccination not important, do not trust vaccines, cultural/religious reasons, husband/head of household won’t allow

^6^ included the following response choices: place of vaccination too far/difficult, time of vaccination inconvenient, unable to pay for vaccination services, unable to pay for transport, vaccine not available at facility, vaccinator absent, long waiting line, poor attitude of health workers, health outreach not regular

^7^ included following response choices: fear of side effect/adverse effect, family problem like illness of mother, child ill/mother refused, child ill/vaccinator refused

The highest median response by LGA for main information source about RI services was loud speaker/town announcer (43%), followed by health worker (21%) and radio (15%) (Table 3). We also asked about the main type of information needed to help mothers/caretakers decide to vaccinate children through RI services (Table 3). Knowing the safety of the vaccine and the importance of vaccination had the highest median responses (37% and 23%, respectively), but also the widest ranges. Knowing which diseases the vaccine protected against had a 10% median response.

**Table 3:** Source and type of information about routine immunization services, across 40 local government areas (LGAs)–Northern Nigeria, 2014–2015.

**Main information source about routine immunization services**^**1**^	**RANGE (%)**	**MEDIAN (%)**
Loud speaker/town announcer	1–77	43
Health worker	1–81	21
Radio	2–41	15
Polio campaign vaccinators	1–18	4
Husband/family/neighbor/friends	1–10	3
Community leader	1–18	3
Not heard of routine immunization before	1–14	3
Don't know	1–27	2
Community mobilizer/VCM	1–14	2
Television	1–11	1
Mosque/church	1–7	1
Women's groups	1–2	1
Mobile telephone/SMS	1	1
Poster/banner	1	1
Newspapers/magazines	1	1
**Main information needed to help decide to vaccinate children through routine immunization services**^**1**^	**RANGE (%)**	**MEDIAN (%)**
Safety of vaccine	3–77	37
Why vaccinating my child is important	3–76	23
What diseases vaccines protect against	1–30	10
Side effects of the vaccine	1–25	5
Whether my husband/family approves	1–44	4
Time/place for routine vaccination near my home	0–47	2
Schedule for vaccination of child	1–10	2
If sick children can receive the vaccine	1–8	1
Other	1–13	1
Whether my community leader approves	0–3	1
Whether my religious leader approves	0–3	1

^1^ Participants required to provide a single response.

When comparing administrative DPT3 coverage data reported for 2013 to the DPT3 coverage estimates obtained during our survey by respective LGA, we found that the administrative estimates were higher than the survey estimates for all 40 LGAs (Fig 2 and Table 4). The difference in survey vs. administrative coverage ranged from 9% in Abuja Metropolitan Area Council (AMAC) LGA, Abuja FCT to 139% in Birnin Kudu LGA, Jigawa state. Reported administrative coverage fell within the 95% CI of our survey estimate in only 2 LGAs; AMAC LGA, Abuja FCT and Itas/Gadau, Bauchi state.

**Table 4:** Administrative coverage estimates vs coverage survey estimates for third dose of diphtheria-pertussis-tetanus vaccine (DPT3).

State	Local Government Area (LGA)	Administrative estimates^1^	Routine immunization coverage survey estimates, with 95% Confidence Interval^2^	% difference between administrative and survey point estimate
**JIGAWA**	BIRNIN KUDU	161	22 (13–31)	139
**SOKOTO**	RABAH	125	2 (1–6)	123
**KEBBI**	KOKO-BESSE	125	5 (2–10)	121
**SOKOTO**	KWARE	115	1 (0–3)	115
**ZAMFARA**	MARADUN	132	20 (11–32)	112
**KATSINA**	BAKORI	129	20 (13–30)	109
**ZAMFARA**	GUSAU	116	10 (4–20)	106
**KATSINA**	MALUMFASHI	120	14 (9–22)	106
**JIGAWA**	BABURA	118	13 (8–20)	105
**KATSINA**	BATAGARAWA	123	18 (11–28)	105
**SOKOTO**	WAMAKO	113	9 (5–18)	104
**ZAMFARA**	SHINKAFI	125	25 (17–33)	100
**KADUNA**	ZARIA	107	7 (5–12)	100
**KATSINA**	MASHI	95	4 (2–9)	91
**JIGAWA**	KAZAURE	139	50 (40–61)	89
**SOKOTO**	SHAGARI	89	2 (0–6)	87
**SOKOTO**	ILLELA	84	2 (1–6)	82
**KEBBI**	SURU	85	4 (2–11)	81
**SOKOTO**	WURNO	92	11 (7–19)	81
**KATSINA**	BATSARI	91	12 (6–22)	79
**JIGAWA**	MIGA	80	6 (2–13)	74
**ZAMFARA**	BUNGUDU	78	11 (7–19)	67
**KATSINA**	FUNTUA	93	27 (18–35)	66
**ZAMFARA**	MARU	72	6 (2–13)	66
**BAUCHI**	NINGI	83	18 (10–29)	65
**BAUCHI**	GAMAWA	88	25 (13–37)	63
**JIGAWA**	KIYAWA	77	18 (11–28)	59
**KADUNA**	KADUNA SOUTH	103	46 (37–54)	57
**KADUNA**	IKARA	70	20 (9–32)	50
**KADUNA**	MAKARFI	64	15 (9–24)	49
**ZAMFARA**	BUKKUYUM	59	14 (7–25)	45
**KADUNA**	IGABI	74	30 (20–40)	44
**BAUCHI**	BAUCHI	68	25 (16–33)	43
**JIGAWA**	MAIGATARI	55	15 (9–25)	40
**KADUNA**	KADUNA NORTH	84	45 (35–55)	39
**KEBBI**	JEGA	40	5 (2–12)	35
**KADUNA**	SABON GARI	75	42 (32–52)	33
**KADUNA**	CHIKUN	91	63 (55–72)	28
**BAUCHI**	ITAS/GADAU	51	39 (27–51)	12
**ABUJA FCT**	AMAC	72	63 (53–72)	9

^1^ World Health Organization, Nigeria; DVD-MT data, 2013; target population: children < 12 months

^2^ Coverage surveys conducted in October 2014 and January 2015; target population: children 12–23 months at time of survey; the intra-class correlations (ICC) due to cluster sampling for DPT3 ranged from 0 to 0.5, with a median of 0.2.

## Discussion

Our survey findings highlight gaps in vaccination coverage at the LGA level in areas of northern Nigeria that are high risk for polio. In the LGAs surveyed, median DPT3 coverage was 14%, with all 40 LGAs having coverage below the district-level target of 80% and all at or below the national reported administrative coverage of 63% [2, 5]. While some LGAs were estimated to have coverage >80% for BCG (4 LGAs), DPT1 (2), OPV0 (2), OPV1 (3), and measles (2), the median coverage for all individual antigens across all LGAs was still <50%. Substantial differences in DPT3 coverage by survey compared with administrative reporting were observed in 38 (95%) LGAs, consistent with the findings from state-level coverage surveys [7, 8]. Our results reinforce the limitation of using administrative vaccination coverage to assess RI service delivery performance in these settings [7].

We observed a wide range in routine vaccination coverage for all antigens across the LGAs surveyed (e.g., DPT3 coverage ranging 1%—63%) and even among LGAs within the same state, similar to the results of a 2014 coverage survey completed in Kano state where DPT3 coverage by LGA ranged from 9%–80% (Dale Rhoda, KANRICS Task Force and Biostat Global Consulting, personal communication). These findings emphasize the need for LGA-level program evaluation to identify specific programmatic issues and appropriately target improvement in the respective LGAs.

Despite varying results among LGAs, the overall survey findings point to both factors of supply and demand in contributing to low vaccination coverage in Nigeria, as seen in other weak health systems [14]. Lack of knowledge or education about vaccines and vaccination services was the most commonly reported reason for non-vaccination, and knowledge about the safety and importance of vaccines were identified as the most important types of information needed to help mothers/caretakers decide to vaccinate their children. These findings highlight the need for targeted social mobilization strategies to increase the demand for RI services. The low reported use of RI services and the common identification of issues relating to access to RI services as a reason for non-vaccination (i.e., inconvenient location and time of vaccination, vaccine stock outs, inconsistent outreach schedules, and inability to pay for vaccination services) suggest more can be done to improve vaccine delivery mechanisms.

Significant strengths of this survey are that LGA-level data was gathered, analyzed, and shared for program action in LGAs designated as high-risk for polio. Using an efficient and rapidly deployable human resource infrastructure that exists within NSTOP and NFELTP, we were able to assure high quality data collection while conducting multiple concurrent LGA surveys. Local NSTOP and government immunization staff had the first-hand opportunity to assess issues contributing to poor vaccination coverage within their LGAs and inform prioritization and intensification of resources for RI service delivery. Detailed reports of survey results for individual LGAs were disseminated within 6 months to each LGA immunization program team for use in work plans for targeted improvement (S4 Appendix).

The survey analyses had limitations. Since we purposively sampled high-risk LGAs within eight states, we expected low vaccination coverage and cannot generalize results to larger geographic areas. Additionally, the sampling of children within clusters was not random, therefore enrolled households may not be representative of the cluster. The selection process, and the assumption of a self-weighting sample likely yields biased point estimates and an underestimate of the standard errors. However, it is unlikely that bias would explain the large discrepancy between administrative coverage and the survey estimates. Challenges during field operations that led to an overall decrease in sample size included: clusters with fewer than seven eligible households, clusters with insecurity issues prohibiting field work (including early withdrawal of survey teams before completing some clusters), and inaccurate estimation of children’s age during enrollment that led to exclusion based on ineligible age during analysis. The surveys were not powered for sub-population analysis, so inferential associations cannot be made about factors associated with low coverage. Overall card availability was low (median value of 45%), and reliance on maternal recall in the absence of a vaccination card can be unreliable and lead to over- or under-estimation of coverage estimates [15]. Future surveys could incorporate verification of vaccination status from health facility records, if available, to improve the documentation of vaccination status.

## Conclusions

Vaccine preventable diseases continue to contribute significantly to the morbidity and mortality burden in Nigeria. Given the limitations of using administrative vaccination coverage data and the heavy resource demand required to conduct regular household coverage surveys, practical means of gathering high quality RI data are needed to inform program planning at the LGA level. Our approach could serve as an example of efficient human resource utilization for conducting quality LGA-level coverage surveys, particularly in countries with sub-optimal vaccine coverage and heterogeneous populations similar to Nigeria. We recommend using coverage surveys as capacity building exercises for immunization staff where possible, while incorporating the improved sampling methodology outlined in the recently updated WHO cluster survey guidelines [16], to achieve high quality results.

## Supporting Information

S1 Appendix Nigeria Routine Immunization Coverage Survey Questionnaire, Phase 1 For reference, this is an example of a blank questionnaire used during Phase 1. (PDF)

S2 Appendix Data tables of antigen coverage estimates, by LGA (PDF)

S3 Appendix Graphs of routine immunization coverage estimates for each LGA, grouped by antigen All coverage estimates combine maternal recall + vaccine card data; complete coverage = 8 antigens (BCG, OPV 1, DPT 1, OPV 2, DPT 2, OPV 3, DPT 3, Measles); does not include OPV or measles doses from SIAs. The order of the LGAs within a state is based on DPT3 coverage. LGAs are grouped by state to illustrate variability in coverage across LGAs within the same state. This data is not representative of state-level coverage since LGAs were purposefully selected. **Graphs of routine immunization coverage estimates, grouped by state.** All coverage estimates combine maternal recall + vaccine card data; complete coverage = 8 antigens (BCG, OPV 1, DPT 1, OPV 2, DPT 2, OPV 3, DPT 3, Measles); does not include OPV or measles doses from SIAs.
(PDF)

S4 Appendix Survey results shared with Maru LGA immunization staff. This is an example of the slide sets that were distributed to each LGA after data analysis was completed.
(PDF)
