# Supplementary material for: Routine Vaccination Coverage in Northern Nigeria: Results from 40 District-Level Cluster Surveys, 2014-2015
Source: PLoS One. 2016 Dec 9;11(12):e0167835. doi: 10.1371/journal.pone.0167835 (PMC5148043; doi:10.1371/journal.pone.0167835)
Supplement: S1 Appendix — (PDF) [file pone.0167835.s001.pdf]

Form 1: Takadda Tambayoyi Bincike Kan Rigakafin Yau Da Kullum a Nijeriya

Nigeria Routine Immunization Coverage Survey Questionnaire, Phase 1

Jiha
State name

Lambar karamar hukuma
LGA number

__ __ __

Sunan karamar hukuma
LGA name

Sunan wuri
Locality name

Lambar unguwa
Cluster number

__ __

Sunan unguwa
Cluster name

Yanayin unguwa

[__] Birni / Urban
[__] Karkara / Rural

□ Birni / Urban
Cluster
type

□ Karkar / Rural

Lambar takardar neman bayani
Questionnaire number

__ [1–7]

Lambar kungiyan masu ganawa
Interviewer team number

__ __

Sunan mai ganawa
Interviewer names

Lambar sufabaiza
Supervisor number

__

Sunan sufabaiza
Supervisor name

Ranan ganawa
Date of interview

__ __ / __ __ / __ __ [Rana/wata/shekara]/[Day / Month / Year]

Matsayin gida a ganawa ta karshe [kungiya ta duba 1] / Household final interview status [CHECK ONE]

[__] An samu cikkaken ganawa/ Complete interview
[__] Ba'a samu chikkaken ganawa ba/ Partial interview

Supervisor's questionnaire quality control check **Signature/date**:____________________________

[__] Consent collected

[__] Child selected using random number table

[__] Child was 12-23 months old

[__] Questionnaire complete

[__] All answers legible

[__] Dates filled correctly

[__] Only one answer per question

[__] Skip patterns used properly

Neman izinin masu gida

Household consent

"Ina kwana/ Ina wuni/ Ina yini sunana………………………………… na zo a madadin sashin Kula da kiwon lafiya ta Karamar hukuma don in gudanar da bincike akan rigakafin yara. Bayanan da zamu tara, za a yi amfani da su don inganta rigakafin a karamar hukumar……………………………. Gidan ka kuma ta samu shiga.Za mu so mu yi maka tamboyoyi akan gidanka da yanayin rigakafin 'ya 'yan ka. Zai dauke mu kamar minti 30 mu kammala. Ba tilas bace a amsa tambayoyin nan kuma duk bayanan da za a bamu zasu kasance sirri ce. Ko akwai wasu tambayoyi game da binchiken?" [IN DA TAMBAYA KO KARIN BAYANI SAI AYI.]

"Good morning/afternoon/evening, my name is .... I am here on behalf of the LGA health department to conduct a survey about child immunization. The information gathered will be used to improve the immunization program in ……………. LGA. Your household was chosen randomly. We would like to ask you questions about the immunization status of children in your household. It will take about 30 minutes to complete. Participation is voluntary, and all information you provide will remain confidential. At this time, do you want to ask me any questions about the survey?"[ANSWER ANY QUESTIONS AND ADDRESS ANY CONCERNS.]

"Zamu iya fara ganawan yanzu?"

"May I begin the interview now?"

[__] EY,AN BADA IZINI. ⇨ CIGABA DA GIDAN DA YA CHANCHANTA, NAN KASA.

YES, PERMISSION IS GIVEN.⇨CONTINUE TO HOUSEHOLD ELIGIBILITY BELOW.

IN BA'A BADA IZINI BA, A TSAYAR DA GANAWAR.⇨ RUBUTA RASHIN AMINCEWA A TAKADAR UNGUWAR A KUMA SAKE YIN AMFANI DA WANNAN TAKARDAN NEMAN BAYANI A GIDAN DA CHANCHANTA NA GABA.

IF PERMISSION IS NOT GIVEN, STOP THE INTERVIEW. ⇨RECORD REFUSAL ON THE CLUSTER TRACKING FORM AND RE-USE THIS SAME QUESTIONNAIRE FOR THE NEXT ELIGIBLE HOUSEHOLD.

Gidan da ya chanchanta

Household eligibility

"Akwai yaran da ke da shekara daya amma kasa da shekaru biyu da haifuwa a cikin gidan nan?"

[YARAN DA AKA HAIFA A TSAKANIN NOWAMBA, 2012 ZUWA OCTOBER, 2013 SUNE SUKA CHAN-CHANTA.]

"Are there any children living in this household who are 1 year old but still less than 2 years old?"

[ELIGIBLE CHILDREN ARE BORN BETWEEN NOVEMBER 2012 AND OCTOBER 2013]

[__] EY, AKWAI YARO/YARINYA DA TA/YA CHANCHANTA.⇨ FARA GANAWA DA TAMBAYA H1 DAKE SHAFI NA GABA.

YES, HAVE ELIGIBLE CHILD.⇨BEGIN THE INTERVIEW WITH QUESTION H1 ON THE NEXT PAGE.

IN A'A NE SAI A DENA GANAWA.⇨A RUBUTA GIDAN BA TA CHANCHANTA BA A TAKARDA UNGUWAR A KUMA SAKE YIN AMFANI DA WANNAN TAKARDAN NEMAN BAYANI A GIDAN DAYA CHANCHANTA NA GABA.

IF NO, THE INTERVIEW IS OVER. ⇨RECORD HOUSEHOLD IS NOT ELIGIBLE ON CLUSTER TRACKING FORM AND RE-USE THIS SAME QUESTIONNAIRE FOR THE NEXT ELIGIBLE HOUSEHOLD.

BAYANI A KAN MEMBOBIN GIDA/HOUSEHOLD MEMBERSINFORMATION

"Yanzu i na so in yi maku tambayoyi a kan membobin gidan nan."
"Now, I would like to ask you some questions about the members of this household."

H1	**Mene ne sunan maigidan nan?**<br>What is the name of head of household?	

H2 **Mene ne sunayen duka yaranda ke cikin gidan, wanda suke da shekara daya amma kasa da skekaru biyu da haifuwa da sunan mahaifiyar su ko mai kula da su?**
[*Bincika*: "Matan aure nawa ne a cikin gidan?"]

What are the names of all the children in the household who are 1 year old but less than 2 years and the mothers/caregivers? [*Probe*: "How many wives are there in the household?"]

LIST THE CHILDREN FROM OLDEST TO YOUNGEST:

**Lamba**<br>Number	**Sunan Yaro/yarinya**<br>Child's name	**Sunan mahaifiya/mai kullawa**<br>Mother/caregiver's name
1		
2		
3		
4		
5		
6		
7		
8		
9		

H3 **[In yaran da suke da shekara daya amma kasa da shekaru biyu da haifuwa sun fi daya, sai a zabi yaro/yarinya ta hanyar yar cinke. In za a yi amfani da shi, sai a duba yawan yaran da suka chanchanta a sa maki a layin gefen hagu mai calar kore. Sai a kuma sa maki a lambar tambaya dake sama mai calar kore. Nema inda al'amudi da layi suka hadu. Wanan lambar inta zata zama lambar da za a yi amfani da ita a wurin yar cinke sunayen yara da aka rubuta a H2.]**

[If there is more than one child who is 1 year old but less than 2 years, select one using the random number table. To use it, circle the total number of eligible children in the grey column on the left. Then, circle the *questionnaire number* in the grey row on the top. Find the box where this column and row intersect. That number will be your randomly chosen number for the children listed in H2.]

**Random Number Table**		**Lanban takardan tambayoyi**/ Questionnaire Number						
		1	2	3	4	5	6	7
**Lamban yaran da suka chanchanta**<br>Number of Eligible Children	2	**2**	**1**	**1**	**2**	**1**	**2**	**2**
	3	**2**	**3**	**3**	**2**	**1**	**1**	**2**
	4	**4**	**2**	**1**	**3**	**2**	**1**	**4**
	5	**2**	**1**	**5**	**4**	**2**	**3**	**5**
	6	**5**	**1**	**6**	**3**	**2**	**4**	**6**
	7	**4**	**7**	**1**	**5**	**3**	**6**	**2**
	8	**1**	**3**	**4**	**1**	**8**	**1**	**7**

		9	**9**	**5**	**8**	**4**	**7**	**2**	**7**
		10	**8**	**1**	**7**	**3**	**9**	**5**	**10**
	[**A RUBUTA LAMBAR YARON DA AKA ZABA ANAN. IN YARO DAYA NE, SAI A RUBUTA 1.**] _____<br>[WRITE NUMBER OF THE SELECTED CHILD HERE. IF ONLY ONE CHILD, WRITE ‘1’.]								

**BAYANI AKAN YARO/YARINYA**/ CHILD INFORMATION	
**"Yanzu zan so in yi tambayoyi game da <Sunan yaro>. Ko zan iya Magana da mahaifiyarsa ko mai lura dasu?"** [In mahaifansa basa nan, sai a sami matashi mai a kalla shekaru 15 wanda ke da masaniyan tarihin rigakafin yaron.]<br>"Now, I would like to ask some questions about <Child's name>. May I speak with <Mother/caregiver's name>?" [If she/he is unavailable, ask if there is an adult who is at least 15 years of age and is knowledgeable about the child's vaccination history.]	
C1	**Wace rana aka haifi<Sunan yaro>?** [*Bincika*: "Wace rana, wata, da shekara da aka haife shi?" "Yaushe ne take/yake cika shekara?" Yi amfani da mahimman lokuta Kaman su, kafin ko bayan azumi, damina ko kaka.]<br>When was <Child's name>born? [*Probe:* "What day, month, and year was child born?""What is his/her birthday?" Use significant events such as before or after fasting, rainy or dry season.]<br>**Rana(RR)** / Day(DD) __ __ [In ba'a san rana ba a rubuta 99]/ [If day is unknown, write 99]<br>**Wata (WW)** / Month (MM) __ __ [Ana bukatar wata da shekara]/ [Month and year are required]<br>**Shekara**/ Year [__] 2012<br>[__] 2013
C1A	**[Duba in yaro/yarinya sun chanchan ta: Haifuwan tsakanin Nuwamba, 2012 da October, 2013?]**<br>[Check if child is eligible: born <u>between November 2012 and October 2013</u>?] [__] Ey / Yes
C2	**<Suna> na miji ne ko mace ?**<br>Is <Name> a boy or a girl?<br>[__] 1. Na miji 1. Male<br>[__] 2. Mace 2. Female
**ABIN DA MAHAIFIYA /MAI KULLAWA SUKA TUNA AKAN ALLURAN RIGAKAFIN YAU DA KULLUM**<br>MOTHER / CAREGIVER RECALL ABOUT ROUTINE VACCINATION	
**"Yanzu zan so inyi tamboyoyin a kan rigakafin yau da kullun da yaro/yarinya suka karba a asibiti, ko a lokochin fadakarwan kiwon lafiya. Wanan bai hada da rigakafin da aka yi, lokocin rigakafin cutar shan inna da bakon dauro na musammam ba."**<br>**[A rubuta "EY" in mahaifiya tace an yi rigakafi ko "A'A" in tace ba'a yi ba. Ya kamata a gan tabon BCG a wajen saman hannun hagu. Rubuta "EY" in an gan alama, "A'A" in babu alama.]**<br>"Now, I would like to ask you some questions about vaccinations your child received through routine health services at the health clinic, or a health outreach program. This does not include vaccinations received during special campaigns for polio and measles."<br>[Record "YES" if mother says that vaccination was given and "NO" if she says it was not given. For BCG, scar should be visible on left upper arm. Record "YES" if scar is seen and "NO" if scar is not visible.]	
M1	**Ko<Suna>ya/ta karban rigakafin ta hanun cibiyar kiwon lafiya ta yau da kullum a rayuwar sa/ta?**<br>Has <Name> ever received any vaccination through routine health services in his/her life?<br>[__] 1.Ey ⇨ **M2** 1. Yes ⇨ **M2**<br>[__] 2. A'a ⇨ **R2, p.9** 2. No ⇨ **R2, p.9**

M2<br><br><br><br><br><br><br><br><br>M2A	**Ko <Suna> ya taba karbar alluran rigakafin cutar tarin fuka (''BCG'') a wajen saman hannun hagu ko Kafada wanda yake barin alama a haifuwa ko bayan haifuwa?** [*Bincika:* "An fi karbansa da zaran an haifu"]<br>Has <Name> ever received an injection to protect against tuberculosis ("BCG") in the left upper arm, or shoulder, which usually causes a scar? [*Probe*: "Usually received at or soon after birth"]<br>[__] 1. Ey — 1. Yes<br>[__] 2. A'a — 2. No<br>**[DUBA TABON BCG A WAJEN SAMAN HANUN HAGU.]**<br>[CHECK FOR BCG SCAR ON LEFT UPPER OUTER ARM.]<br>[__] 1. An ga alama — 1. Scar seen<br>[__] 2. Ba' a ga alama ba — 2. No scar seen
M3<br><br><br><br><br><br><br>M3A	**Ko <Suna> ya/ta taba karban allura a saman chinyar hagu, don kariya daga cutar shan inna, tetanus, tari ko diphtheria(''Penta'' ko ''DPT'')?**<br>Has <Name> ever received an injection in their left upper thigh to protect against tetanus, whooping cough, or diphtheria ("Penta" or "DPT")?<br>[__] 1. Ey — 1. Yes<br>[__] 2. A'a ⇨ **M4** — 2.No ⇨**M4**<br>**Sau nawa gaba daya aka karbi rigakafin penta/ DPT?**<br>How many total times was Penta/DPT vaccine received?<br>**Yawan lokaci _____**[A rubuta 99 in ba'a sani ba]<br>Number of times [99=Don't know]

M4	**Ko <Suna> ya taba karbar maganin digawa ta baki na (''OPV'') don kare shi daga cutar shan innaa asibiti/wurin fadakarwa?** [*Bincika*: "Ban da ranakun yakin fadarkarwan shan inna na gida da gida."]<br>Has <Name>ever received drops in their mouth to protect against polio ("OPV") at a clinic/outreach? [*Probe: "*Not during polio house-to-house campaign"]<br>[__] 1. Ey — 1. Yes<br>[__] 2. A'a ⇨**M5** — 2. No ⇨ **M5**
M4A	**An taba diga maganin cutar shan inna a asibiti/wurin fadakarwa a cikin mako biyu da haifuwa?** [*Bincika*: "Banda ranakun fadakarwan cutar shan inna na gida da gida."]<br>Were polio drops received at a clinic/outreach in the first two weeks of birth? [*Probe:* "Not during polio house-to-house campaign"]<br>[__] 1. Cikin mako 2 da haifuwa — 1. Within 2 weeks of birth<br>[__] 2. Ba'a cikin mako biyu ba/ban sani ba — 2. Not within 2 weeks of birth/don't know
M4B	**Sau nawa gaba daya aka diga ma yaro/yarinya maganin cutar shan inna a asibiti?** [*Bincika*: "Banda ranakun fadakarwa."]<br>How many total times were polio drops received at a clinic/outreach? [*Probe: "*Not during campaign"]<br>**Sau nawa**/ Number of times**________** [Rubuta 99 in ba'a sani ba]/ [99=Don't know]
M5	**Ko <Suna>ya/ta taba karban allura a chinya da haifuwa ko bayan haifuwa don kariya daga ciwon hanta ("HepB")?**<br>Has <Name> ever received an injection in their thigh at birth or soon after birth to protect against Hepatitis B ("HepB")?<br>[__] 1. Ey — 1. Yes<br>[__] 2. A'a ⇨ **M6** — 2. No ⇨ **M6**
M5A	**Sau nawa gaba daya aka karbi rigakafin ciwon hanta?**<br>How many total times was Hepatitis B vaccine received?<br>**Yawan lokaci_____** [Rubuta 99 in ba'a sani ba]<br>Number of times [99=Don't know]
M6	**Ko <Suna> ya/ta taba karban allura a saman hannun hagu dayake/take wata 9 ko fiye da haka don kariya daga ciwon bakon dauro?** [*Bincika*: "Banda lokacin fadakarwan ciwon shawara."]<br>Has <Name> ever received an injection in his/her left upper arm at age 9 months or older to prevent him/her from getting measles? [*Probe: "*Not during a measles campaign"]<br>[__] 1. Ey — 1. Yes<br>[__] 2. A'a — 2. No

M7	**[DUBA KO MAHAIFIYA TACE"EY" YA/TA KARBI DUKA RIGAKAFI A M2–M6.]**<br>[CHECK IF MOTHER SAID "YES" TO CHILD RECEIVING ALL VACCINATIONS IN M2–M6.]<br>[__] 1. Ey — 1. Yes<br>[__] 2. A'a — 2. No
M8	**Ko <Suna> ya/ta taba karban allura a saman hannuan dama da yaka/take wata 9 ko fiye da haka don kariya daga ciwon shawara?** [*Bincika*: "Banda lokacin fadakarwan shawara"]<br>Has <Name> ever received an injection in the right upper arm at the age of 9 months or older to prevent him/her from getting yellow fever? [*Probe: "*Not during yellow fever campaign"]<br>[__] 1. Ey — 1. Yes<br>[__] 2. A'a — 2. No
M9	**Kuna da takardan shaida daga asibiti na nuna cewa yaron nan ya kammala dukan rigakafi?**<br>Do you have a certificate of completion of all vaccines for this child from the health facility?<br>[__] 1. Ey, an gani — 1. Yes, seen<br>[__] 2. Ey ba'a gani ba — 2. Yes, not seen<br>[__] 3. A'a — 3. No
M10	**Katin da ake rubuta alluran rigakafin <Suna yaro>?** [*Bincika*: Nuna kati.]<br>[IN EY NE] **Ko zan iya gani?**<br>Do you have a card where <Name>'s vaccinations are written down? [*Probe:* Show sample card.]<br>[IF YES] May I see it please?<br>[__] 1. Ey, an gani ⇨ **V1** — 1. Yes, seen ⇨**V1 (NEXT PAGE)**<br>[__] 2. Ey, ba'a gani ba — 2. Yes, not seen<br>[__] 3. A'a — 3. No
M11	**Me ya sa ba shi/ta da katin alluran rigakafin yanzu haka?** [ZABI DALILI MAI KWARI DAYA.]<br>Why don't you have a vaccination card for him/her right now? [CHOOSE ONE MAIN REASON.]<br>[__] 1. Ba'a ba mahaifiya/mai kulawa kati ba — 1. Card not given to mother/caregiver<br>[__] 2. Babu kati a yanzu /wajen gida — 2. Card not available now/outside house<br>[__] 3. Kati ya bata — 3. Card lost<br>[__] 4. Ba kati a asibiti — 4. Card not available at the clinic<br>[__] 5. Ance mahaifiya/mai kullawa su biya kudin kati — 5. Mother/caregiver asked to pay for card<br>[__] 6. Yaro bai taba zuwa asibiti ba — 6. Child never went to clinic<br>[__] 7. Ban sani ba — 7. Don't know<br>[__] 8. Saura, ambata:________________________ — 8. Other, specify

SKIP PAGE 8 AND START AT QUESTION 'R1' ON PAGE 9

KATIN RIGAKAFIN YAU DA KULLUM/ ROUTINE VACINATION CARD

[A DUBA KATIN RIGAKAFIN YARO/YARINYA IDAN AN RUBUTA KWANAN WATA SAI A RUBUTA "EY" IN KUMA BABU, SAI A RUBUTA KWANAN WATAN DA AKA BADA A CIKA KWANAN WATA YANDA YA KAMATA BA, A RUBUTA 99.] [FOR EACH VACCINE, CHECK "YES" IF ANY MARK IS MADE IN THE "DATE GIVEN" COLUMN ON THE VACCINATION CARD AND "NO" IF THERE IS NO MARK. COPY "DATES GIVEN." [illegible]

	**Rigakafi**<br>Vaccine	**An bada rigakafi?**<br>Vaccine given?	**Ranan da akabada(rana/wata/shekara)**<br>Date given (DD/MM/YY)	
V1	**OPV 0 (Birth)**	[__] 1. Ey / Yes<br>[__] 2. A'a / No	__ __/__ __/__ __	[__] Ba'a rubuta rana ba<br>Missing
V2	**HepB 0 (Birth)**	[__] 1. Ey / Yes<br>[__] 2. A'a / No	__ __/__ __/__ __	[__] Ba'a rubuta rana ba<br>Missing
V3	**BCG**	[__] 1. Ey / Yes<br>[__] 2. A'a / No	__ __/__ __/__ __	[__] Ba'a rubuta rana ba<br>Missing
V4	**OPV 1**	[__] 1. Ey / Yes<br>[__] 2. A'a / No	__ __/__ __/__ __	[__] Ba'a rubuta rana ba<br>Missing
V5	**Penta 1 / DPT 1**	[__] 1. Ey / Yes<br>[__] 2. A'a / No	__ __/__ __/__ __	[__] Ba'a rubuta rana ba<br>Missing
V6	**OPV 2**	[__] 1. Ey / Yes<br>[__] 2. A'a / No	__ __/__ __/__ __	[__] Ba'a rubuta rana ba<br>Missing
V7	**Penta 2 / DPT 2**	[__] 1. Ey / Yes<br>[__] 2. A'a / No	__ __/__ __/__ __	[__] Ba'a rubuta rana ba<br>Missing
V8	**OPV 3**	[__] 1. Ey / Yes<br>[__] 2. A'a / No	__ __/__ __/__ __	[__] Ba'a rubuta rana ba<br>Missing
V9	**Penta 3 / DPT 3**	[__] 1. Ey / Yes<br>[__] 2. A'a / No	__ __/__ __/__ __	[__] Ba'a rubuta rana ba<br>Missing
V10	**Measles (9 months)**	[__] 1. Ey / Yes<br>[__] 2. A'a / No	__ __/__ __/__ __	[__] Ba'a rubuta rana ba<br>Missing
V11	**Yellow fever**	[__] 1. Ey / Yes<br>[__] 2. A'a / No	__ __/__ __/__ __	[__] Ba'a rubuta rana ba<br>Missing
V12	**HepB 1 (10 weeks)**	[__] 1. Ey / Yes<br>[__] 2. A'a / No	__ __/__ __/__ __	[__] Ba'a rubuta rana ba<br>Missing
V13	**HepB 2 (14 weeks)**	[__] 1. Ey / Yes<br>[__] 2. A'a / No	__ __/__ __/__ __	[__] Ba'a rubuta rana ba<br>Missing
V14	**[DUBA KO KATI YA NUNA CEWA YARO/YARINYA YA/TA KARBI DUKA RIGAKAFIN DAKE V1–V10.]**<br>[CHECK IF CARD INDICATES THAT CHILD RECEIVED ALL VACCINATIONS IN V1–V10.]<br>[__] 1. Ey — 1. Yes<br>[__] 2. A'a — 2. No			

**HALAYE / SANIN RIGAKAFIN YAU DA KULLUM**<br>ROUTINE IMMUNIZATION BEHAVIORS/KNOWLEDGE	
R1	**[DUBA M7 A SHAFI NA 7 DA KUMA. DUBA A CIKIN TAMBAYA DAYA KO KUMA TAMBOYOYIN BIYU, AMSOSHIN SU "A'A" NE?]**<br>[CHECK M7 ON P.7 FOR WHETHER ANSWER IS "YES" OR "NO".]<br>[__] 1. Daya ko biyun, amsoshin "A'A" ne ⇨ **R3** — 1. IF M7 = "YES" ⇨ **R3**<br>[__] 2. Duka ba "A'A" ⇨**R2** — 2. IF M7 ="NO" ⇨ **R2**
R2	**Me yasa <Suna> bai/bata karbi duka rigakafin sa/ta ta hanun cibiyoyin kiwon lafiya nayau da kullum ba?** [KADA A KARANTA AMSOSHI. A ZABI DALII MAI KWARI GUDA DAYA.]<br>Why did <Name> not receive all of his/her vaccinations through routine health services?<br>[DO NOT READ THE ANSWERS. CHOOSE ONE MAIN REASON BASED ON RESPONSE.]<br>[__] 1. Rashin sanin amfani rigakafi — 1. Unaware of need for vaccination<br>[__] 2. Bai/bata san suna bukatan wasu rigakafin ba — 2. Did not know needed other vaccines<br>[__] 3. Zaton Kaman rigakafi bai da mahimmanci — 3. Feel vaccination not important<br>[__] 4. Rashin yarda da rigakafi — 4. Do not trust vaccines<br>[__] 5. Tsoron sakkamako/ mai illa — 5. Fear of side effect/adverse event<br>[__] 6. Dalilan al'ada da addini — 6. Cultural/religious reasons<br>[__] 7. Rashin sanin wurin/ da lokacin rigakafi — 7. Unaware of place/time of vaccination<br>[__] 8. Wurin yin rigakafi yayinisa/wahala — 8. Place of vaccination too far/difficult<br>[__] 9. Lokacin rigakafi na da takura — 9. Time of vaccination inconvenient<br>[__] 10. Mahaifiya bata da lokaci — 10. Mother too busy<br>[__] 11. Niyar yi daga baya — 11. Plan to do it later<br>[__] 12. An manta a kai yaro/yarinya wurin rigakafi — 12. Forgot to take child for vaccination<br>[__] 13. Miji/ Maigida ba zai yarda ba — 13. Husband/head of household won't allow<br>[__] 14. Babu kudin biyan rigakafi — 14. Unable to pay for vaccination services<br>[__] 15. Babu kudin mota — 15. Unable to pay for transport<br>[__] 16. Laluran iyali,kaman rashin lafiyar mahaifiya — 16. Family problem, like illness of mother<br>[__] 17. Mahaifiya ta ki saboda rashin lafiyan yaro — 17. Child ill, mother refused<br>[__] 18. Mai rigakafi ya ki saboda rashen lafiyar yaro — 18. Child ill, vaccinator refused<br>[__] 19. Ba magani rigakafi a wurin yi — 19. Vaccine not available at facility<br>[__] 20. Mai bada rigakafi baya nan — 20. Vaccinator absent<br>[__] 21. Yawan jama'a — 21. Long waiting line<br>[__] 22. Munanan halayen maaikatan kiwon lafiya — 22. Poor attitude of health workers<br>[__] 23. Ba fadakarwar lafiya akai akai — 23. Health outreach not regular<br>[__] 24. Ban sani ba — 24. Don't know<br>[__] 25. Saura, ambata:___________________ — 25. Other, specify

R3

A ta wace hanya aka fi samun bayani akan rigakafin yau da kullum?

[KADA A KARANTA AMSOSHI, A ZABI DALILI MAI KWARI KUDA DAYA.]

What is your main source of information about vaccines offered through routine health services? [DO NOT READ THE ANSWERS. CHOOSE ONE MAIN SOURCE BASED ON RESPONSE.]

[__] 1. Ma'aikacin kiwon lafiya	1. Health worker
[__] 2. Masu rigakafin ciwon shan inna	2. Polio campaign vaccinators
[__] 3. Amsa kuwa/ masu shela	3. Loud speaker/town announcer
[__] 4. Hotunan sanarwa	4. Poster/banner
[__] 5. Rediyo	5. Radio
[__] 6. Telebijin	6. Television
[__] 7. Wayar tafi da gidan ka	7. Mobile telephone/SMS
[__] 8. Jaridu/kassidu	8. Newspapers/magazines
[__] 9. Miji/yan'uwa/makwabta/abokan arziki	9. Husband/family/neighbor/friends
[__] 10. Kungiyar mata	10. Women's groups
[__] 11. Masallatai/coci-coci	11. Mosque/church
[__] 12. Shugabanin al'umma	12. Community leader
[__] 13. Ma su hada kai al'umma/VCM	13. Community mobilizer/VCM
[__] 14. Rashin jin bayanin rigakafi daga cibiyoyin lafiya	14. Not heard of routine vaccination before
[__] 15. Ban sani ba	15. Don't know
16. [illegible]	16. [illegible]

R4

Wace bayani ne iyaye mata za su bukata don yanke shawaran yi wa yaran su rigakafi ta hannun cibiyoyi kiwon lafiya ta yau da kullum?

[KAR A KARANTA AMSOSHI. ZABI BAYANI DAYA MAI KWARI BISA GA AMSOSHIN DA AKA SAMU.]

What information would help mothers decide to vaccinate their children through routine immunization services? [DO NOT READ THE ANSWERS. CHOOSE ONE MAIN INFORMATION BASED ON THEIR RESPONSE.]

[__] 1. Ingancin rigakafi	1. Safety of the vaccine
[__] 2. Sakkamako mai illa na rigakafin	2. Side effects of the vaccine
[__] 3. Dalilin da yasa yi wa yaro na rigakafi ke da mahimminci	3. Why vaccinating my child is important
[__] 4. Wane cututtuka ne rigakafin ke karewa	4. What diseases vaccines protect against
[__] 5. Ko yara marasa lafiya za su iya karban rigakafi	5. If sick children can receive the vaccine
[__] 6. Lokacin/wurin yin rigakafin yau da kullum yayi kusa da gida na.	6. Time/place for routine vaccination near my home
[__] 7. Tsarin yi wa yaro rigakafi	7. Schedule for vaccination of child
[__] 8. Ko maigida/iyali sun ammince	8. Whether my husband/family approves
[__] 9. Ko shugaban addini na ya ammince	9. Whether my religious leader approves
[__] 10. Ko shugaban al'umma ta ya ammince	10. Whether my community leader approves
[__] 11. Saura, ambata: ____________________	11. Other, specify:

FADAKARWA AKAN CUTAR CIWON SHAN INNA/ POLIO CAMPAIGNS

“Yanzu zan so inyi wasu tambayoyi akan rigakafin da yaron ka ya karba a fadakarwa ciwon shan ina na kwanan nan. A lokachin fadakarwan, kungiyoyi masu rigakafin su kan bi gida gida suna diga maganin a bakin yara. Kungiyoyin su kan sa shaida a dan yastan yaro don ya nuna cewa ya karbe wannan rigakafin.”

“Now, I would like to ask some questions about vaccinations your child received during recent polio campaigns, or immunization plus days. During polio campaigns, vaccination teams come to houses and give polio vaccine drops in the children’s mouths. Teams also mark the child’s finger to indicate the child has already collected the vaccine.”

P1 **Ko <Suna> ya karbi rigakafin cutar shan inna da aka yi na gida gida a watan Satumba 18-23, 2014?** [*Bincika*: “A watan da ta wace.”]

Did <Name> receive polio vaccine drops during the September 18-23, 2014 house-to-house polio campaigns? [*Probe*: “Last month”]

[__] 1. Ey ⇨ **P2**	1. Yes ⇨ **P2**
[__] 2. A’a	2. No

P1A **Me yasa bai/bata karbi rigakafin cutar shan inna lokacin fadakarwan da aka yi a Satumba ba?**
Why did he/she not collect vaccine during the September polio campaign?

[A RUBUTA LAMBAR AMSOSHI DAGA JERIN SUNAYE DA KE SHAFI NA GABA] ______
[RECORD NUMBER FOR RESPONSE FROM THE LIST ON THE NEXT PAGE]

IF RESPONSE IS “19 – Other” PLEASE WRITE RESPONSE HERE: ________________________________

P2 **Ko <Suna> ya karbi rigakafin cutar shan inna da aka yi na gida gida a watan Ogusta 9-12, 2014?** [*Bincika*: “Wata biyu dasu ka wuce.”]

Did <Name> receive polio vaccine drops during the August 9-12, 2014 house-to-house polio campaigns? [*Probe*: “2 months ago”]

[__] 1. Ey ⇨ **P3**	1. Yes ⇨ **P3**
[__] 2. A’a	2. No

P2A **Me yasa bai/bata karbi rigakafin cutar shan inna lokacin fadakarwan da aka yi a Ogusta ba?**
Why did he/she not collect vaccine during the August polio campaign?

[A RUBUTA LAMBAR AMSOSHI DAGA JERIN SUNAYE DA KE SHAFI NA GABA] ______
[RECORD NUMBER FOR RESPONSE FROM THE LIST ON THE NEXT PAGE]

IF RESPONSE IS “19 – Other” PLEASE WRITE RESPONSE HERE: ________________________________

**JERIN AMSOSHI NA TAMBAYA P1A DA P2A** / RESPONSE LIST for QUESTIONS P1A and P2A	
1. Kungiyan basu zo gida ba	1. House not visited by team
2. Yaro ba shi nan	2. Child absent
3. Rashin ingancin magani/tsoron sakkamako mai illa	3. Vaccine safety/fear of adverse event
4. Zaton ba'a bukata	4. No felt need
5. Dalilin Al'ada/addini	5. Religious/cultural reasons
6. Dalilan siyasa da yanayin mulki	6. Political reasons
7. Raci akan rigakafin	7. Too many rounds
8. Rashin kula da sauran cututtuka	8. Other unmet health needs
9. Mahaifiya ta ki kuma ba dalili	9. Mother refused, no reason given
10. Miji/Maigida ya ki	10. Husband/head of household refused
11. Ma'aikata ba su zo gida ba	11. Vaccinator did not visit house
12. Yaro ba lafiya	12. Child sick
13. Mahaifiya/mai kullawa bata bada izini ba	13. No mother/caregiver consent given
14. Ba'a ji dadin ma'aikata ba	14.Unhappy with team
15. An daga sai wane lokaci	15. Postponed until another time
16. Ba'a san ana fadakarwa ba	16. Not aware of campaign
17. Rigakafin lokacin fadakarwa bai kai wadda aka bada wurin cibiyoyin kiwon lafiya inganci ba	17. Campaign vaccine is not as good as vaccine offered through routine health services
18. Ban sani ba	18. Don't know
19. Saura	19. Other

P3	**Ta wace hanya kake/kike samun bayani akan fadakarwa cutar shan inna?**<br>[KA DA A KARANTA AMSOSHI A ZABI DALILI DAYA MAI KWARI BISA GA BAYANI DA AKA SAMU.]<br>Which is your main source of information about polio campaigns?<br>[DO NOT READ THE ANSWERS. CHOOSE ONE MAIN REASON BASED ON RESPONSE.]	
	[__] 1. Ma'aikachin kiwon lafiya	1. Health worker
	[__] 2. Masu bada rigakafin cutar shan inna	2. Polio campaign vaccinators
	[__] 3. Ammsakuwa/masu shela	3. Loud speaker/town announcer
	[__] 4. Hotunan sanarwa	4. Poster/banner
	[__] 5. Rediyo	5. Radio
	[__] 6. Telebijiin	6. Television
	[__] 7. Wayar tafi da gidan ka/ gajeruwan sako	7. Mobile telephone/SMS
	[__] 8. Jaridu/ kassidu	8. Newspapers/magazines
	[__] 9.Miji/yan'uwa/Makwabta/Abokai	9. Husband/family/neighbor/friends
	[__] 10. Kungiyar mata	10. Women's groups
	[__] 11. Massallaci da coci	11. Mosque/church
	[__] 12. Masu hada kan al'umma/VCM	12. Community mobilizer/VCM
	[__] 13. Ban taba jin labarin rigakafin ta wurin ma'aikatan ba	13. Not heard of polio campaigns
	[__] 14. Ban sani ba	14. Don't know
	[__] 15. Saura, ambata:____________________________	15. Other, specify

BAYANI GAME DA MAHAIFIYA/ MOTHER'S INFORMATION		
**"Yanzu zan soin tambaye ka dan tarihin(mahaifiya /mai kullawa)."**<br>"Now, I would like to ask you some questions about your (or the mother's/caregiver's) background."		
B1	**Ya'ya nawa kike (ko maihaifiya/ mai kullawa) da shi a yanzu?**<br>How many children do you (or does the mother/caregiver) have currently?	**Lamba_____**<br>Number
B2	**Wane matakin illimin ne kika kai (maihaifiya/mai kullawa)?**<br>What is the highest level of schooling you (or the mother/caregiver) achieved?<br>[__] 1. Babu ilimi — 1. None<br>[__] 2. Ilimin firamare (Wane mataki) — 2. Primary (any amount)<br>[__] 3. Ilimin sakandare (Wane mataki) — 3. Secondary (any amount)<br>[__] 4. Ilimin gaba da sakandari — 4. Post-secondary<br>[__] 5. Makarantan Islamiyya — 5. Quranic schools	
B3	**Wane matakin makaranta mai gida ya kure?**<br>What is the highest level of schooling the head of household achieved?<br>[__] 1. Babu — 1. None<br>[__] 2. Ilimin firamare (Wane mataki) — 2. Primary (any amount)<br>[__] 3. Ilimin sakandare (Wane mataki) — 3. Secondary (any amount)<br>[__] 4. Ilimin gaba da sakandari — 4. Post-secondary<br>[__] 5. Makarantan Islamiyya — 5. Quranic schools	

[A DUBA TAKARDAR NEMAN BAYANI, KO AN AMSA DUKA TAMBOYOYI, SAI A KALMALA GANAWAR DA GODE WA MAIHAIFIYA / MAI KULLAWA SABO DA HADIN KAI DA SUKA BAYAR.]

[CHECK THE QUESTIONNAIRE FOR COMPLETION OF ALL QUESTIONS.

THEN, END THE INTERVIEW AND THANK THE MOTHER/CAREGIVER FOR PARTICIPATING.]
