# Supplementary material for: Routine Vaccination Coverage in Northern Nigeria: Results from 40 District-Level Cluster Surveys, 2014-2015
Source: PLoS One. 2016 Dec 9;11(12):e0167835. doi: 10.1371/journal.pone.0167835 (PMC5148043; doi:10.1371/journal.pone.0167835)
Supplement: S2 Appendix — (PDF) [file pone.0167835.s002.pdf]

Table 1: BCG Coverage per LGA, Nigeria 2014. Immunization Coverage Survey[1,2]										
S/No	State	Lga	Sample Size	Cluster	Average No. of respondents/ cluster	Coverage Estimate (%)	Lower 95% CI	Upper 95% CI	Design Effect	ICC
1	Sokoto	Illela	195	30	6.5	14	8	23	2.1	0.20
2	Sokoto	Wurno	210	30	7.0	26	16	36	2.5	0.26
3	Sokoto	Rabah	210	30	7.0	11	7	17	1.2	0.03
4	Sokoto	Kware	201	30	6.7	12	7	22	2.5	0.27
5	Sokoto	Shagari	206	30	6.9	10	5	19	2.3	0.22
6	Sokoto	Wamako	203	30	6.8	28	16	39	3.4	0.41
7	Katsina	Batagarawa	207	30	6.9	33	21	46	3.3	0.39
8	Katsina	Mashi	202	30	6.7	35	27	43	1.5	0.08
9	Katsina	Batsari	192	28	6.9	41	30	52	2.4	0.24
10	Katsina	Malumfashi	193	30	6.4	38	28	48	2.0	0.18
11	Katsina	Bakori	200	30	6.7	44	32	56	2.6	0.29
12	Katsina	Funtua	204	30	6.8	44	34	54	1.9	0.16
13	Jigawa	Kazaure	209	30	7.0	81	73	88	1.9	0.16
14	Jigawa	Babura	198	30	6.6	48	36	60	2.9	0.34
15	Jigawa	Maigatari	207	30	6.9	48	36	60	2.8	0.30
16	Jigawa	Miga	197	30	6.6	25	17	34	1.7	0.12
17	Jigawa	Kiyawa	180	30	6.0	60	50	70	1.6	0.12
18	Jigawa	Birnin_Kudu	177	29	6.1	45	35	55	1.7	0.13
19	FCT	Amac	192	29	6.6	96	91	99	1.7	0.13
20	Zamfara	Bukkuyum	194	29	6.7	28	16	40	3.3	0.41
21	Zamfara	Maradun	208	30	6.9	45	34	56	2.4	0.23
22	Zamfara	Shinkafi	208	30	6.9	44	35	54	1.8	0.14
23	Zamfara	Gusau	157	23	6.8	30	19	41	2.2	0.20
24	Zamfara	Bungudu	159	23	6.9	38	27	49	1.9	0.14
25	Zamfara	Maru	141	23	6.1	21	7	35	3.9	0.56
26	Bauchi	Gamawa	181	28	6.5	60	50	71	2.0	0.17
27	Bauchi	Itas-Gadau	208	30	6.9	50	38	63	3.1	0.35
28	Bauchi	Ningi	203	30	6.8	40	29	51	2.6	0.27
29	Bauchi	Bauchi	198	29	6.8	70	60	80	2.2	0.21
30	Kaduna	Makarfi	203	30	6.8	25	14	35	2.9	0.33
31	Kaduna	Ikara	207	30	6.9	39	27	51	3.0	0.34
32	Kaduna	Zaria	205	30	6.8	45	36	55	1.8	0.14
33	Kaduna	Igabi	202	29	7.0	59	47	72	3.3	0.38
34	Kaduna	Kaduna North	186	29	6.4	83	74	89	1.8	0.14
35	Kaduna	Chikun	171	25	6.8	95	87	98	2.7	0.29
36	Kebbi	Jega	200	30	6.7	18	12	26	1.8	0.14
37	Kebbi	Suru	207	30	6.9	10	6	17	1.8	0.13
38	Kebbi	Koko Besse	201	30	6.7	15	8	29	4.2	0.56
39	Kaduna	Sabo Gari	207	30	6.9	76	66	86	2.7	0.28
40	Kaduna	Kaduna South	186	30	6.4	89	81	94	1.9	0.17

1 Only clusters that were surveyed are included

2 When the design effect was estimated to be less than 1 we present it as 1, and the ICC=0.

Table 2: DPT1 Coverage per LGA,Nigeria 2014. Immunization Coverage Survey[1,2]										
**S/No**	**State**	**Lga**	**Sample Size**	**Cluster**	**Average No. of respondents/ cluster**	**Coverage Estimate (%)**	**Lower 95% CI**	**Upper 95% CI**	**Design Effect**	**ICC**
**1**	Sokoto	Illela	195	30	6.5	17	11	27	2.0	0.18
**2**	Sokoto	Wurno	210	30	7.0	24	14	34	2.8	0.30
**3**	Sokoto	Rabah	210	30	7.0	11	6	18	1.9	0.15
**4**	Sokoto	Kware	201	30	6.7	8	5	15	1.8	0.13
**5**	Sokoto	Shagari	206	30	6.9	6	3	14	2.3	0.23
**6**	Sokoto	Wamako	203	30	6.8	27	15	38	3.2	0.38
**7**	Katsina	Batagarawa	207	30	6.9	34	22	47	3.4	0.40
**8**	Katsina	Mashi	202	30	6.7	21	15	28	1.2	0.03
**9**	Katsina	Batsari	192	28	6.9	44	33	55	2.2	0.21
**10**	Katsina	Malumfashi	193	30	6.4	39	28	51	2.4	0.27
**11**	Katsina	Bakori	200	30	6.7	42	31	53	2.5	0.26
**12**	Katsina	Funtua	204	30	6.8	41	31	51	2.0	0.17
**13**	Jigawa	Kazaure	209	30	7.0	77	68	86	2.3	0.21
**14**	Jigawa	Babura	198	30	6.6	44	32	56	2.7	0.30
**15**	Jigawa	Maigatari	207	30	6.9	47	35	59	2.9	0.32
**16**	Jigawa	Miga	197	30	6.6	23	15	32	1.9	0.17
**17**	Jigawa	Kiyawa	180	30	6.0	58	48	67	1.7	0.13
**18**	Jigawa	Birnin_Kudu	177	29	6.1	52	43	61	1.5	0.10
**19**	FCT	Amac	192	29	6.6	95	88	98	2.2	0.21
**20**	Zamfara	Bukkuyum	194	29	6.7	36	23	49	3.4	0.42
**21**	Zamfara	Maradun	208	30	6.9	43	32	53	2.3	0.22
**22**	Zamfara	Shinkafi	208	30	6.9	50	41	60	1.9	0.15
**23**	Zamfara	Gusau	157	23	6.8	29	19	40	2.0	0.18
**24**	Zamfara	Bungudu	159	23	6.9	33	22	44	2.0	0.16
**25**	Zamfara	Maru	141	23	6.1	23	8	39	4.2	0.62
**26**	Bauchi	Gamawa	181	28	6.5	56	45	67	2.0	0.19
**27**	Bauchi	Itas-Gadau	208	30	6.9	52	41	64	2.8	0.30
**28**	Bauchi	Ningi	203	30	6.8	38	27	50	2.6	0.27
**29**	Bauchi	Bauchi	198	29	6.8	68	58	77	1.9	0.15
**30**	Kaduna	Makarfi	203	30	6.8	30	18	41	2.9	0.34
**31**	Kaduna	Ikara	207	30	6.9	40	29	51	2.5	0.25
**32**	Kaduna	Zaria	205	30	6.8	33	24	41	1.5	0.09
**33**	Kaduna	Igabi	202	29	7.0	57	46	69	2.8	0.30
**34**	Kaduna	Kaduna North	186	29	6.4	77	69	86	2.0	0.18
**35**	Kaduna	Chikun	171	25	6.8	94	86	98	2.1	0.18
**36**	Kebbi	Jega	200	30	6.7	20	13	30	2.2	0.21
**37**	Kebbi	Suru	207	30	6.9	13	7	22	2.4	0.23
**38**	Kebbi	Koko Besse	201	30	6.7	19	11	33	3.8	0.50
**39**	Kaduna	Sabo Gari	207	30	6.9	70	59	81	2.7	0.29
**40**	Kaduna	Kaduna South	186	30	6.4	81	73	87	1.4	0.08

1 Only clusters that were surveyed are included

2 When the design effect was estimated to be less than 1 we present it as 1, and the ICC=0.

Table 3: DPT3 Coverage per LGA, Nigeria 2014. Immunization Coverage Survey[1,2]										
**S/No**	**State**	**Lga**	**Sample Size**	**Cluster**	**Average No. of respondents/ cluster**	**Coverage Estimate (%)**	**Lower 95% CI**	**Upper 95% CI**	**Design Effect**	**ICC**
**1**	Sokoto	Illela	195	30	6.5	2	1	6	1.4	0.08
**2**	Sokoto	Wurno	210	30	7.0	11	7	19	1.8	0.13
**3**	Sokoto	Rabah	210	30	7.0	2	1	6	1.4	0.07
**4**	Sokoto	Kware	201	30	6.7	0	0	3	1.0	0.00
**5**	Sokoto	Shagari	206	30	6.9	1	0	6	1.6	0.11
**6**	Sokoto	Wamako	203	30	6.8	9	5	18	2.7	0.29
**7**	Katsina	Batagarawa	207	30	6.9	18	11	28	2.4	0.24
**8**	Katsina	Mashi	202	30	6.7	4	2	9	1.6	0.10
**9**	Katsina	Batsari	192	28	6.9	12	6	22	2.8	0.30
**10**	Katsina	Malumfashi	193	30	6.4	14	9	22	1.6	0.12
**11**	Katsina	Bakori	200	30	6.7	20	13	30	2.4	0.24
**12**	Katsina	Funtua	204	30	6.8	26	18	35	1.8	0.14
**13**	Jigawa	Kazaure	209	30	7.0	50	40	61	2.2	0.20
**14**	Jigawa	Babura	198	30	6.6	13	8	20	1.5	0.08
**15**	Jigawa	Maigatari	207	30	6.9	15	9	25	2.6	0.27
**16**	Jigawa	Miga	197	30	6.6	6	2	12	2.1	0.19
**17**	Jigawa	Kiyawa	180	30	6.0	18	11	28	2.1	0.21
**18**	Jigawa	Birnin_Kudu	177	29	6.1	22	13	31	2.2	0.23
**19**	FCT	Amac	192	29	6.6	63	53	72	1.8	0.14
**20**	Zamfara	Bukkuyum	194	29	6.7	14	7	25	2.8	0.32
**21**	Zamfara	Maradun	208	30	6.9	20	11	32	3.4	0.40
**22**	Zamfara	Shinkafi	208	30	6.9	25	17	33	1.8	0.13
**23**	Zamfara	Gusau	157	23	6.8	10	4	20	2.7	0.29
**24**	Zamfara	Bungudu	159	23	6.9	11	7	18	1.3	0.05
**25**	Zamfara	Maru	141	23	6.1	6	2	13	1.7	0.14
**26**	Bauchi	Gamawa	181	28	6.5	25	13	36	3.1	0.38
**27**	Bauchi	Itas-Gadau	208	30	6.9	39	27	51	3.1	0.35
**28**	Bauchi	Ningi	203	30	6.8	18	10	29	2.9	0.32
**29**	Bauchi	Bauchi	198	29	6.8	25	16	33	1.7	0.13
**30**	Kaduna	Makarfi	203	30	6.8	15	9	24	2.1	0.19
**31**	Kaduna	Ikara	207	30	6.9	20	9	32	3.9	0.50
**32**	Kaduna	Zaria	205	30	6.8	7	4	12	1.0	0.00
**33**	Kaduna	Igabi	202	29	7.0	30	20	40	2.2	0.20
**34**	Kaduna	Kaduna North	186	29	6.4	45	35	55	1.9	0.16
**35**	Kaduna	Chikun	171	25	6.8	63	54	72	1.3	0.05
**36**	Kebbi	Jega	200	30	6.7	5	2	12	2.2	0.21
**37**	Kebbi	Suru	207	30	6.9	4	2	11	2.4	0.24
**38**	Kebbi	Koko Besse	201	30	6.7	4	2	10	1.7	0.12
**39**	Kaduna	Sabo Gari	207	30	6.9	42	32	52	2.1	0.19
**40**	Kaduna	Kaduna South	186	30	6.4	46	37	54	1.2	0.04

1 Only clusters that were surveyed are included

2 When the design effect was estimated to be less than 1 we present it as 1, and the ICC=0.

Table 4: Measles Coverage per LGA,Nigeria 2014. Immunization Coverage Survey[1,2]										
**S/No**	**State**	**Lga**	**Sample Size**	**Cluster**	**Average No. of respondents/ cluster**	**Coverage Estimate (%)**	**Lower 95% CI**	**Upper 95% CI**	**Design Effect**	**ICC**
**1**	Sokoto	Illela	195	30	6.5	7	3	15	2.4	0.26
**2**	Sokoto	Wurno	210	30	7.0	20	11	30	2.5	0.25
**3**	Sokoto	Rabah	210	30	7.0	8	4	13	1.4	0.06
**4**	Sokoto	Kware	201	30	6.7	9	5	16	1.7	0.12
**5**	Sokoto	Shagari	206	30	6.9	3	1	9	2.3	0.21
**6**	Sokoto	Wamako	203	30	6.8	21	11	31	2.9	0.33
**7**	Katsina	Batagarawa	207	30	6.9	28	17	38	2.8	0.31
**8**	Katsina	Mashi	202	30	6.7	16	11	23	1.4	0.06
**9**	Katsina	Batsari	192	28	6.9	40	27	52	2.9	0.32
**10**	Katsina	Malumfashi	193	30	6.4	33	21	44	2.6	0.29
**11**	Katsina	Bakori	200	30	6.7	29	20	38	2.0	0.17
**12**	Katsina	Funtua	204	30	6.8	22	15	30	1.6	0.10
**13**	Jigawa	Kazaure	209	30	7.0	54	43	65	2.4	0.24
**14**	Jigawa	Babura	198	30	6.6	29	18	39	2.5	0.27
**15**	Jigawa	Maigatari	207	30	6.9	40	29	51	2.5	0.26
**16**	Jigawa	Miga	197	30	6.6	10	6	16	1.3	0.05
**17**	Jigawa	Kiyawa	180	30	6.0	39	31	48	1.2	0.04
**18**	Jigawa	Birnin_Kudu	177	29	6.1	48	38	58	1.5	0.10
**19**	FCT	Amac	192	29	6.6	88	78	93	2.4	0.24
**20**	Zamfara	Bukkuyum	194	29	6.7	26	14	37	3.1	0.37
**21**	Zamfara	Maradun	208	30	6.9	36	25	46	2.3	0.22
**22**	Zamfara	Shinkafi	208	30	6.9	46	37	55	1.6	0.10
**23**	Zamfara	Gusau	157	23	6.8	18	10	29	2.1	0.20
**24**	Zamfara	Bungudu	159	23	6.9	28	18	38	1.8	0.13
**25**	Zamfara	Maru	141	23	6.1	21	7	34	3.8	0.55
**26**	Bauchi	Gamawa	181	28	6.5	55	44	66	2.1	0.21
**27**	Bauchi	Itas-Gadau	208	30	6.9	49	37	61	2.7	0.28
**28**	Bauchi	Ningi	203	30	6.8	31	21	40	2.2	0.20
**29**	Bauchi	Bauchi	198	29	6.8	47	37	57	2.0	0.17
**30**	Kaduna	Makarfi	203	30	6.8	22	13	31	2.3	0.23
**31**	Kaduna	Ikara	207	30	6.9	26	15	36	2.9	0.32
**32**	Kaduna	Zaria	205	30	6.8	18	13	24	1.0	0.00
**33**	Kaduna	Igabi	202	29	7.0	45	32	57	3.2	0.37
**34**	Kaduna	Kaduna North	186	29	6.4	67	56	77	2.3	0.23
**35**	Kaduna	Chikun	171	25	6.8	90	81	95	2.2	0.21
**36**	kebbi	Jega	200	30	6.7	15	9	24	2.1	0.19
**37**	kebbi	Suru	207	30	6.9	10	6	18	2.0	0.17
**38**	kebbi	Koko Besse	201	30	6.7	16	8	29	4.1	0.55
**39**	Kaduna	Sabo Gari	207	30	6.9	54	42	66	2.8	0.31
**40**	Kaduna	Kaduna South	186	30	6.4	65	56	74	1.6	0.11

1 Only clusters that were surveyed are included

2 When the design effect was estimated to be less than 1 we present it as 1, and the ICC=0.

Table 5: OPV0 Coverage per LGA,Nigeria 2014. Immunization Coverage Survey[1,2]										
S/No	State	Lga	Sample Size	Cluster	Average No. of respondents/ cluster	Coverage Estimate (%)	Lower 95% CI	Upper 95% CI	Design Effect	ICC
1	Sokoto	Illela	195	30	6.5	15	9	24	2.3	0.23
2	Sokoto	Wurno	210	30	7.0	28	17	38	2.8	0.31
3	Sokoto	Rabah	210	30	7.0	5	3	10	1.1	0.01
4	Sokoto	Kware	201	30	6.7	7	4	12	1.1	0.02
5	Sokoto	Shagari	206	30	6.9	10	5	17	2.0	0.17
6	Sokoto	Wamako	203	30	6.8	23	12	33	2.9	0.33
7	Katsina	Batagarawa	207	30	6.9	27	16	38	2.8	0.31
8	Katsina	Mashi	202	30	6.7	22	15	29	1.5	0.08
9	Katsina	Batsari	192	28	6.9	34	24	44	2.1	0.19
10	Katsina	Malumfashi	193	30	6.4	36	26	47	2.2	0.22
11	Katsina	Bakori	200	30	6.7	22	14	29	1.8	0.14
12	Katsina	Funtua	204	30	6.8	34	25	43	1.7	0.13
13	Jigawa	Kazaure	209	30	7.0	76	68	84	1.9	0.15
14	Jigawa	Babura	198	30	6.6	45	32	58	3.0	0.36
15	Jigawa	Maigatari	207	30	6.9	43	31	56	3.0	0.35
16	Jigawa	Miga	197	30	6.6	26	17	35	1.8	0.15
17	Jigawa	Kiyawa	180	30	6.0	60	51	69	1.5	0.10
18	Jigawa	Birnin_Kudu	177	29	6.1	50	40	60	1.7	0.14
19	FCT	Amac	192	29	6.6	89	81	94	2.0	0.17
20	Zamfara	Bukkuyum	194	29	6.7	11	6	22	3.0	0.35
21	Zamfara	Maradun	208	30	6.9	42	32	53	2.2	0.19
22	Zamfara	Shinkafi	208	30	6.9	43	34	52	1.7	0.12
23	Zamfara	Gusau	157	23	6.8	30	19	41	2.0	0.17
24	Zamfara	Bungudu	159	23	6.9	35	24	45	1.7	0.11
25	Zamfara	Maru	141	23	6.1	24	8	40	4.6	0.69
26	Bauchi	Gamawa	181	28	6.5	56	45	67	2.2	0.21
27	Bauchi	Itas-Gadau	208	30	6.9	50	38	62	2.8	0.31
28	Bauchi	Ningi	203	30	6.8	42	33	52	1.9	0.15
29	Bauchi	Bauchi	198	29	6.8	69	60	78	1.8	0.13
30	Kaduna	Makarfi	203	30	6.8	22	12	31	2.6	0.27
31	Kaduna	Ikara	207	30	6.9	24	12	37	4.2	0.54
32	Kaduna	Zaria	205	30	6.8	33	23	43	2.1	0.18
33	Kaduna	Igabi	202	29	7.0	44	33	55	2.4	0.24
34	Kaduna	Kaduna North	186	29	6.4	77	68	86	2.0	0.18
35	Kaduna	Chikun	171	25	6.8	94	87	97	1.5	0.08
36	Kebbi	Jega	200	30	6.7	17	11	26	2.1	0.20
37	Kebbi	Suru	207	30	6.9	8	4	16	2.5	0.25
38	Kebbi	Koko Besse	201	30	6.7	6	2	15	3.0	0.35
39	Kaduna	Sabo Gari	207	30	6.9	65	54	76		
40	Kaduna	Kaduna South	186	30	6.2	80	72	88		

1 Only clusters that were surveyed are included

2 When the design effect was estimated to be less than 1 we present it as 1, and the ICC=0.

Table 6: OPV1 Coverage per LGA,Nigeria 2014. Immunization Coverage Survey[1,2]										
**S/No**	**State**	**Lga**	**Sample Size**	**Cluster**	**Average No. of respondents/ cluster**	**Coverage Estimate (%)**	**Lower 95% CI**	**Upper 95% CI**	**Design Effect**	**ICC**
**1**	Sokoto	Illela	195	30	6.5	19	12	28	2.1	0.20
**2**	Sokoto	Wurno	210	30	7.0	30	20	40	2.5	0.25
**3**	Sokoto	Rabah	210	30	7.0	14	8	22	1.9	0.16
**4**	Sokoto	Kware	201	30	6.7	12	8	20	1.6	0.11
**5**	Sokoto	Shagari	206	30	6.9	10	5	17	2.0	0.17
**6**	Sokoto	Wamako	203	30	6.8	30	18	42	3.2	0.39
**7**	Katsina	Batagarawa	207	30	6.9	33	22	45	3.0	0.34
**8**	Katsina	Mashi	202	30	6.7	35	25	44	1.9	0.16
**9**	Katsina	Batsari	192	28	6.9	48	37	60	2.3	0.22
**10**	Katsina	Malumfashi	193	30	6.4	40	29	51	2.2	0.21
**11**	Katsina	Bakori	200	30	6.7	50	38	61	2.5	0.27
**12**	Katsina	Funtua	204	30	6.8	45	35	55	2.0	0.17
**13**	Jigawa	Kazaure	209	30	7.0	80	72	88	2.0	0.16
**14**	Jigawa	Babura	198	30	6.6	51	38	63	3.0	0.36
**15**	Jigawa	Maigatari	207	30	6.9	57	44	70	3.3	0.39
**16**	Jigawa	Miga	197	30	6.6	28	20	36	1.6	0.11
**17**	Jigawa	Kiyawa	180	30	6.0	61	52	70	1.5	0.09
**18**	Jigawa	Birnin_Kudu	177	29	6.1	59	49	70	1.9	0.18
**19**	FCT	Amac	192	29	6.6	96	91	99	1.7	0.13
**20**	Zamfara	Bukkuyum	194	29	6.7	36	23	49	3.6	0.45
**21**	Zamfara	Maradun	208	30	6.9	49	39	59	2.0	0.18
**22**	Zamfara	Shinkafi	208	30	6.9	57	47	66	1.9	0.14
**23**	Zamfara	Gusau	157	23	6.8	32	22	43	2.0	0.17
**24**	Zamfara	Bungudu	159	23	6.9	38	28	49	1.8	0.14
**25**	Zamfara	Maru	141	23	6.1	29	13	45	4.1	0.60
**26**	Bauchi	Gamawa	181	28	6.5	68	58	78	2.1	0.19
**27**	Bauchi	Itas-Gadau	208	30	6.9	56	44	67	2.7	0.29
**28**	Bauchi	Ningi	203	30	6.8	49	39	60	2.1	0.19
**29**	Bauchi	Bauchi	198	29	6.8	72	63	82	2.1	0.20
**30**	Kaduna	Makarfi	203	30	6.8	31	19	42	3.0	0.34
**31**	Kaduna	Ikara	207	30	6.9	43	31	54	2.7	0.29
**32**	Kaduna	Zaria	205	30	6.8	46	37	56	1.7	0.12
**33**	Kaduna	Igabi	202	29	7.0	57	44	70	3.4	0.40
**34**	Kaduna	Kaduna North	186	29	6.4	82	73	88	1.8	0.15
**35**	Kaduna	Chikun	171	25	6.8	98	93	100	1.7	0.12
**36**	Kebbi	Jega	200	30	6.7	26	15	36	2.8	0.31
**37**	Kebbi	Suru	207	30	6.9	12	6	20	2.4	0.23
**38**	Kebbi	Koko Besse	201	30	6.7	17	9	30	4.2	0.56
**39**	Kaduna	Sabo Gari	207	30	6.9	77	66	87	3.0	0.34
**40**	Kaduna	Kaduna South	186	30	6.4	87	78	92	1.9	0.16

1 Only clusters that were surveyed are included

2 When the design effect was estimated to be less than 1 we present it as 1, and the ICC=0.

Table 7: OPV3 Coverage per LGA,Nigeria 2014. Immunization Coverage Survey[1,2]										
**S/No**	**State**	**Lga**	**Sample Size**	**Cluster**	**Average No. of respondents/ cluster**	**Coverage Estimate (%)**	**Lower 95% CI**	**Upper 95% CI**	**Design Effect**	**ICC**
**1**	Sokoto	Illela	195	30	6.5	3	1	8	1.7	0.13
**2**	Sokoto	Wurno	210	30	7.0	18	11	29	2.8	0.30
**3**	Sokoto	Rabah	210	30	7.0	2	1	5	1.0	0.00
**4**	Sokoto	Kware	201	30	6.7	2	1	6	1.3	0.05
**5**	Sokoto	Shagari	206	30	6.9	1	0	6	1.6	0.11
**6**	Sokoto	Wamako	203	30	6.8	12	6	21	2.4	0.25
**7**	Katsina	Batagarawa	207	30	6.9	21	11	31	3.1	0.35
**8**	Katsina	Mashi	202	30	6.7	5	3	11	1.5	0.08
**9**	Katsina	Batsari	192	28	6.9	13	8	22	2.0	0.17
**10**	Katsina	Malumfashi	193	30	6.4	24	14	34	2.6	0.29
**11**	Katsina	Bakori	200	30	6.7	23	15	31	1.9	0.16
**12**	Katsina	Funtua	204	30	6.8	31	23	40	1.8	0.13
**13**	Jigawa	Kazaure	209	30	7.0	57	45	69	2.9	0.32
**14**	Jigawa	Babura	198	30	6.6	23	14	33	2.3	0.23
**15**	Jigawa	Maigatari	207	30	6.9	19	11	33	4.0	0.51
**16**	Jigawa	Miga	197	30	6.6	6	3	12	1.7	0.12
**17**	Jigawa	Kiyawa	180	30	6.0	18	11	27	1.9	0.18
**18**	Jigawa	Birnin_Kudu	177	29	6.1	32	22	41	1.8	0.15
**19**	FCT	Amac	192	29	6.6	66	58	73	1.2	0.04
**20**	Zamfara	Bukkuyum	194	29	6.7	12	6	23	3.1	0.36
**21**	Zamfara	Maradun	208	30	6.9	25	14	35	2.7	0.29
**22**	Zamfara	Shinkafi	208	30	6.9	30	22	38	1.4	0.07
**23**	Zamfara	Gusau	157	23	6.8	10	5	21	2.5	0.25
**24**	Zamfara	Bungudu	159	23	6.9	16	10	25	1.5	0.09
**25**	Zamfara	Maru	141	23	6.1	7	3	16	2.1	0.21
**26**	Bauchi	Gamawa	181	28	6.5	30	20	41	2.4	0.25
**27**	Bauchi	Itas-Gadau	208	30	6.9	44	31	56	3.2	0.37
**28**	Bauchi	Ningi	203	30	6.8	18	12	27	1.9	0.16
**29**	Bauchi	Bauchi	198	29	6.8	28	20	37	1.8	0.13
**30**	Kaduna	Makarfi	203	30	6.8	20	11	30	2.8	0.31
**31**	Kaduna	Ikara	207	30	6.9	21	9	32	3.9	0.50
**32**	Kaduna	Zaria	205	30	6.8	27	18	35	1.8	0.14
**33**	Kaduna	Igabi	202	29	7.0	29	18	40	2.8	0.30
**34**	Kaduna	Kaduna North	186	29	6.4	32	24	41	1.4	0.07
**35**	Kaduna	Chikun	171	25	6.8	56	47	66	1.5	0.08
**36**	Kebbi	Jega	200	30	6.7	8	4	15	1.8	0.14
**37**	Kebbi	Suru	207	30	6.9	6	3	13	2.1	0.19
**38**	Kebbi	Koko Besse	201	30	6.7	3	1	7	1.2	0.03
**39**	Kaduna	Sabo Gari	207	30	6.9	50	41	60	1.8	0.14
**40**	Kaduna	Kaduna South	186	30	6.4	50	43	57	1.0	0.00

1 Only clusters that were surveyed are included

2 When the design effect was estimated to be less than 1 we present it as 1, and the ICC=0.

Table 8: Complete Coverage per LGA, Nigeria 2014. Immunization Coverage Survey[1,2]										
S/No	State	Lga	Sample Size	Cluster	Average No. of respondents/ cluster	Coverage Estimate (%)	Lower 95% CI	Upper 95% CI	Design Effect	ICC
1	Sokoto	Illela	195	30	6.5	1	0	3	1.0	0.00
2	Sokoto	Wurno	210	30	7.0	10	5	16	1.6	0.11
3	Sokoto	Rabah	210	30	7.0	0	0	3	1.0	0.00
4	Sokoto	Kware	201	30	6.7	0	0	0	1.0	0.00
5	Sokoto	Shagari	206	30	6.9	0	0	3	1.0	0.00
6	Sokoto	Wamako	203	30	6.8	7	3	15	2.8	0.30
7	Katsina	Batagarawa	207	30	6.9	10	5	18	2.5	0.26
8	Katsina	Mashi	202	30	6.7	1	0	4	1.0	0.00
9	Katsina	Batsari	192	28	6.9	8	4	18	2.7	0.29
10	Katsina	Malumfashi	193	30	6.4	8	4	16	2.2	0.21
11	Katsina	Bakori	200	30	6.7	14	8	21	1.7	0.13
12	Katsina	Funtua	204	30	6.8	19	13	27	1.7	0.11
13	Jigawa	Kazaure	209	30	7.0	40	29	51	2.6	0.26
14	Jigawa	Babura	198	30	6.6	6	3	11	1.1	0.02
15	Jigawa	Maigatari	207	30	6.9	6	2	13	2.5	0.25
16	Jigawa	Miga	197	30	6.6	1	0	4	1.0	0.00
17	Jigawa	Kiyawa	180	30	6.0	13	8	21	1.6	0.13
18	Jigawa	Birnin_Kudu	177	29	6.1	15	9	26	2.4	0.27
19	FCT	Amac	192	29	6.6	51	42	60	1.4	0.06
20	Zamfara	Bukkuyum	194	29	6.7	10	5	20	2.9	0.33
21	Zamfara	Maradun	208	30	6.9	16	8	28	3.7	0.46
22	Zamfara	Shinkafi	208	30	6.9	20	15	26	1.1	0.01
23	Zamfara	Gusau	157	23	6.8	4	1	16	3.8	0.49
24	Zamfara	Bungudu	159	23	6.9	8	4	13	1.1	0.01
25	Zamfara	Maru	141	23	6.1	3	1	8	1.4	0.07
26	Bauchi	Gamawa	181	28	6.5	21	10	32	3.4	0.43
27	Bauchi	Itas-Gadau	208	30	6.9	36	24	48	3.1	0.35
28	Bauchi	Ningi	203	30	6.8	11	7	19	1.7	0.13
29	Bauchi	Bauchi	198	29	6.8	17	12	25	1.5	0.08
30	Kaduna	Makarfi	203	30	6.8	11	6	18	1.9	0.15
31	Kaduna	Ikara	207	30	6.9	15	8	28	4.0	0.52
32	Kaduna	Zaria	205	30	6.8	5	3	9	1.0	0.00
33	Kaduna	Igabi	202	29	7.0	18	11	27	2.2	0.20
34	Kaduna	Kaduna North	186	29	6.4	24	16	31	1.3	0.06
35	Kaduna	Chikun	171	25	6.8	47	38	55	1.2	0.03
36	Kebbi	Jega	200	30	6.7	3	1	7	1.2	0.03
37	Kebbi	Suru	207	30	6.9	2	1	8	2.1	0.19
38	Kebbi	Koko Besse	201	30	6.7	1	1	4	1.0	0.00
39	Kaduna	Sabo Gari	207	30	6.9	33	23	43	2.4	0.23
40	Kaduna	Kaduna South	186	30	6.4	29	23	35	1.0	0.00

1 Only clusters that were surveyed are included

2 When the design effect was estimated to be less than 1 we present it as 1, and the ICC=0.
