# Supplementary material for: Routine Vaccination Coverage in Northern Nigeria: Results from 40 District-Level Cluster Surveys, 2014-2015
Source: PLoS One. 2016 Dec 9;11(12):e0167835. doi: 10.1371/journal.pone.0167835 (PMC5148043; doi:10.1371/journal.pone.0167835)
Supplement: S3 Appendix — The order of the LGAs within a state is based on DPT3 coverage. LGAs are grouped by state to illustrate variability in coverage across LGAs within the same state. This data is not representative of state-level coverage since LGAs were purposefully selected. Graphs of routine immunization coverage estimates, grouped by state. All coverage estimates combine maternal recall + vaccine card data; complete coverage = 8 antigens (BCG, OPV 1, DPT 1, OPV 2, DPT 2, OPV 3, DPT 3, Measles); does not include OPV or measles doses from SIAs. (PDF) [file pone.0167835.s003.pdf]

Supplementary Appendix 3

Routine immunization coverage estimates for each LGA, grouped by antigen

NB: All coverage estimates combine maternal recall + vaccine card data; complete coverage = 8 antigens (BCG, OPV 1, DPT 1, OPV 2, DPT 2, OPV 3, DPT 3, Measles); does not include OPV doses from SIAs.

The order of the LGAs within a state is based on DPT3 coverage. LGAs are grouped by state to illustrate variability in coverage across LGAs within the same state. This data is not representative of state-level coverage since LGAs were purposefully selected.

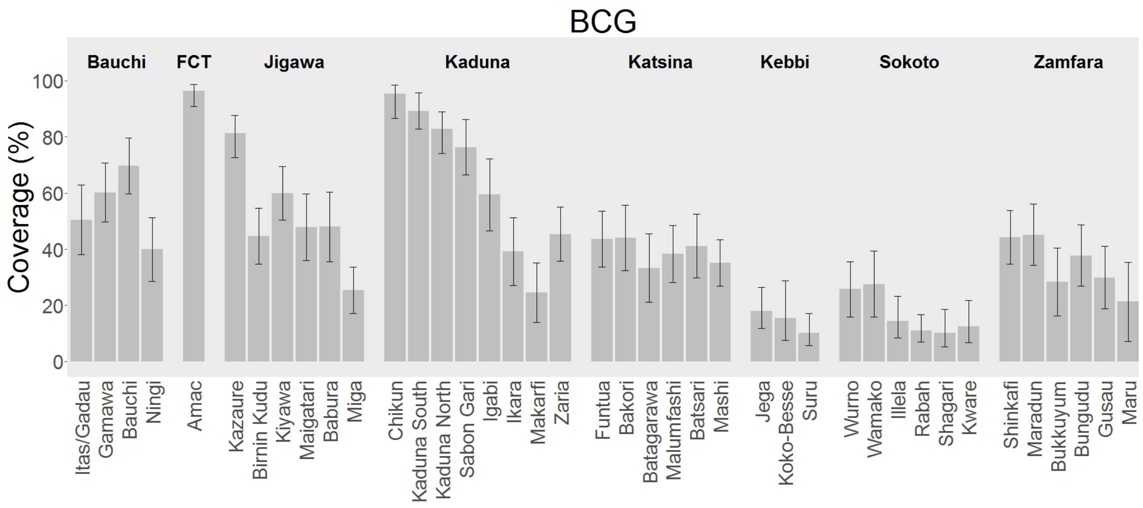

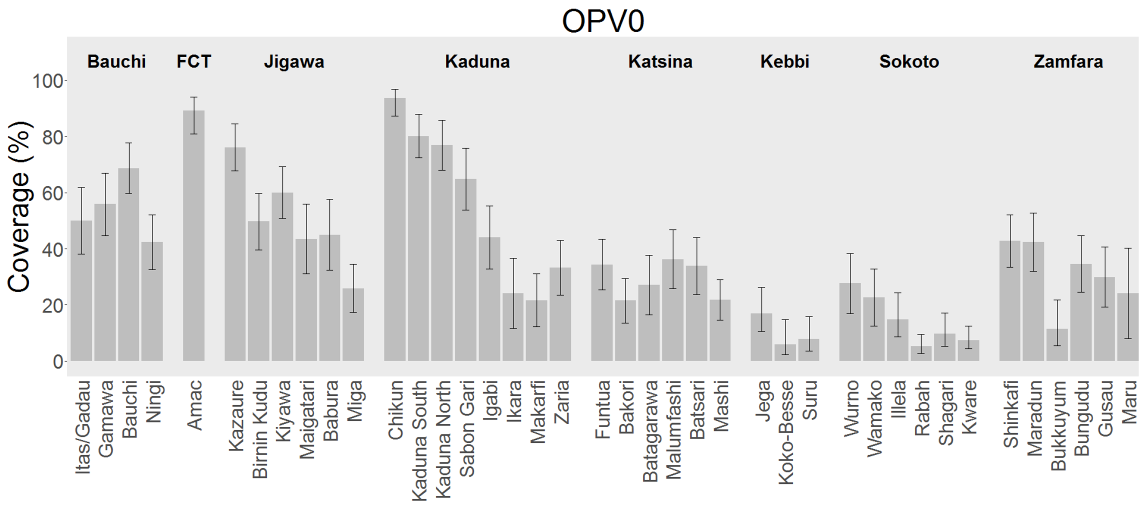

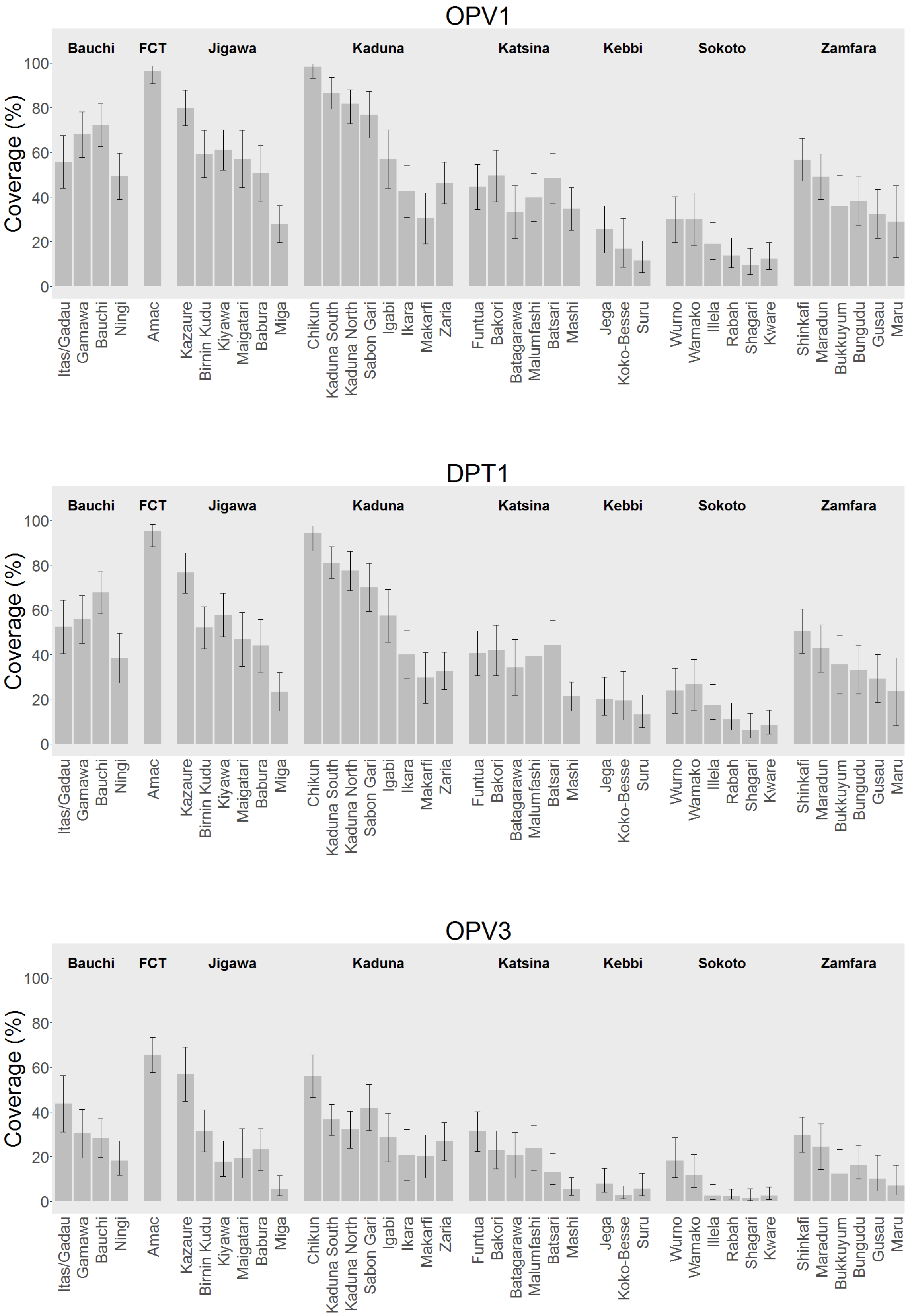
OPV1
DPT1
OPV3
Coverage (%)
0
20
40
60
80
100
Bauchi
FCT
Jigawa
Kaduna
Katsina
Kebbi
Sokoto
Zamfara
Itas/Gadau
Gamawa
Bauchi
Ningi
Amac
Kazaure
Birnin Kudu
Kiyawa
Maigatari
Babura
Miga
Chikun
Kaduna South
Kaduna North
Sabon Gari
Igabi
Ikara
Makarfi
Zaria
Funtua
Bakori
Batagarawa
Malumfashi
Batsari
Mashi
Jega
Koko-Besse
Suru
Wurno
Wamako
Illela
Rabah
Shagari
Kware
Shinkafi
Maradun
Bukkuyum
Bungudu
Gusau
Maru

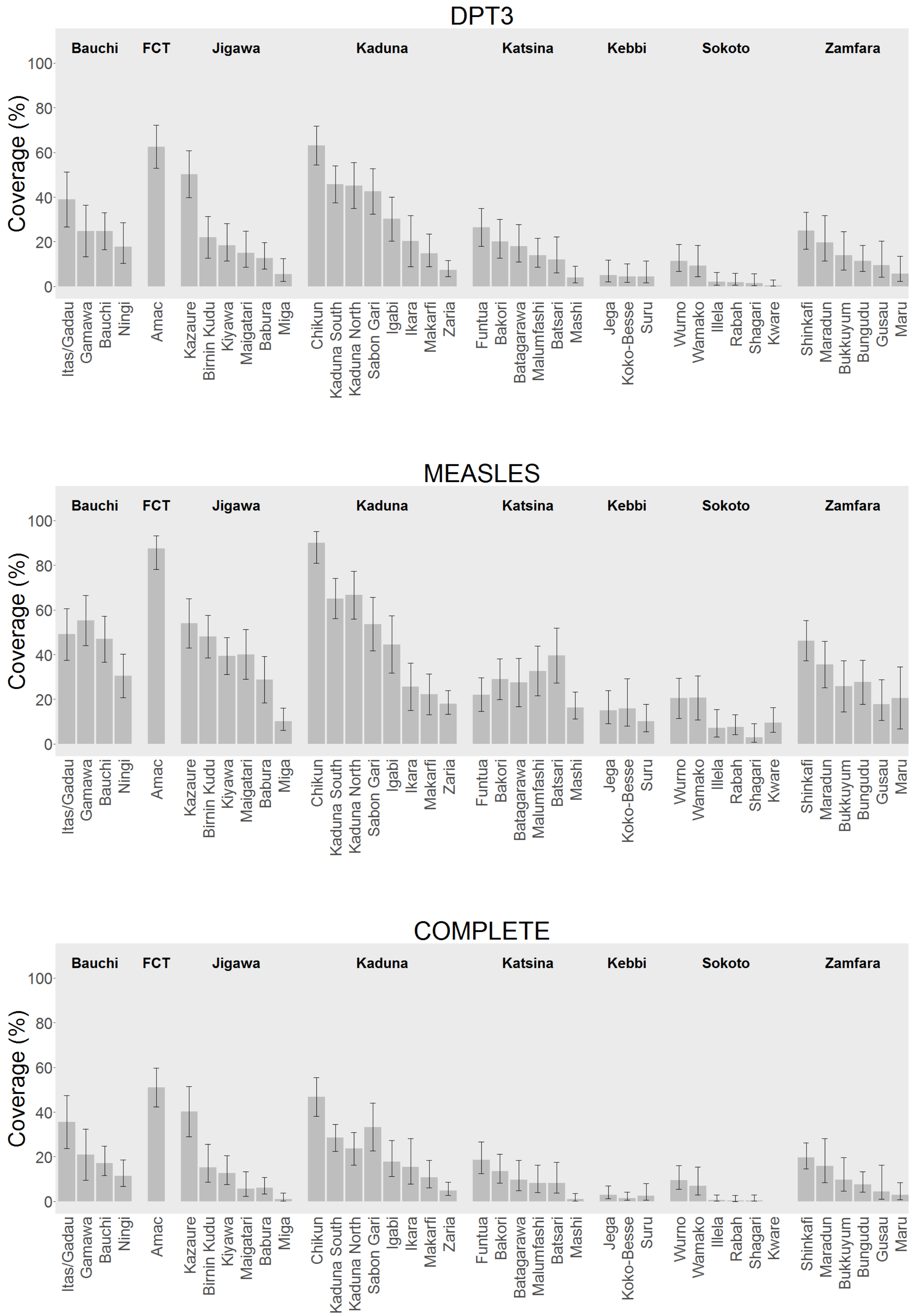

DPT3
MEASLES
COMPLETE
Coverage (%)
Bauchi
FCT
Jigawa
Kaduna
Katsina
Kebbi
Sokoto
Zamfara
Itas/Gadau
Gamawa
Bauchi
Ningi
Amac
Kazaure
Birnin Kudu
Kiyawa
Maigatari
Babura
Miga
Chikun
Kaduna South
Kaduna North
Sabon Gari
Igabi
Ikara
Makarfi
Zaria
Funtua
Bakori
Batagarawa
Malumfashi
Batsari
Mashi
Jega
Koko-Besse
Suru
Wurno
Wamako
Illela
Rabah
Shagari
Kware
Shinkafi
Maradun
Bukkuyum
Bungudu
Gusau
Maru
0
20
40
60
80
100

Routine immunization coverage estimates, grouped by state

NB: All coverage estimates combine maternal recall + vaccine card data; complete coverage = 8 antigens (BCG, OPV 1, DPT 1, OPV 2, DPT 2, OPV 3, DPT 3, Measles); does not include OPV doses from SIAs.

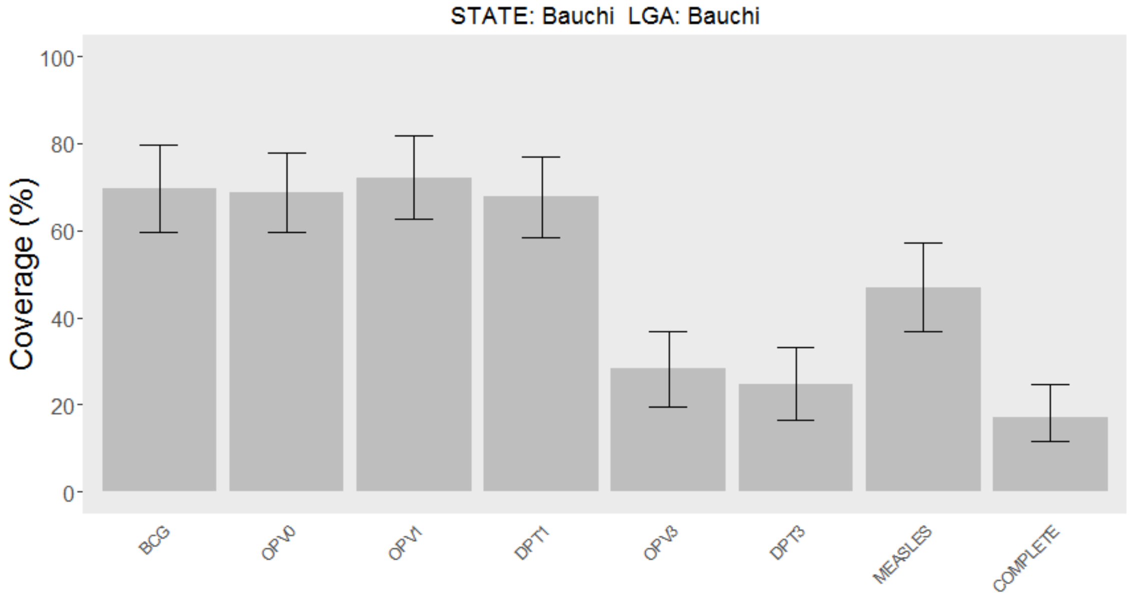

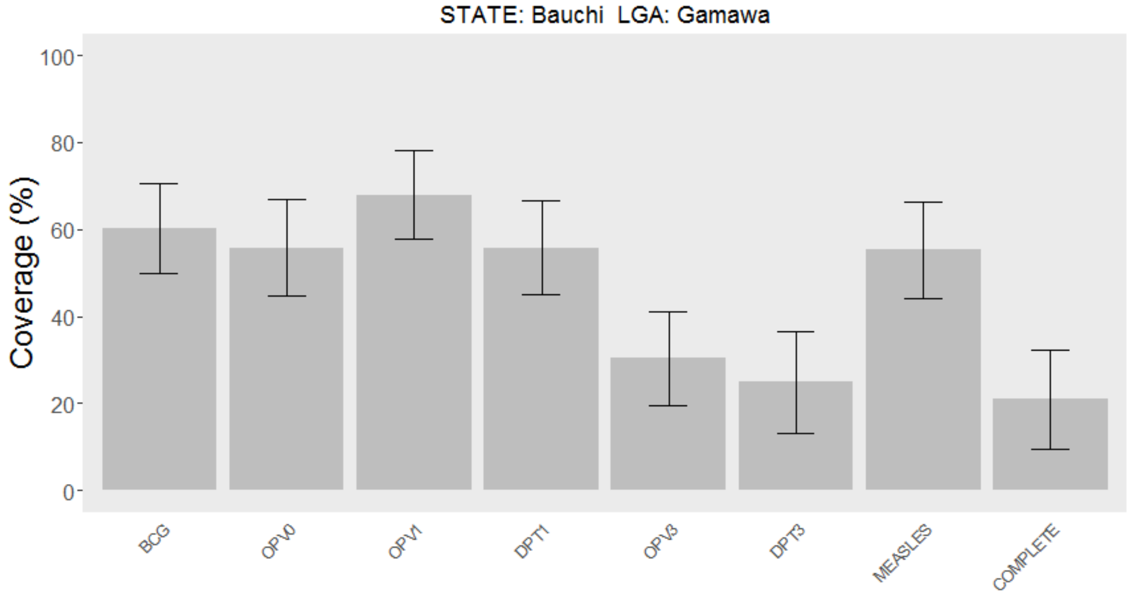

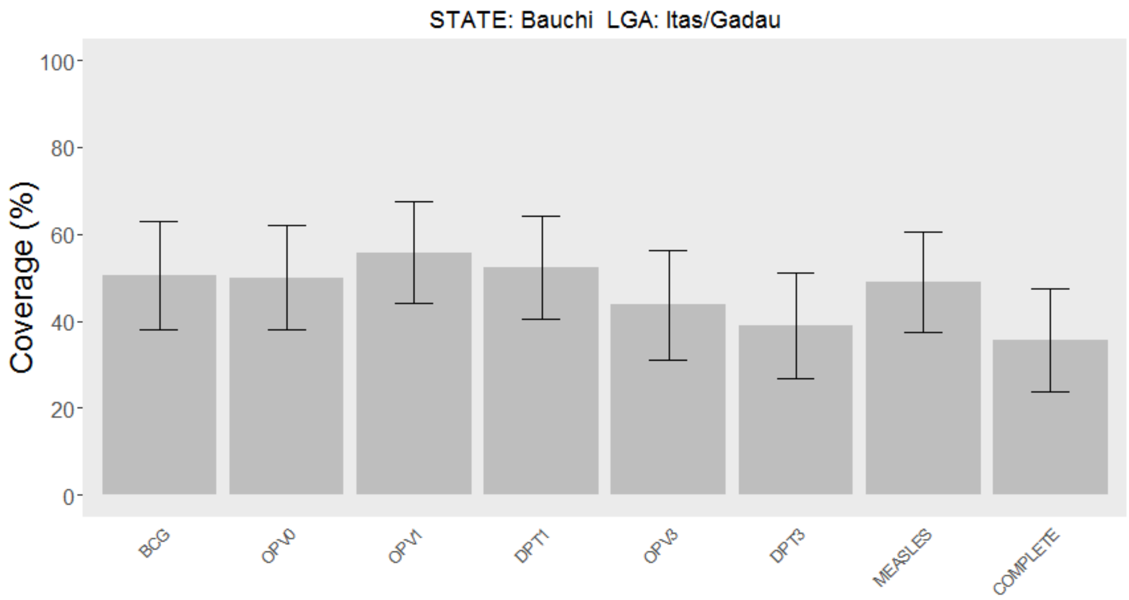
STATE: Bauchi LGA: Itas/Gadau
Coverage (%)
100
80
60
40
20
0
BCG
OPV0
OPV1
DPT1
OPV3
DPT3
MEASLES
COMPLETE

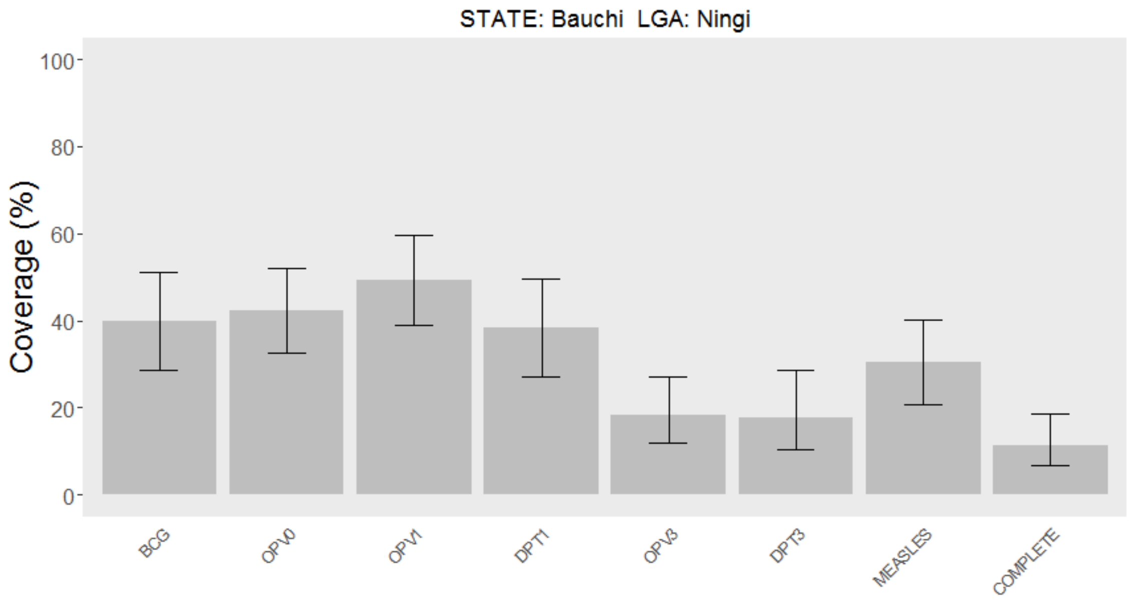
STATE: Bauchi LGA: Ningi
Coverage (%)
100
80
60
40
20
0
BCG
OPV0
OPV1
DPT1
OPV3
DPT3
MEASLES
COMPLETE

STATE: FCT LGA: Amac

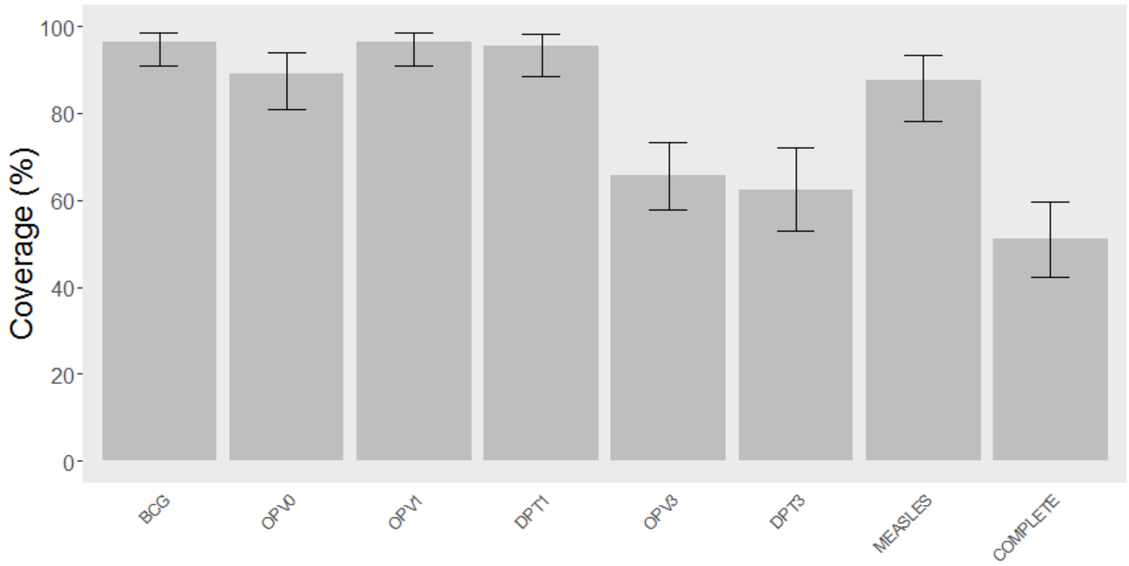

STATE: Jigawa LGA: Babura

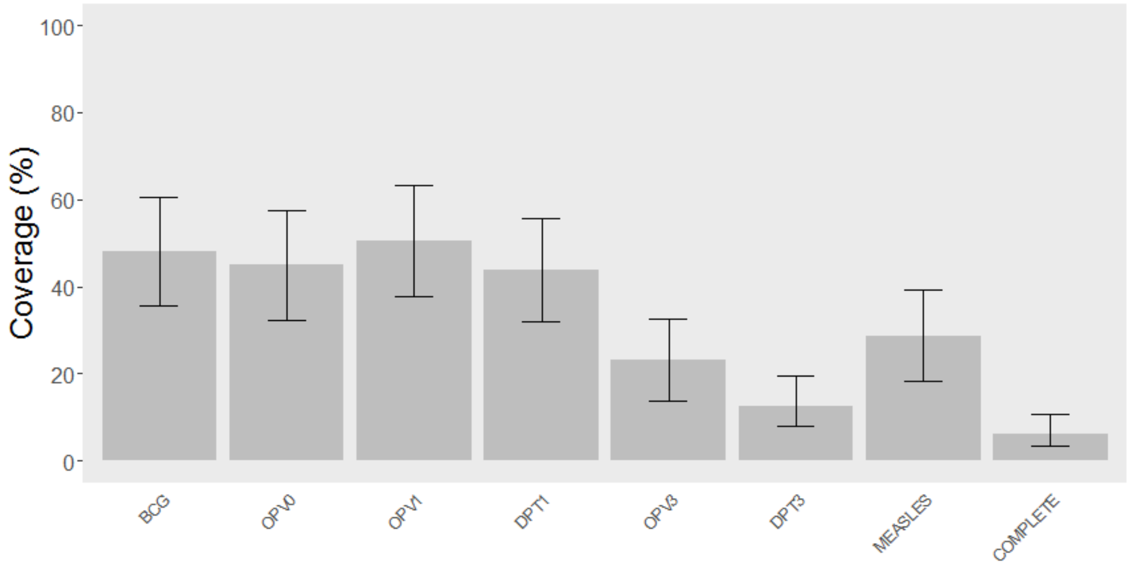

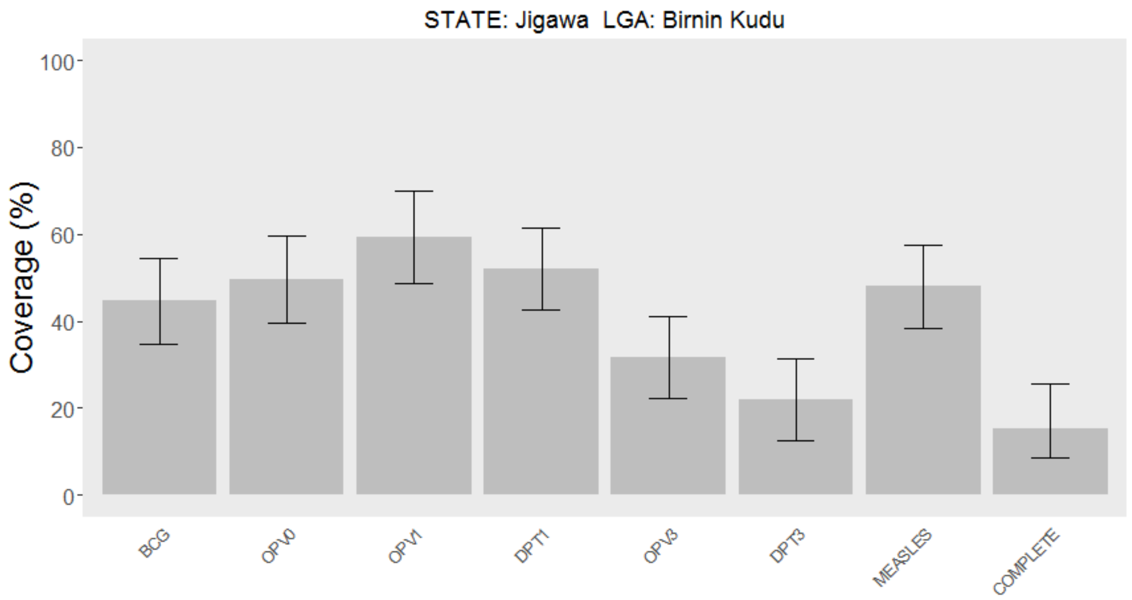
STATE: Jigawa LGA: Birnin Kudu
Coverage (%)
100
80
60
40
20
0
BCG
OPV0
OPV1
DPT1
OPV3
DPT3
MEASLES
COMPLETE

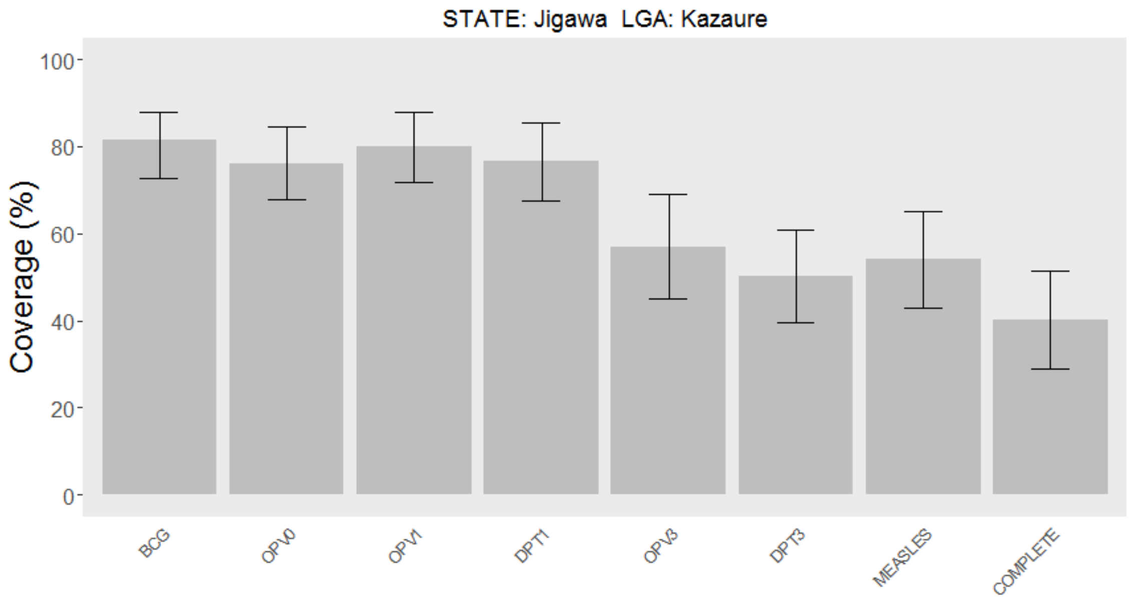
STATE: Jigawa LGA: Kazaure
Coverage (%)
100
80
60
40
20
0
BCG
OPV0
OPV1
DPT1
OPV3
DPT3
MEASLES
COMPLETE

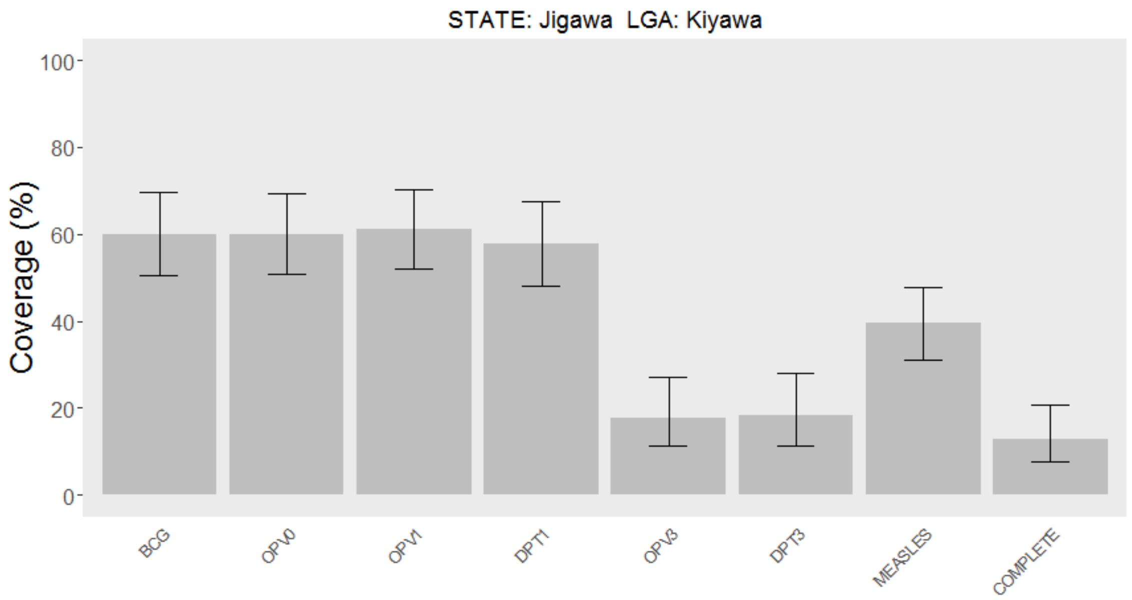
STATE: Jigawa LGA: Kiyawa
Coverage (%)
100
80
60
40
20
0
BCG
OPV0
OPV1
DPT1
OPV3
DPT3
MEASLES
COMPLETE

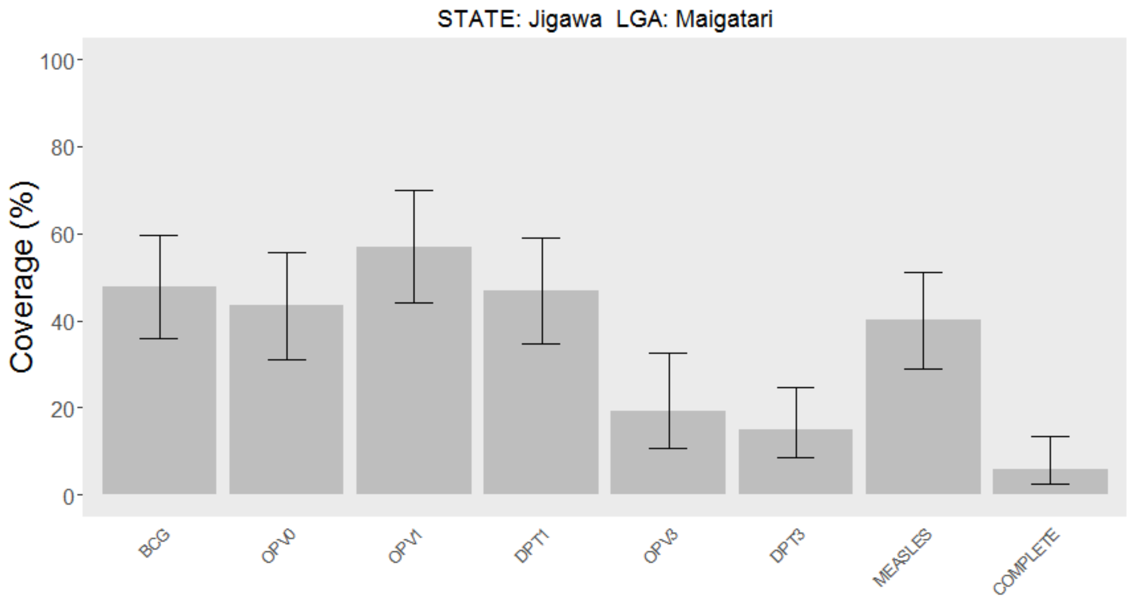
STATE: Jigawa LGA: Maigatari
Coverage (%)
100
80
60
40
20
0
BCG
OPV0
OPV1
DPT1
OPV3
DPT3
MEASLES
COMPLETE

STATE: Jigawa LGA: Miga

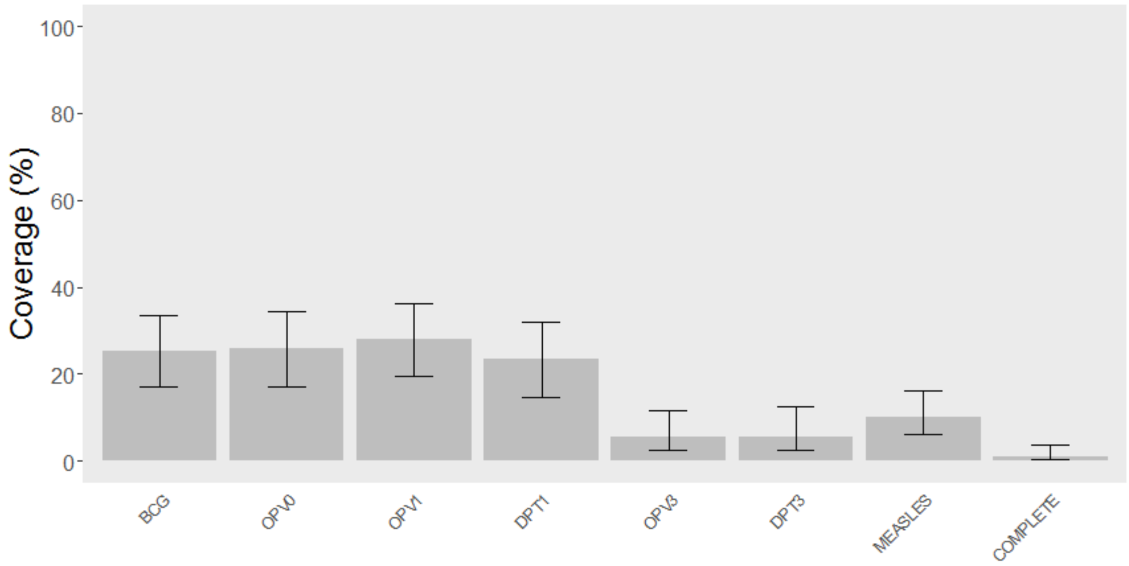

STATE: Kaduna LGA: Chikun

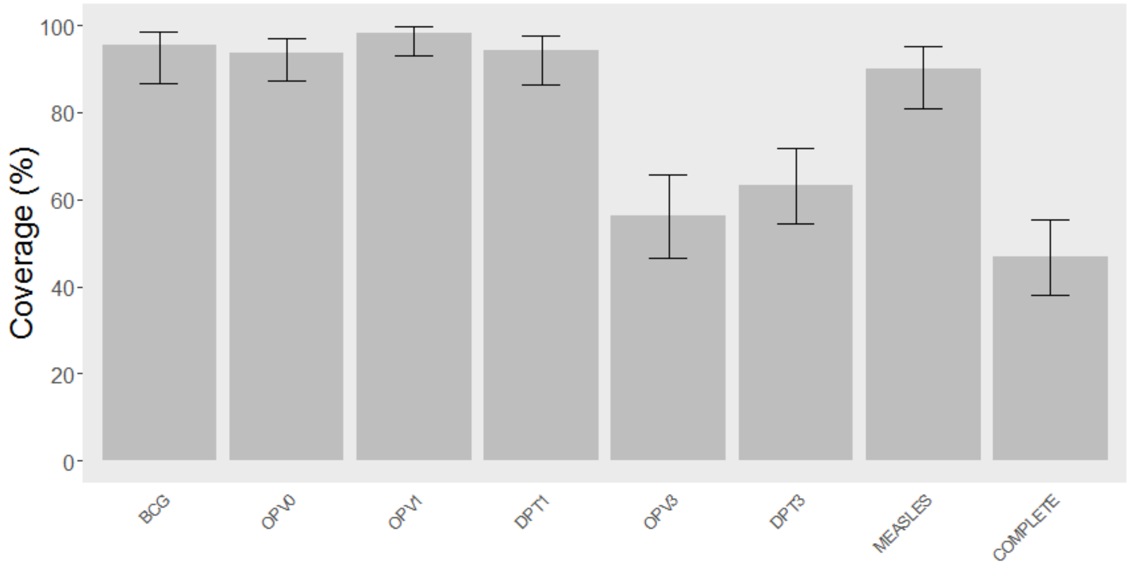

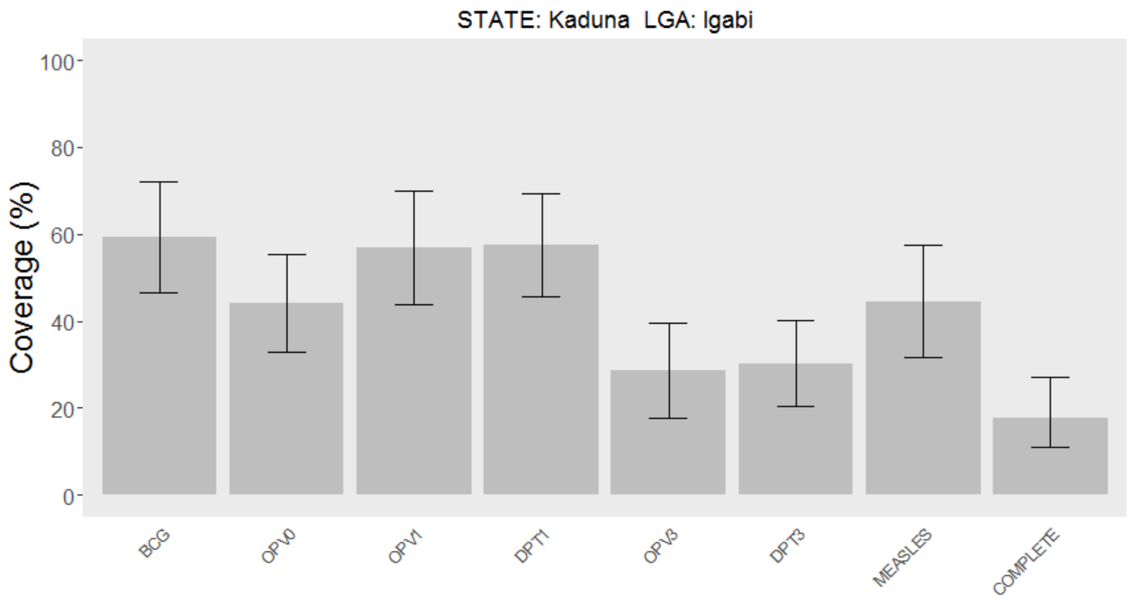
STATE: Kaduna LGA: Igabi
Coverage (%)
100
80
60
40
20
0
BCG
OPV0
OPV1
DPT1
OPV3
DPT3
MEASLES
COMPLETE

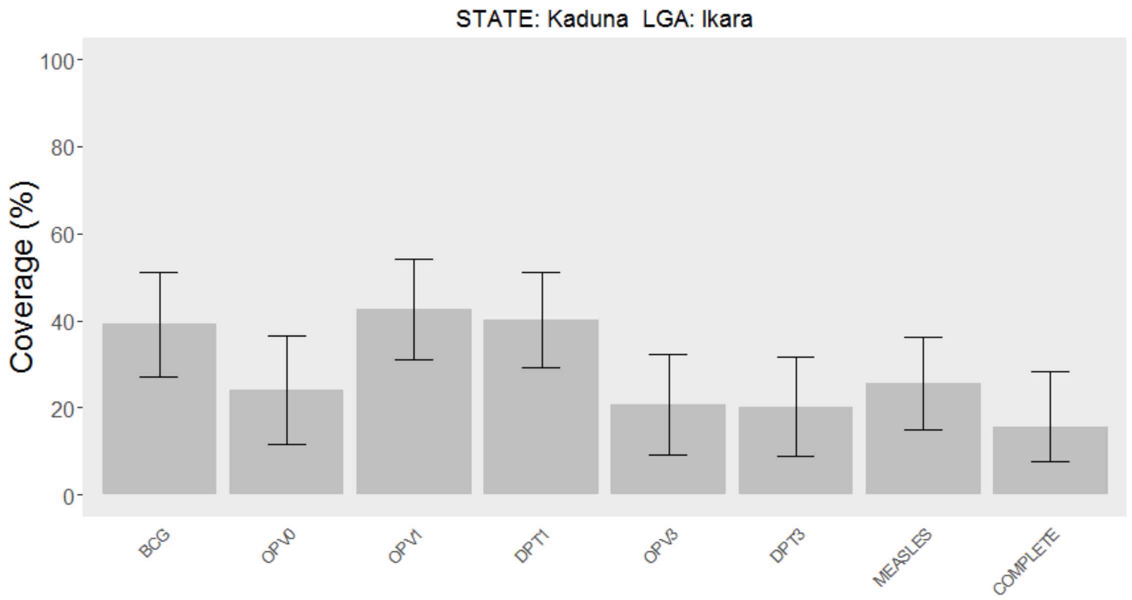
STATE: Kaduna LGA: Ikara
Coverage (%)
100
80
60
40
20
0
BCG
OPV0
OPV1
DPT1
OPV3
DPT3
MEASLES
COMPLETE

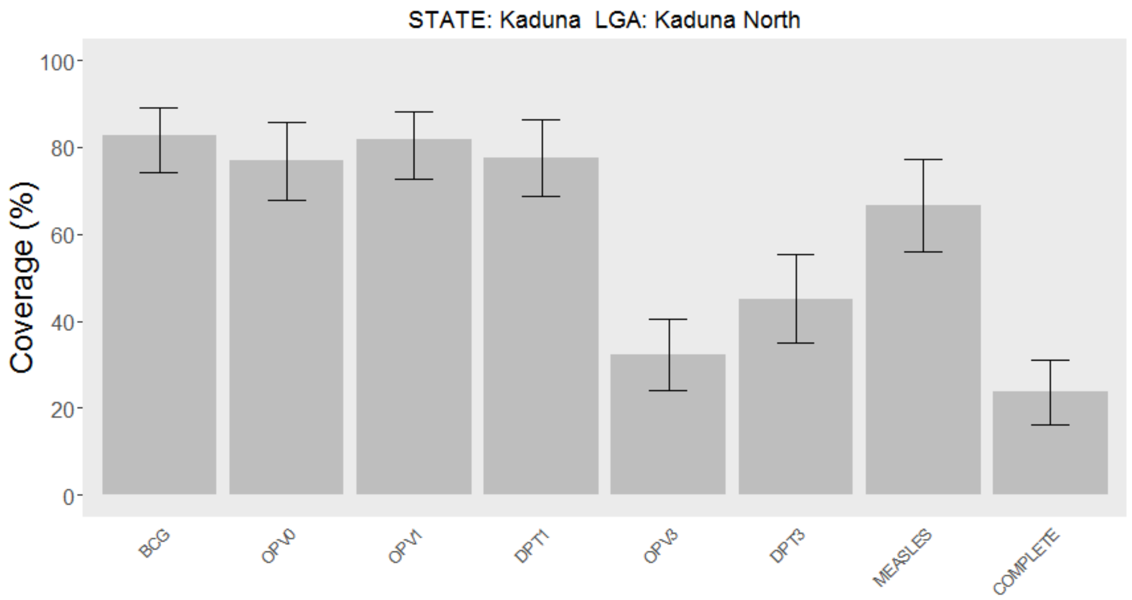
STATE: Kaduna LGA: Kaduna North
Coverage (%)
0
20
40
60
80
100
BCG
OPV0
OPV1
DPT1
OPV3
DPT3
MEASLES
COMPLETE

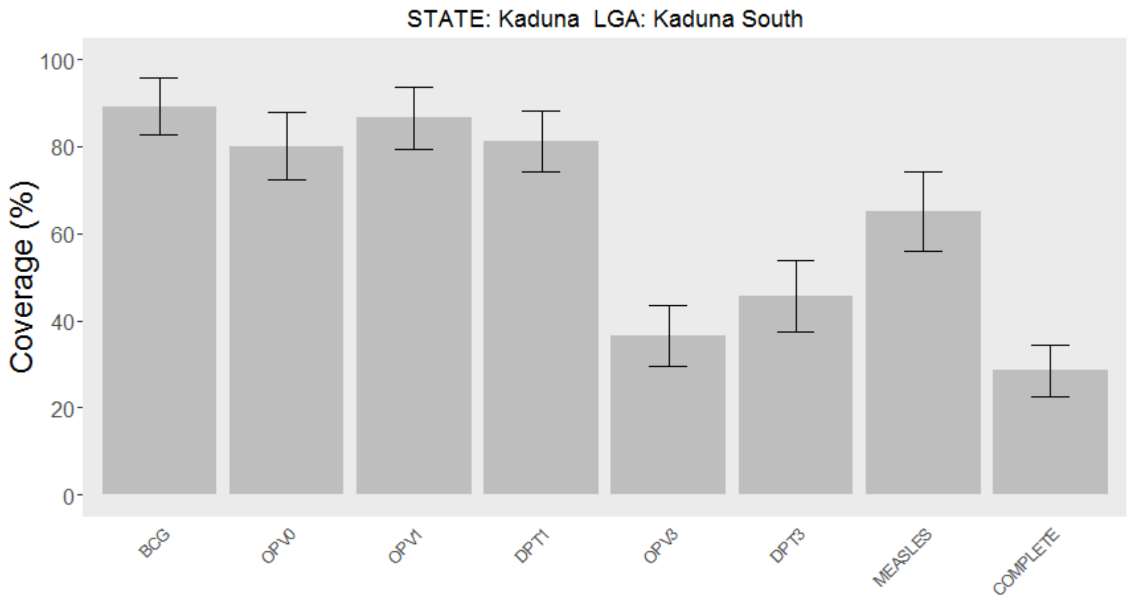
STATE: Kaduna LGA: Kaduna South
Coverage (%)
0
20
40
60
80
100
BCG
OPV0
OPV1
DPT1
OPV3
DPT3
MEASLES
COMPLETE

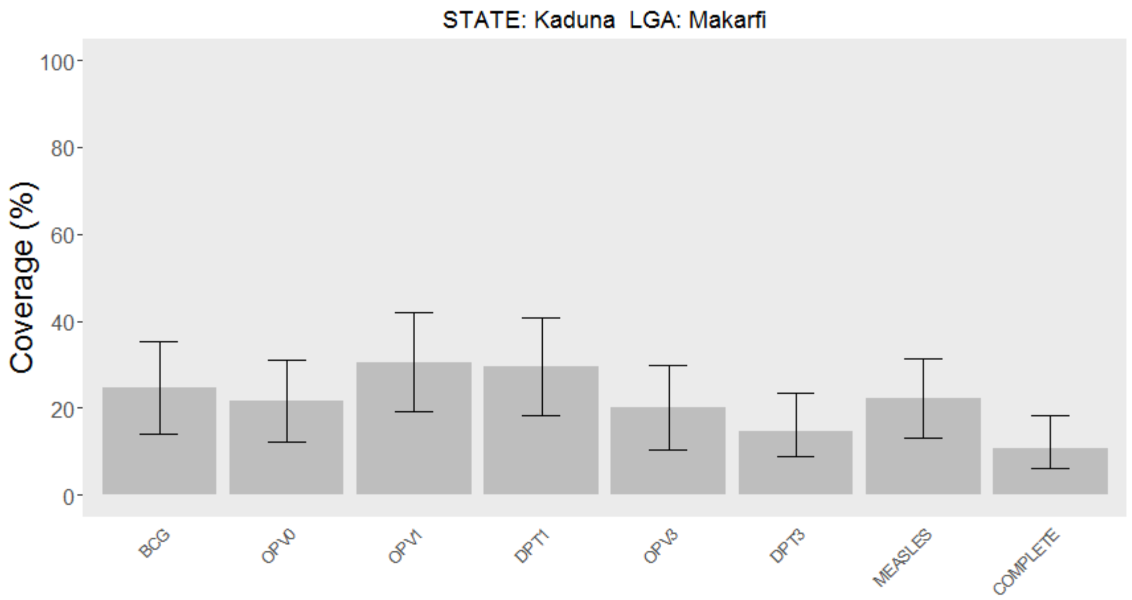
STATE: Kaduna LGA: Makarfi
Coverage (%)
0
20
40
60
80
100
BCG
OPV0
OPV1
DPT1
OPV3
DPT3
MEASLES
COMPLETE

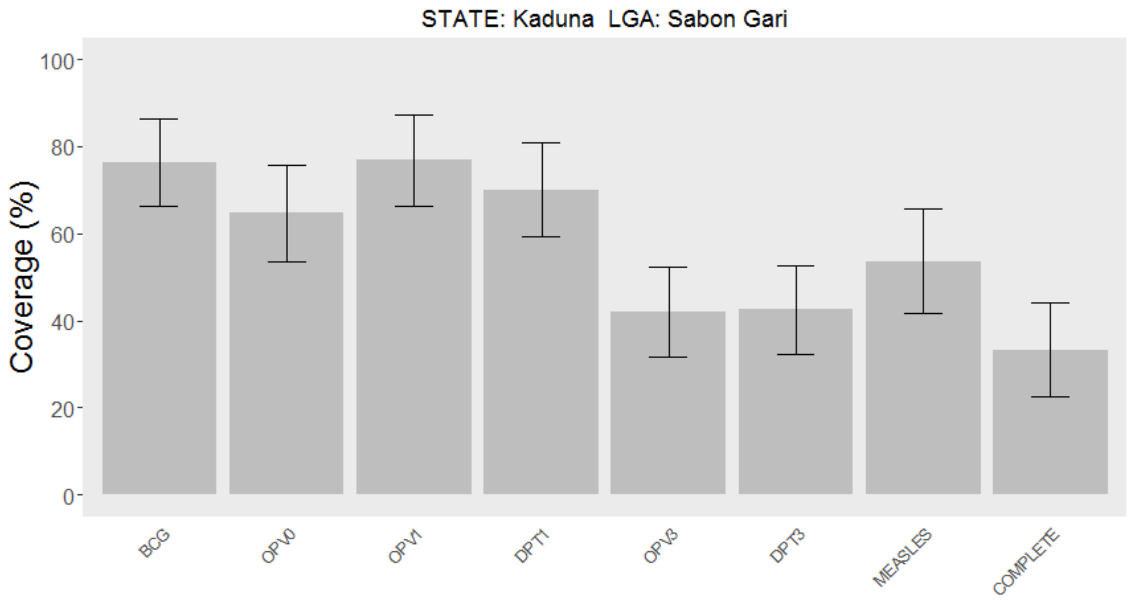
STATE: Kaduna LGA: Sabon Gari
Coverage (%)
0
20
40
60
80
100
BCG
OPV0
OPV1
DPT1
OPV3
DPT3
MEASLES
COMPLETE

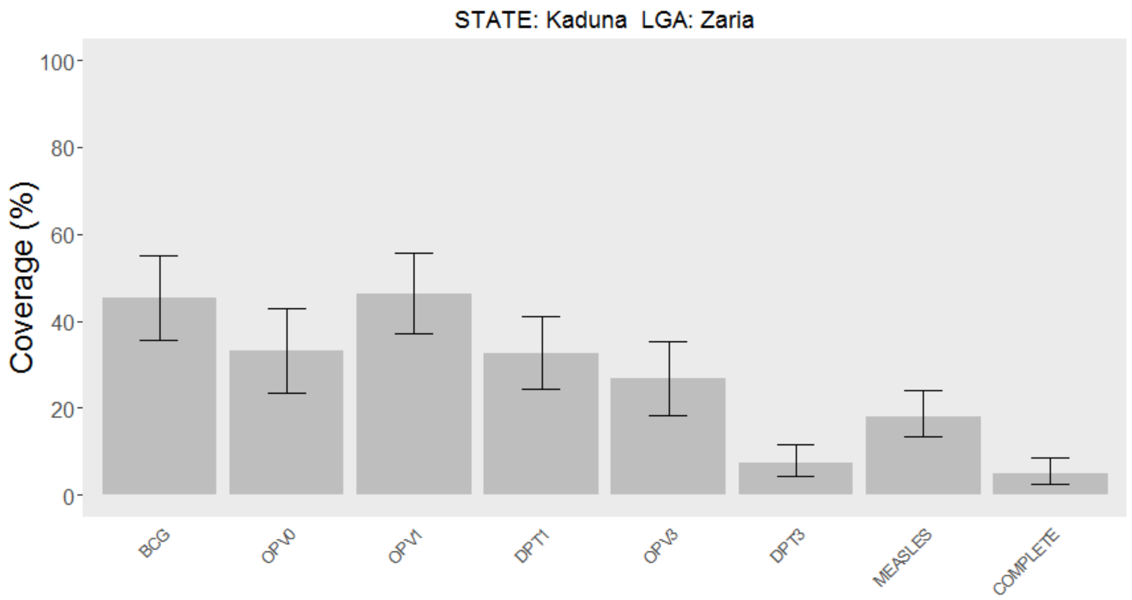
STATE: Kaduna LGA: Zaria
Coverage (%)
100
80
60
40
20
0
BCG
OPV0
OPV1
DPT1
OPV3
DPT3
MEASLES
COMPLETE

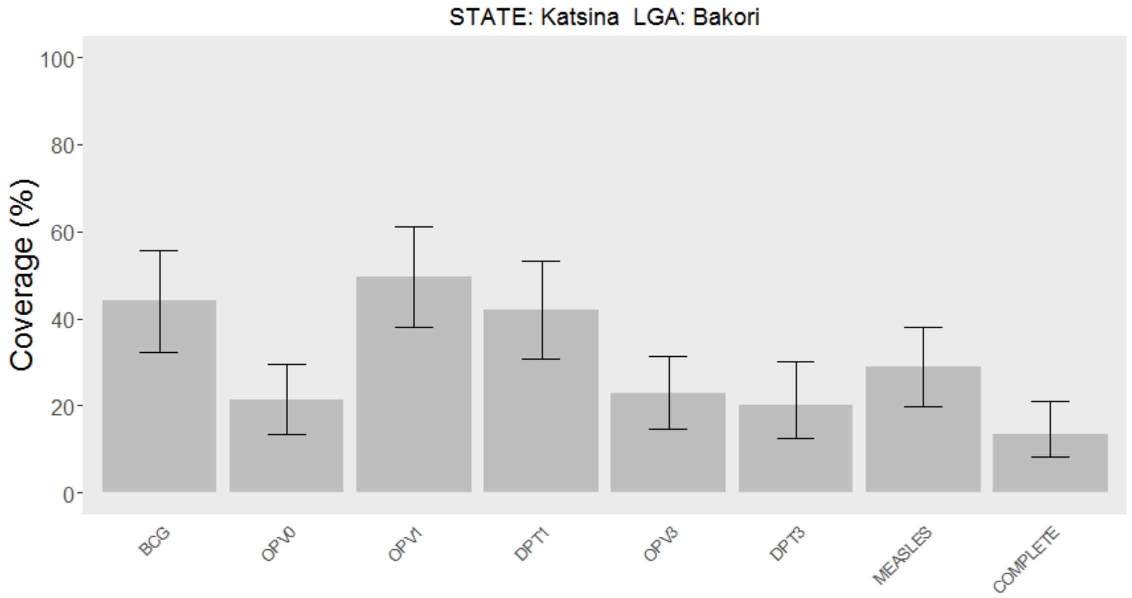
STATE: Katsina LGA: Bakori
Coverage (%)
100
80
60
40
20
0
BCG
OPV0
OPV1
DPT1
OPV3
DPT3
MEASLES
COMPLETE

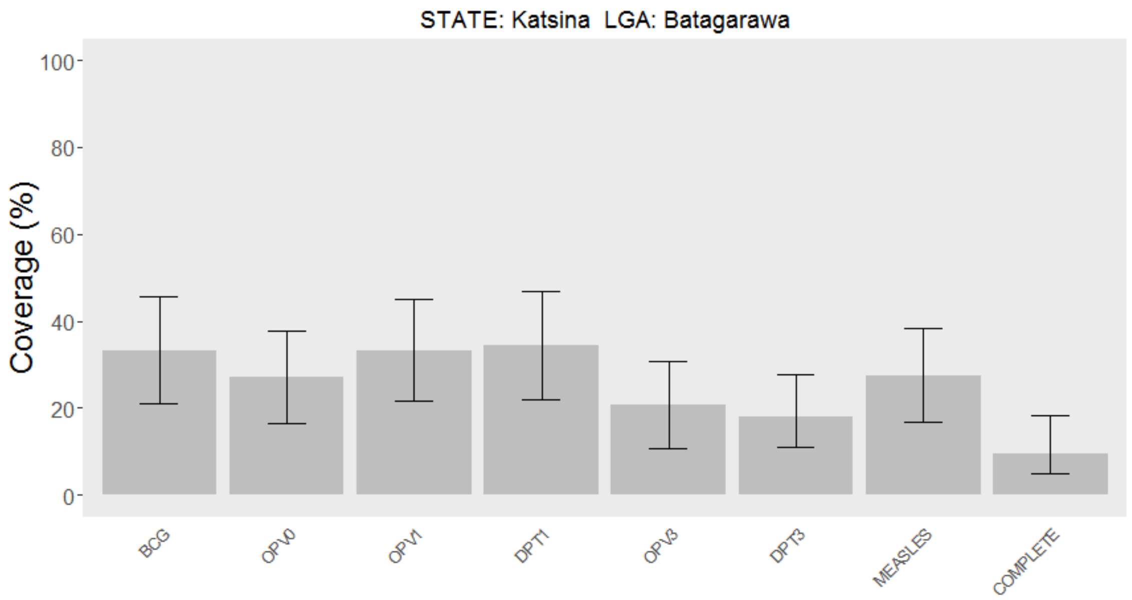
STATE: Katsina LGA: Batagarawa
Coverage (%)
100
80
60
40
20
0
BCG
OPV0
OPV1
DPT1
OPV3
DPT3
MEASLES
COMPLETE

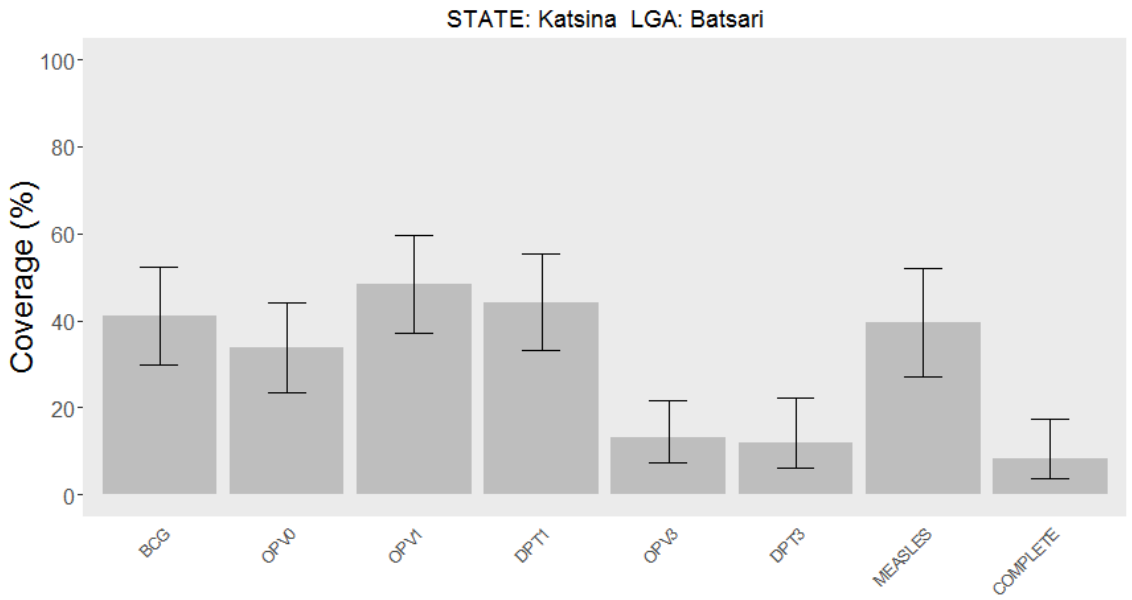
STATE: Katsina LGA: Batsari
Coverage (%)
100
80
60
40
20
0
BCG
OPV0
OPV1
DPT1
OPV3
DPT3
MEASLES
COMPLETE

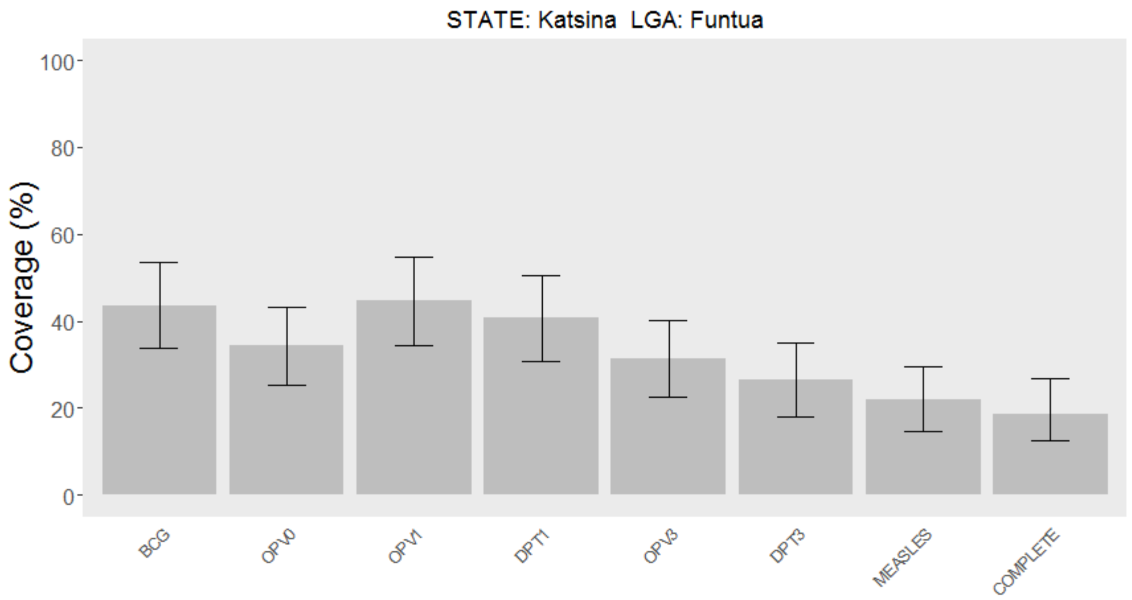
STATE: Katsina LGA: Funtua
Coverage (%)
0
20
40
60
80
100
BCG
OPV0
OPV1
DPT1
OPV3
DPT3
MEASLES
COMPLETE

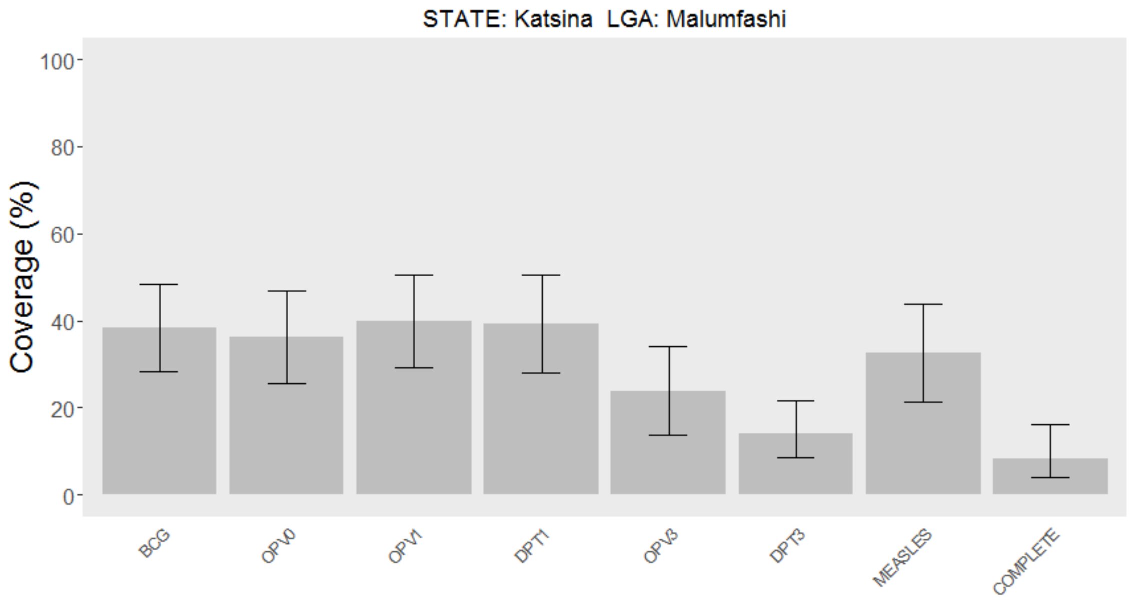
STATE: Katsina LGA: Malumfashi
Coverage (%)
0
20
40
60
80
100
BCG
OPV0
OPV1
DPT1
OPV3
DPT3
MEASLES
COMPLETE

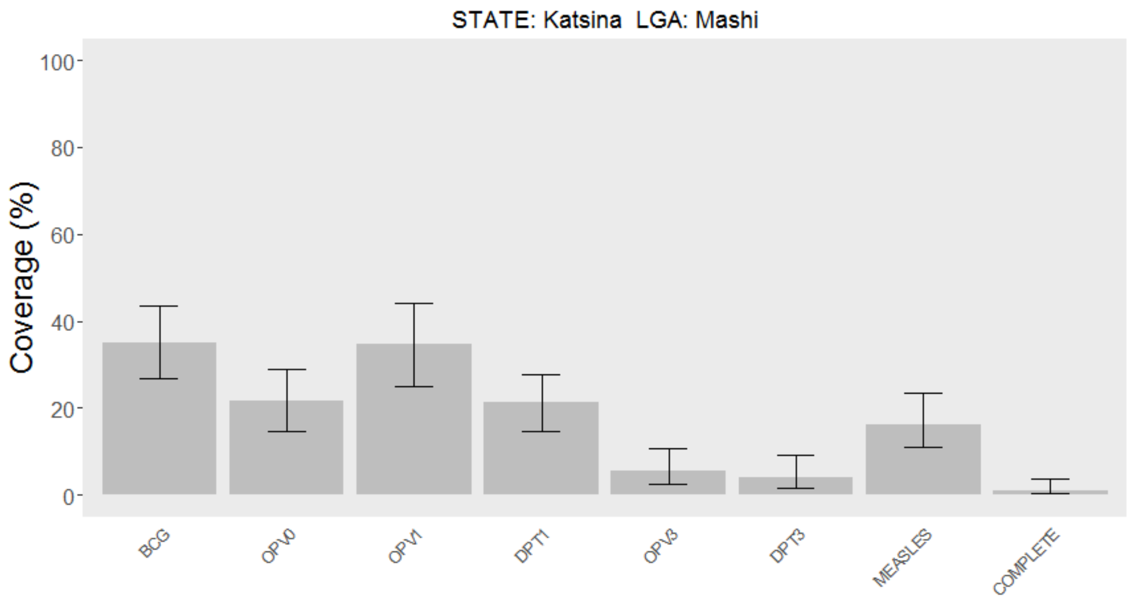
STATE: Katsina LGA: Mashi
Coverage (%)
100
80
60
40
20
0
BCG
OPV0
OPV1
DPT1
OPV3
DPT3
MEASLES
COMPLETE

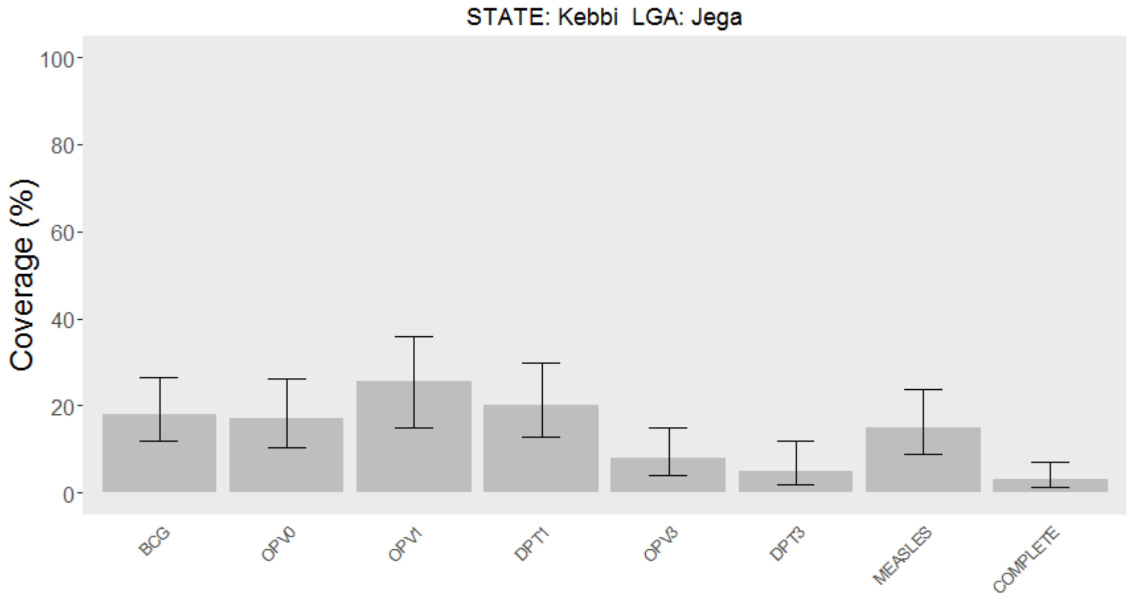
STATE: Kebbi LGA: Jega
Coverage (%)
100
80
60
40
20
0
BCG
OPV0
OPV1
DPT1
OPV3
DPT3
MEASLES
COMPLETE

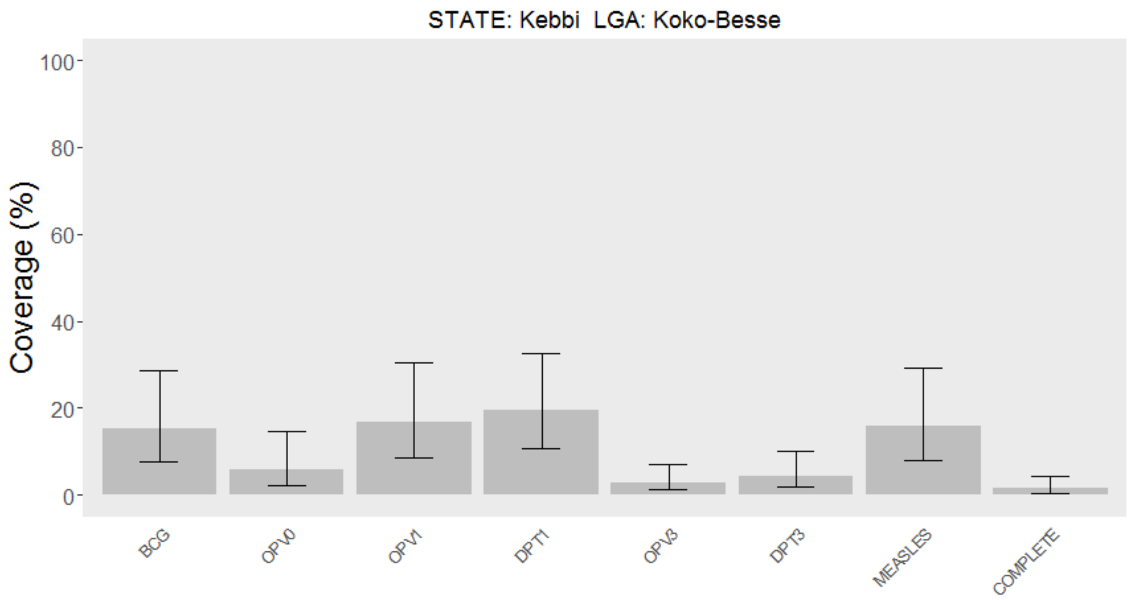

STATE: Kebbi LGA: Koko-Besse
Coverage (%)
100
80
60
40
20
0
BCG
OPV0
OPV1
DPT1
OPV3
DPT3
MEASLES
COMPLETE

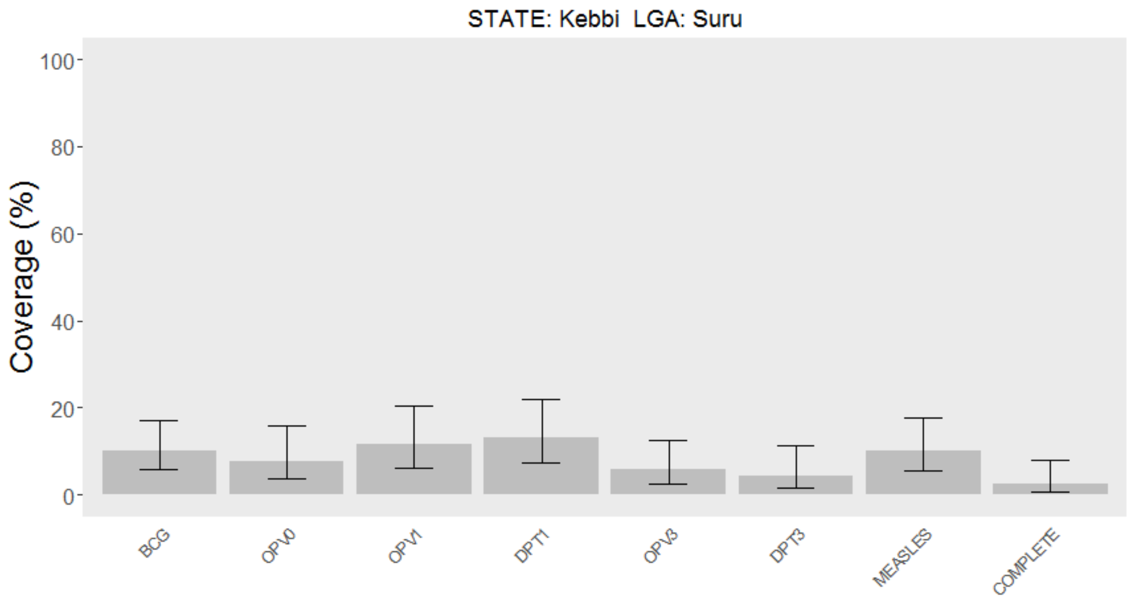

STATE: Kebbi LGA: Suru
Coverage (%)
100
80
60
40
20
0
BCG
OPV0
OPV1
DPT1
OPV3
DPT3
MEASLES
COMPLETE

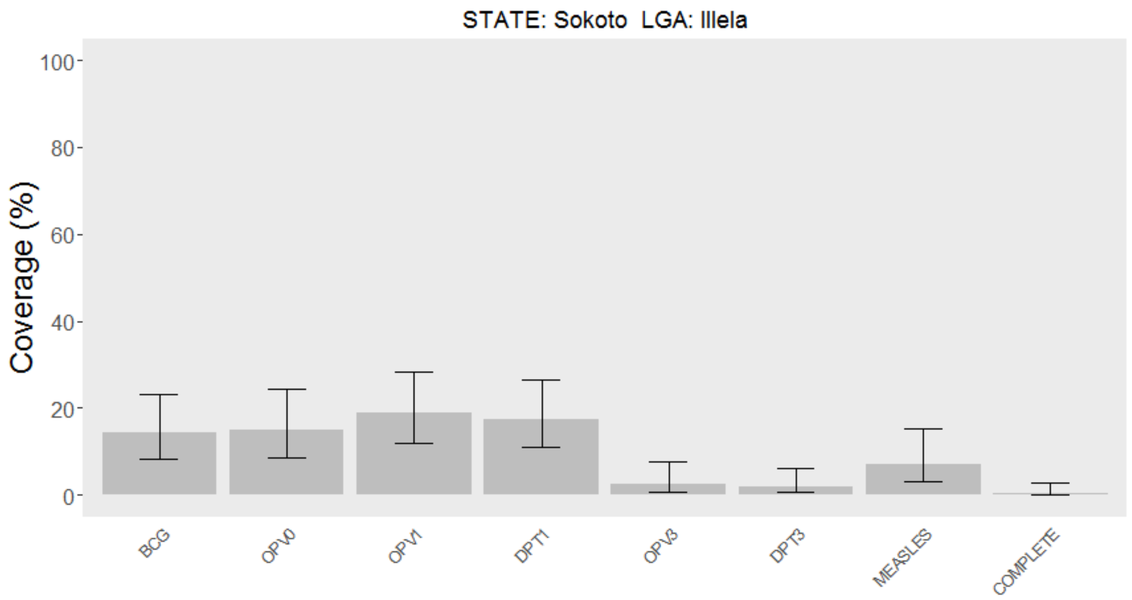
STATE: Sokoto LGA: Illela
Coverage (%)
0
20
40
60
80
100
BCG
OPV0
OPV1
DPT1
OPV3
DPT3
MEASLES
COMPLETE

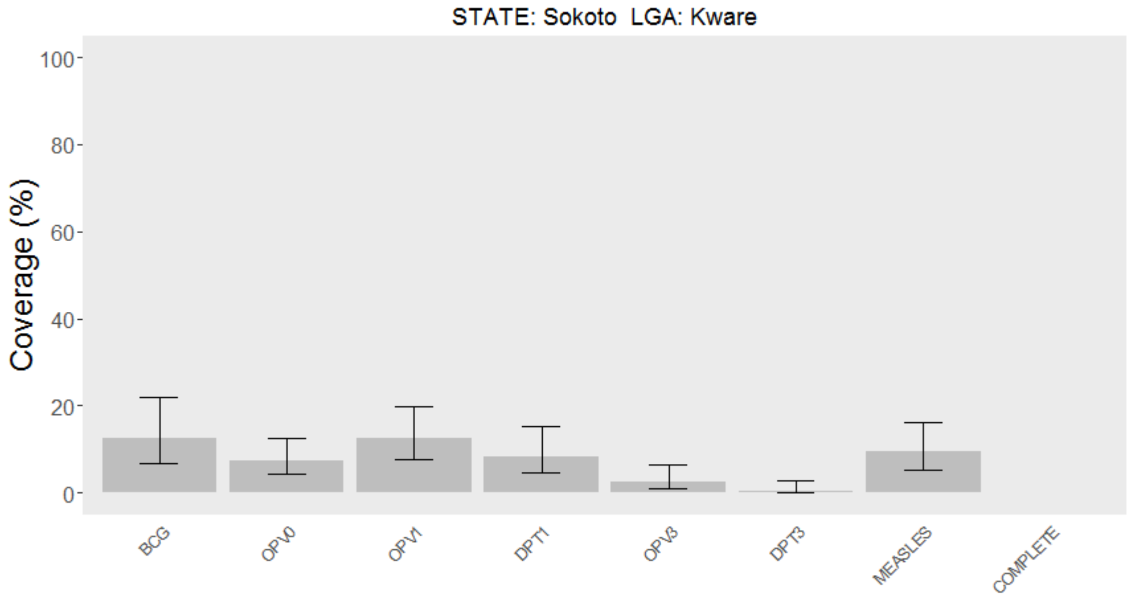
STATE: Sokoto LGA: Kware
Coverage (%)
0
20
40
60
80
100
BCG
OPV0
OPV1
DPT1
OPV3
DPT3
MEASLES
COMPLETE

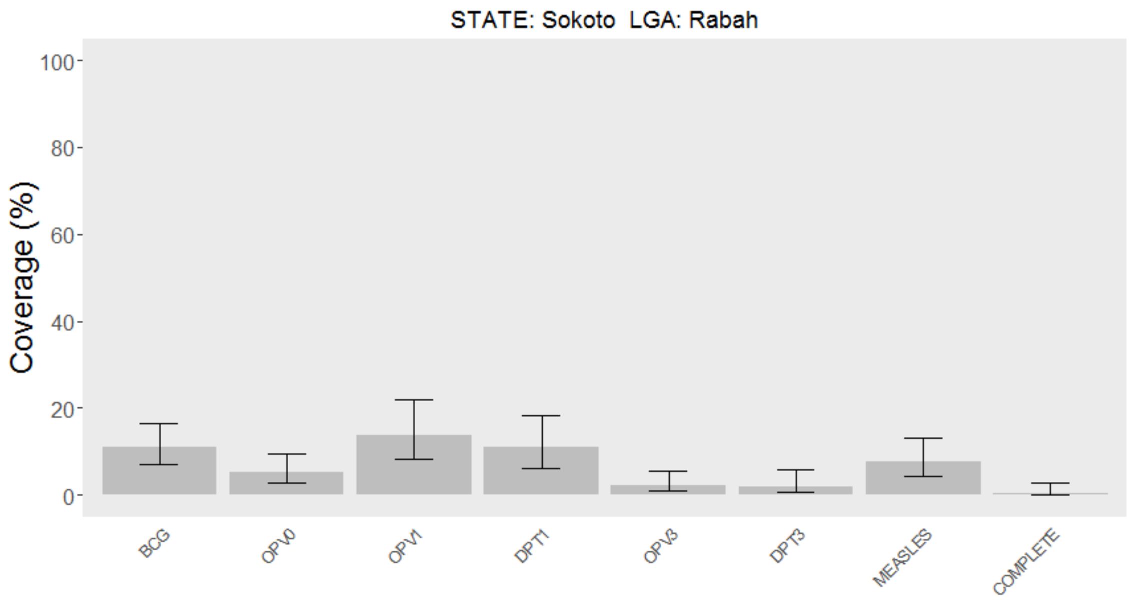

STATE: Sokoto LGA: Rabah
Coverage (%)
100
80
60
40
20
0
BCG
OPV0
OPV1
DPT1
OPV3
DPT3
MEASLES
COMPLETE

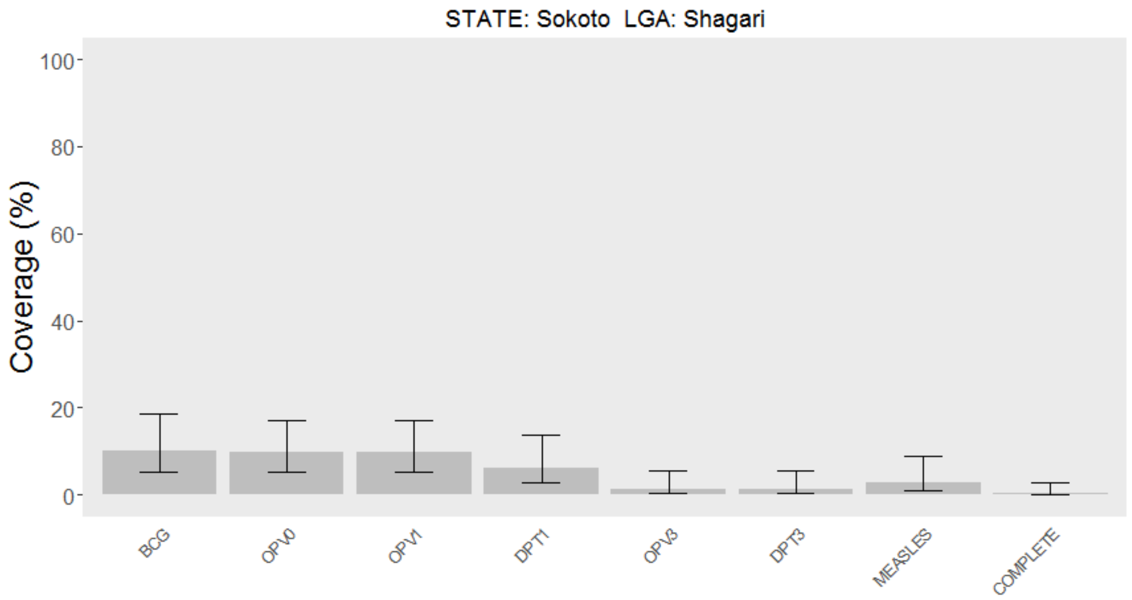

STATE: Sokoto LGA: Shagari
Coverage (%)
100
80
60
40
20
0
BCG
OPV0
OPV1
DPT1
OPV3
DPT3
MEASLES
COMPLETE

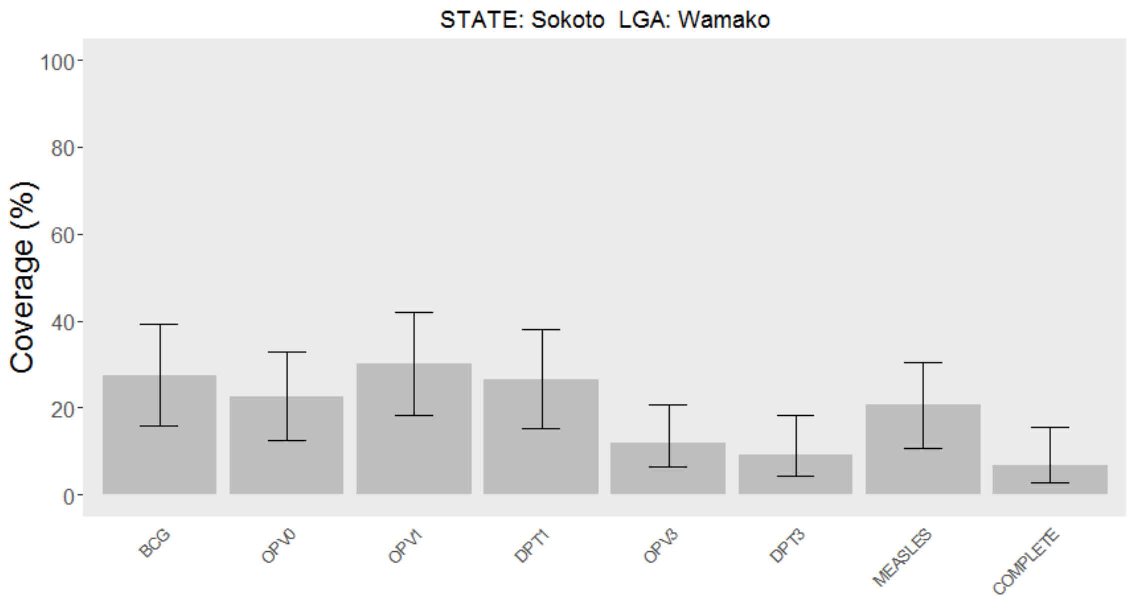
STATE: Sokoto LGA: Wamako
Coverage (%)
0
20
40
60
80
100
BCG
OPV0
OPV1
DPT1
OPV3
DPT3
MEASLES
COMPLETE

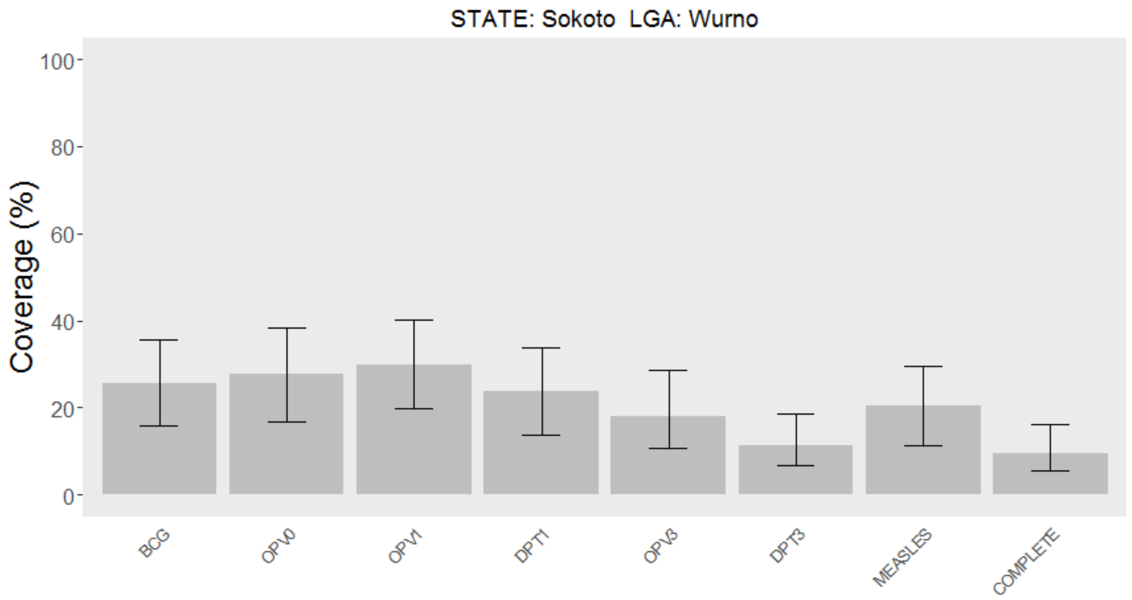
STATE: Sokoto LGA: Wurno
Coverage (%)
0
20
40
60
80
100
BCG
OPV0
OPV1
DPT1
OPV3
DPT3
MEASLES
COMPLETE

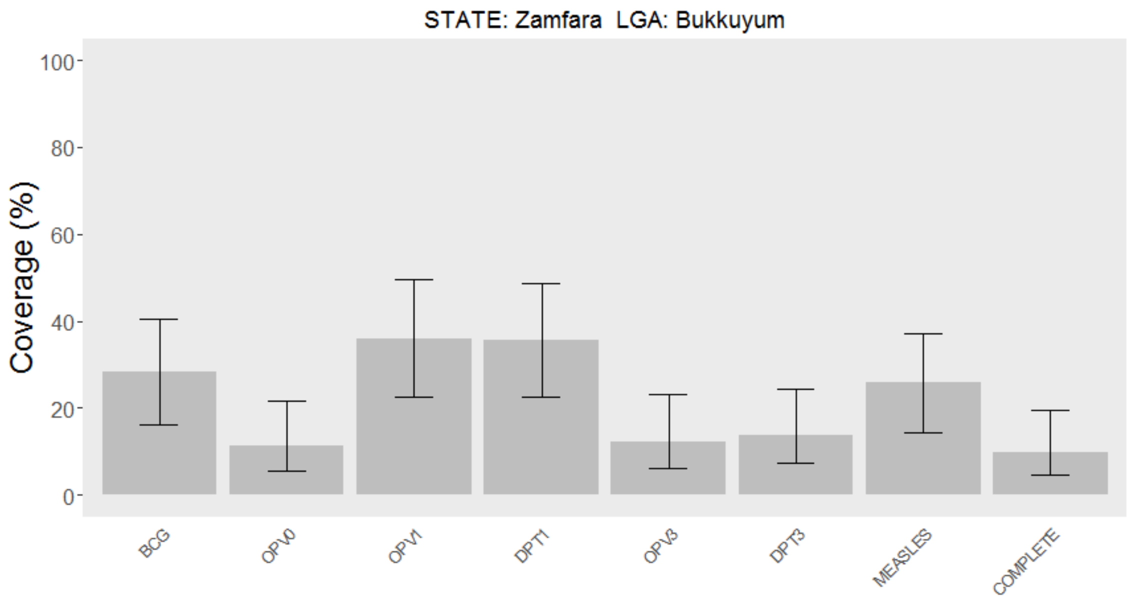
STATE: Zamfara LGA: Bukkuyum
Coverage (%)
100
80
60
40
20
0
BCG
OPV0
OPV1
DPT1
OPV3
DPT3
MEASLES
COMPLETE

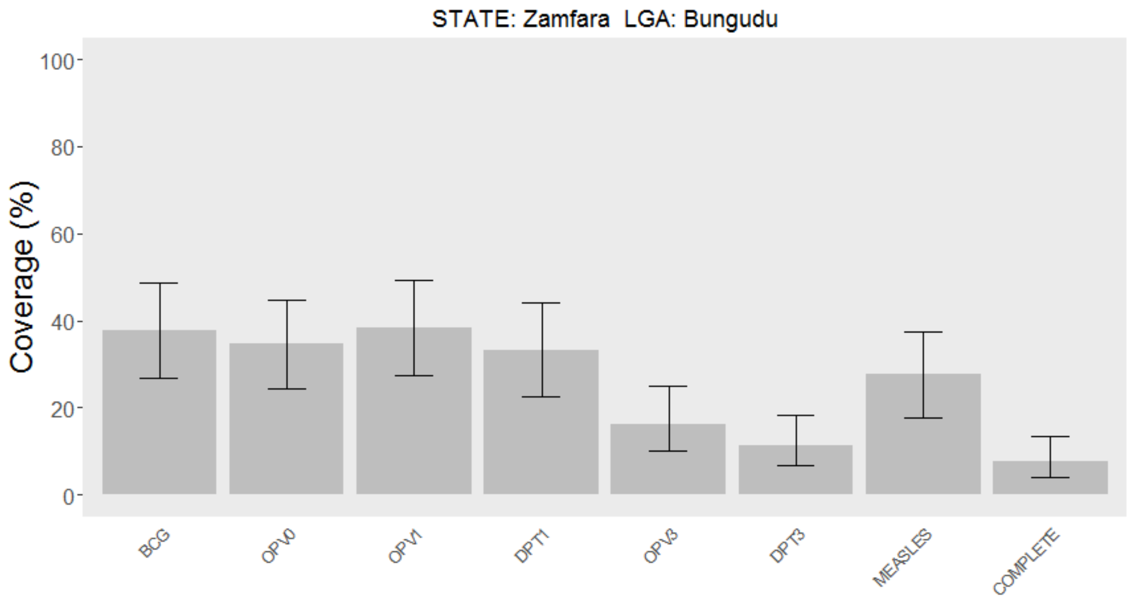
STATE: Zamfara LGA: Bungudu
Coverage (%)
100
80
60
40
20
0
BCG
OPV0
OPV1
DPT1
OPV3
DPT3
MEASLES
COMPLETE

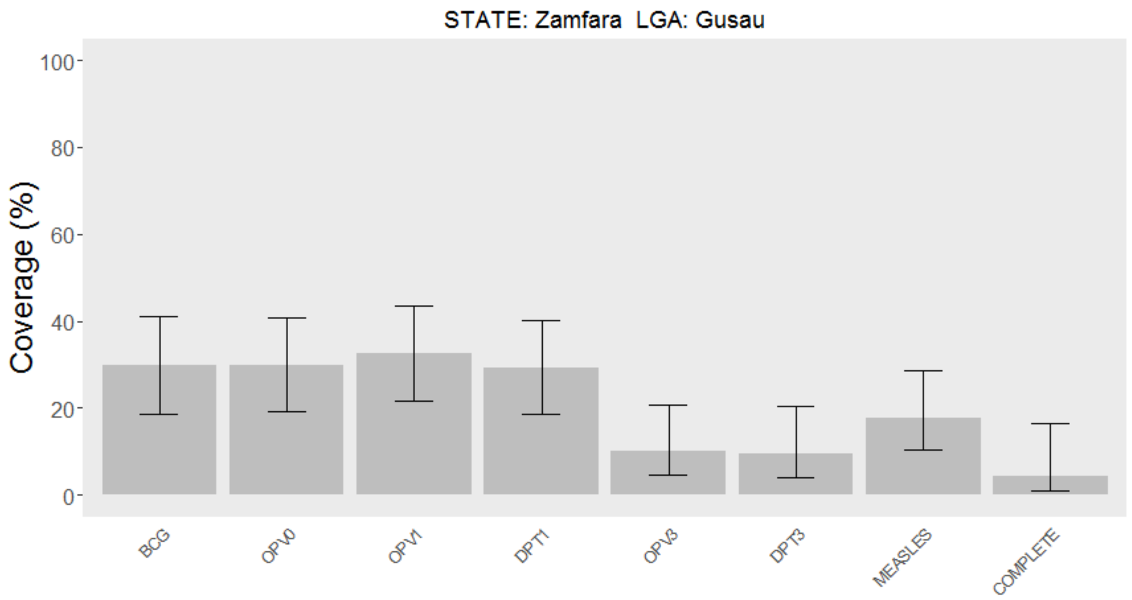
STATE: Zamfara LGA: Gusau
Coverage (%)
0
20
40
60
80
100
BCG
OPV0
OPV1
DPT1
OPV3
DPT3
MEASLES
COMPLETE

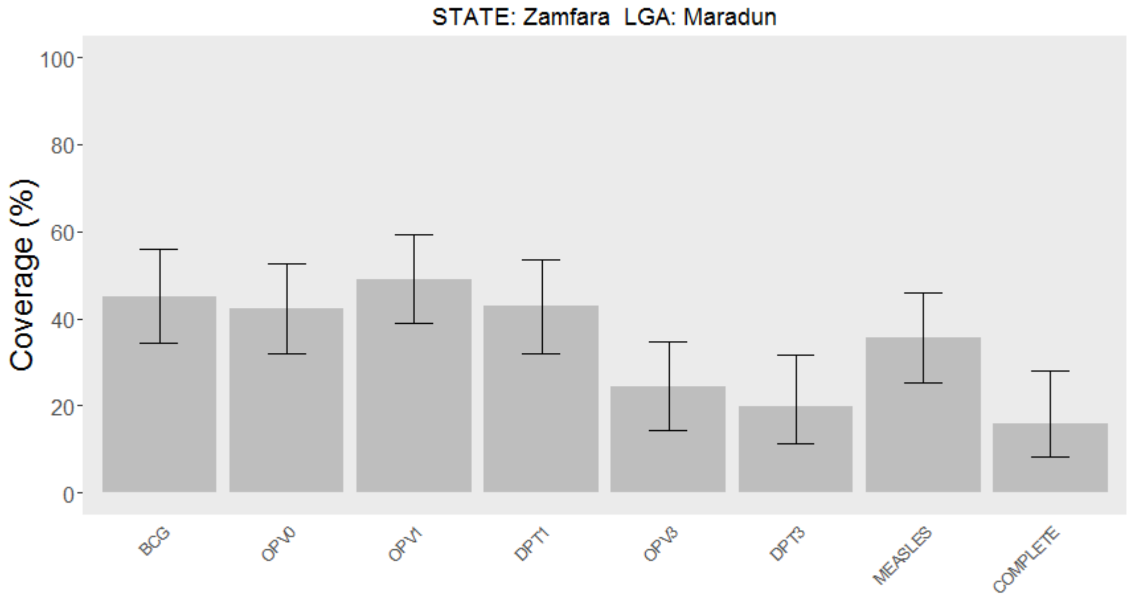
STATE: Zamfara LGA: Maradun
Coverage (%)
0
20
40
60
80
100
BCG
OPV0
OPV1
DPT1
OPV3
DPT3
MEASLES
COMPLETE

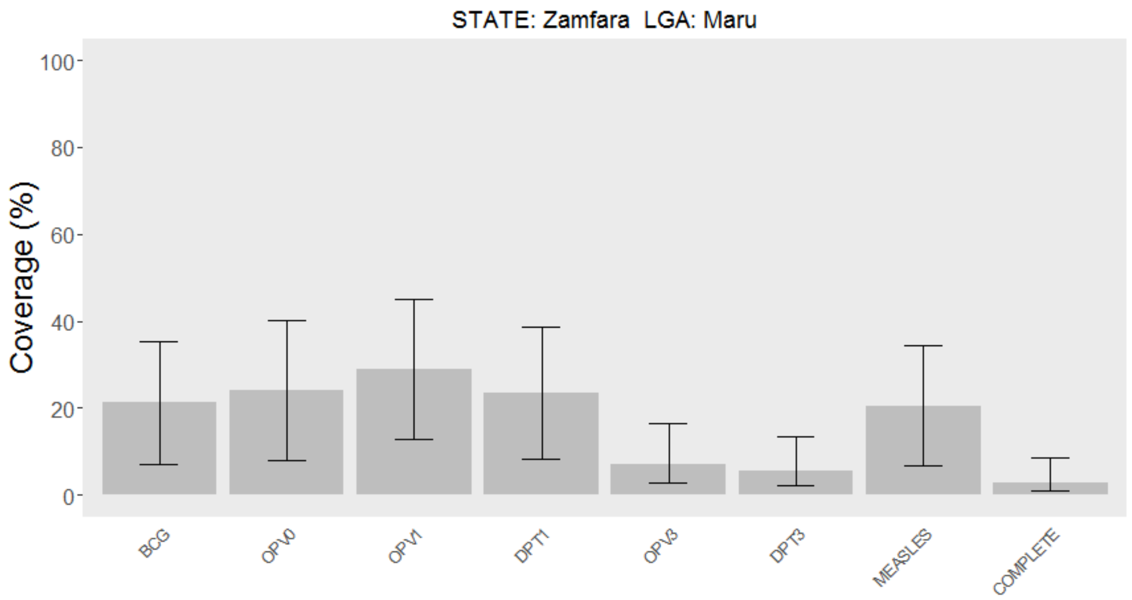
STATE: Zamfara LGA: Maru
Coverage (%)
100
80
60
40
20
0
BCG
OPV0
OPV1
DPT1
OPV3
DPT3
MEASLES
COMPLETE

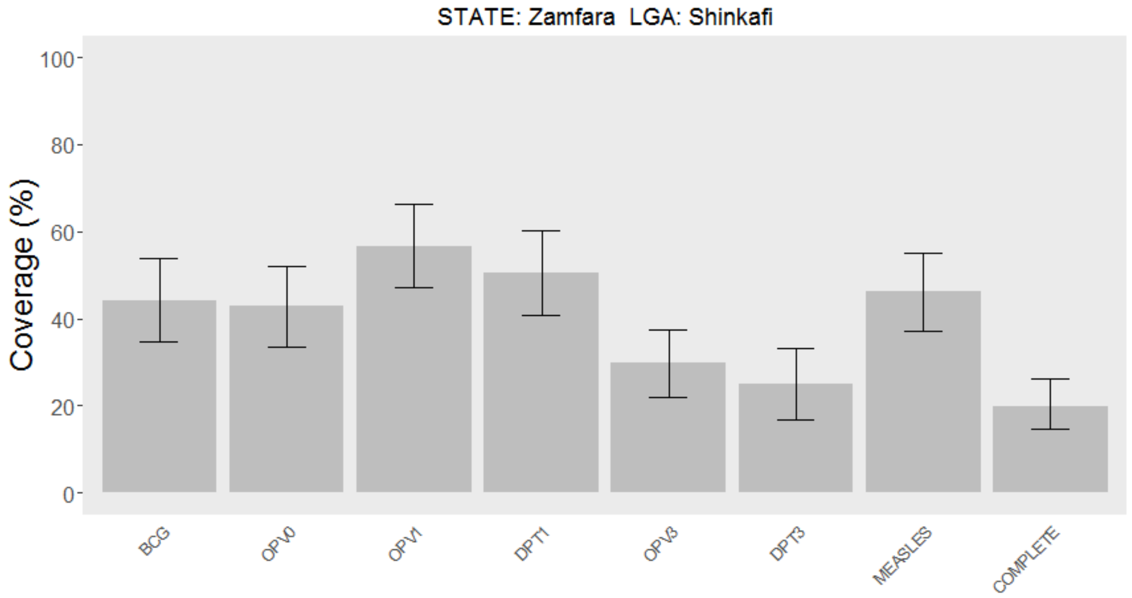
STATE: Zamfara LGA: Shinkafi
Coverage (%)
100
80
60
40
20
0
BCG
OPV0
OPV1
DPT1
OPV3
DPT3
MEASLES
COMPLETE
