# Supplementary material for: Routine Vaccination Coverage in Northern Nigeria: Results from 40 District-Level Cluster Surveys, 2014-2015
Source: PLoS One. 2016 Dec 9;11(12):e0167835. doi: 10.1371/journal.pone.0167835 (PMC5148043; doi:10.1371/journal.pone.0167835)
Supplement: S4 Appendix — This is an example of the slide sets that were distributed to each LGA after data analysis was completed. (PDF) [file pone.0167835.s004.pdf]

Routine Immunization Coverage Survey

Polio High Risk LGAs
Northern Nigeria, 2014-2015

CDC - Nigeria

NSTOP

NFELTP

NPHCDA

Project Goal

As part of efforts to strengthen the routine immunization program, NSTOP planned to conduct coverage surveys in high-risk LGAs during 2014 to evaluate routine immunization coverage in children 12–23 months of age

Objectives

1) To estimate LGA-level RI coverage

2) To estimate coverage in recent polio vaccination campaign rounds

3) To describe reasons for non-vaccination

4) To identify primary sources of vaccination information

5) To train the LGA immunization program team on conducting future household surveys

Intended Use of Data

- Summary reports will be developed for each LGA
- Results will be reported to the state-level and national NPHCDA immunization program staff
- Data will be used to evaluate the vaccination coverage of polio and other antigens in high-risk LGAs
  - Useful for regional planning
  - Documenting Nigeria’s progress towards polio eradication

Survey methodology

- Cluster survey
- Two-stage selection of clusters and households
  - Cluster selection
    - “Enumeration Areas” from 2006 census (NPC)
    - Using PPS
  - Household and child selection
    - Households selected by movement through cluster from a randomly selected start point
    - Collect information on **ONE** child 12–23 months of age within the selected household

Survey methodology

- A total of ~210 children interviewed per LGA
  - 30 clusters (EAs) in each LGA
  - Quota of 7 children in each cluster
  - Sample size
    - 420 → 393 (2 LGAs in pilot phase)
    - 7980 → 7422 (38 LGAs in phase 1 & phase 2)

- Maps and GPS coordinates used
  - For each selected cluster
  - Used for locating starting point and movement plan

Questionnaire

- Standardized paper interview questionnaires
  - Bi-lingual in English and Hausa
  - Read aloud by trained interviewers
- Interview questions include
  - Socio-demographic information
  - Vaccination history from routine immunization
  - Vaccination history from most recent polio IPD round
  - Awareness of opportunities for routine immunizations
  - Reasons for non-vaccination

Project Phases

- Field testing of survey instruments
  - February 2014
  - Administered questionnaires to English and Hausa speaking women around FCT
- Pilot phase
  - May 2014; 2 LGAs in Kaduna state
- Phase I
  - October 2014; 19 LGAs (*due to resource limitations, Ebola*)
- Phase II
  - January 2015; 19 additional LGAs

Study Population

- Target: children 12–23 months of age

- In polio high-risk LGAs with NSLO assigned

  - Pilot phase
    - Kaduna (2)
  - Phase 1
    - Katsina (6)
    - Jigawa (6)
    - Sokoto (6)
    - FCT (1)
  - Phase 2
    - Bauchi (4)
    - Kaduna (6)
    - Kebbi (3)
    - Zamfara (6)

RI Coverage Survey LGAs, Northern Nigeria

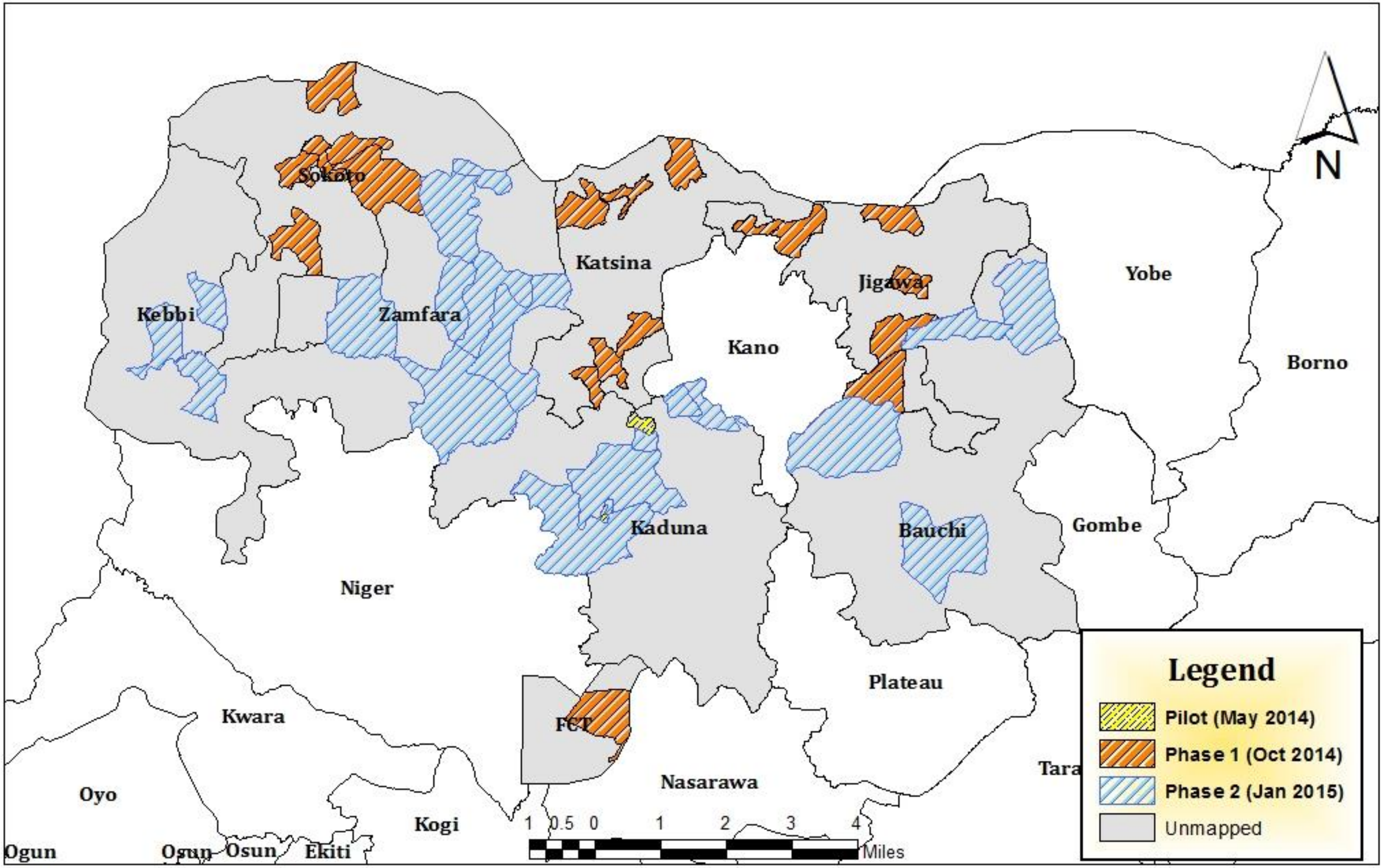

Training and Fieldwork

- Training
  - Central & State level for supervisors
  - LGA level for interviewers
- Fieldwork
  - Team supervision by NFELTP residents
  - Senior supervision by central and state NSTOP officers
    - Conducted spot checks
    - Assisted with finding settlements and cluster start points

Data Entry and Analysis

- All data were entered in an electronic database
  - CSPro software (free software)
  - In Abuja at the NSTOP office
  - Supervised by NSTOP data management team
  - Hired data clerks + NFELTP residents
- Data analyzed using SAS and SPSS

LGA RESULTS – MARU,
ZAMFARA STATE

Results of HH visits in MARU LGA

	N (%)
1+ Eligible child	159 (82.0)
No eligible child	34 (17.5)
No one home	1 (0.5)
Vacant dwelling	0 (0.0)
Missing	0 (0.0)
Refusal	0 (0.0)
**Total**	**194**

Results of the interview with mother or caregiver of eligible infant age 12-23 months

Completed questionnaire	159 (100.0)
Mother/Caregiver absent	0 (0.0)
**Total**	**159**

Socio-demographic factors of survey population

	MARU LGA<br>N (%)
**Sex**	
Male	62 (44.0)
Female	79 (56.0)
**Number of children**	
1	16 (11.3)
2-4	58 (41.1)
5+	58 (41.1)
Missing/Don't Know	9 (6.4)
**Highest level of maternal education**	
None	10 (7.1)
Primary	4 (2.8)
Secondary	3 (2.1)
Post-secondary	1 (0.7)
Quranic schools	122 (86.5)
Missing	1 (0.7)
**Highest level of household head education**	
None	1 (0.7)
Primary	4 (2.8)
Secondary	7 (5.0)
Post-secondary	5 (3.5)
Quranic schools	123 (87.2)
Missing	1 (0.7)
**Rural**	127 (90.1)
**Urban**	14 (9.9)
**Total**	**141**

Access to RI in MARU LGA

	**N (%)**
Ever use of RI*	45 (31.9)

*denominator = all eligible children (141)

Vaccination card findings

	**N (%)**
Ever received a vaccine card†	35 (77.8)
Card retention¶	22 (62.9)
Completed cards§	4 (18.2)

†denominator = ever use of RI (45)
¶denominator = ever received vaccine card (35); required interviewer to see card
§ denominator = card retention (22); complete DD/MM/YY on card for BCG, OPV1/OPV2/OPV3, DPT1/DPT2/DPT3,Measles
**based on revised Nigeria EPI schedule

Reason for not receiving all vaccinations through routine health services in MARU LGA*

	N (%)
Access to RI	30 (25.9)
Knowledge/Education	42 (36.2)
Illness/Side effects	12 (10.3)
Other: Mother too busy, plan to do it later	11 (9.5)
Misc	21 (18.1)
**Total**	**116**

*asked of all participants who responded ‘no’ to any RI and ‘no’ to receiving all vaccinations; questionnaire responses combined into categories

MARU Coverage estimates by antigen*

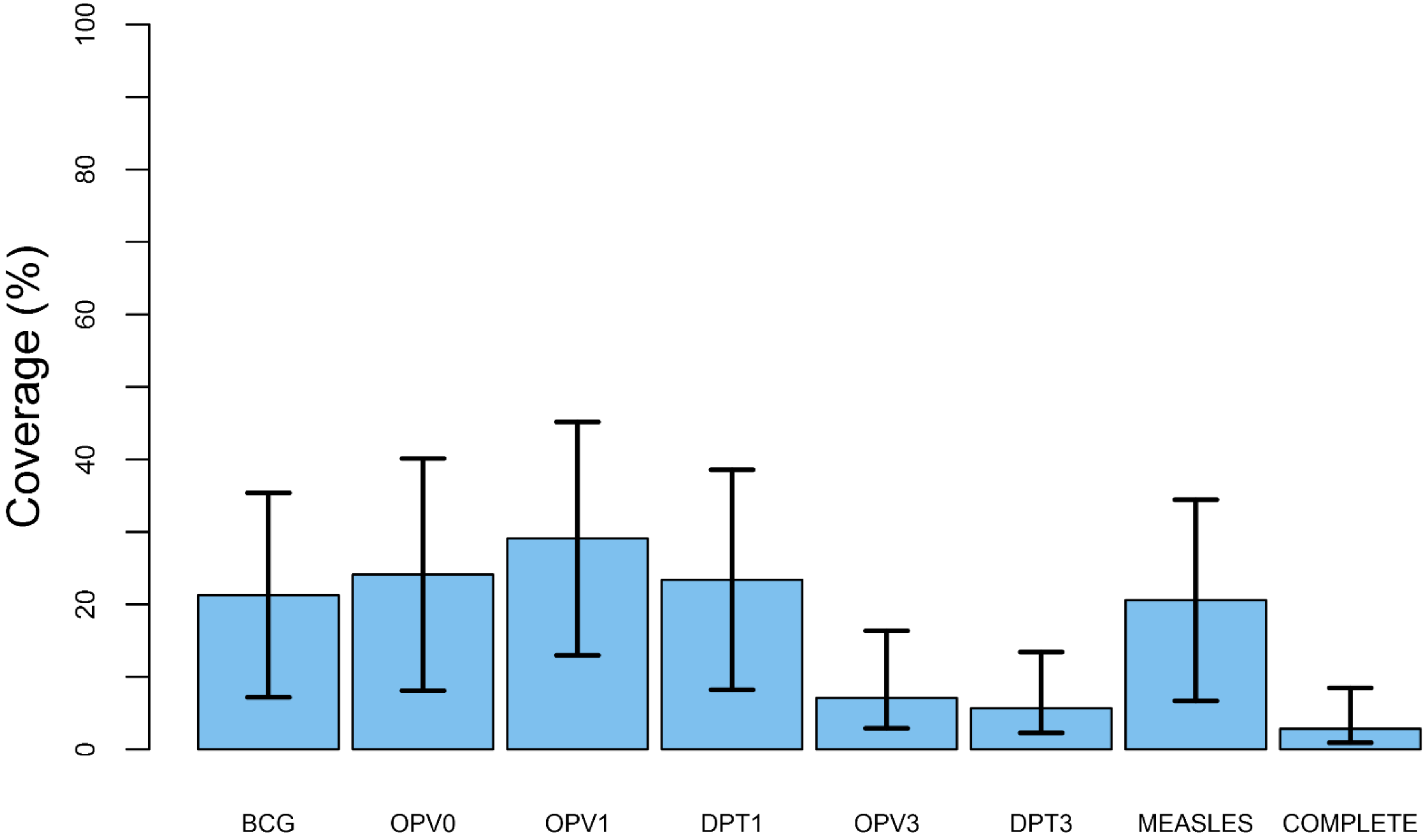

*all coverage estimates combine maternal recall + vaccine card data; complete coverage = 8 antigens (BCG, OPV 1, DPT 1, OPV 2, DPT 2, OPV 3, DPT 3, Measles); does not include OPV doses from SIAs.

Source of information about RI in MARU LGA

	N (%)
Health worker	23 (16.3)
Polio campaign vaccinators	5 (3.5)
Loud speaker/town announcer	59 (41.8)
Poster/banner	0 (0.0)
Radio	31 (22.0)
Television	0 (0.0)
Mobile telephone/SMS	0 (0.0)
Newspapers/magazines	0 (0.0)
Husband/family/neighbor/friends	0 (0.0)
Women's groups	1 (0.7)
Mosque/church	0 (0.0)
Community leader	0 (0.0)
Community mobilizer/VCM	20 (14.2)
Not heard of routine immunization before	1 (0.7)
Don't know	1 (0.7)
Missing	0 (0.0)
**Total**	**141**

Reasons to vaccinate in MARU LGA

	N (%)
**What information helps to decide to vaccinate children through RI services**	
Safety of vaccine	101 (71.6)
Side effects of the vaccine	0 (0.0)
Why vaccinating my child is important	14 (9.9)
What diseases vaccines protect against	4 (2.8)
If sick children can receive the vaccine	1 (0.7)
Time/place for routine vaccination near my home	7 (5.0)
Schedule for vaccination of child	8 (5.7)
Whether my husband/family approves	5 (3.5)
Whether my religious leader approves	0 (0.0)
Whether my community leader approves	0 (0.0)
Other	1 (0.7)
Missing	0 (0.0)
**Total**	**141**

Participation in SIAs in MARU LGA

	N (%)
**Participation in December 2014 SIA**	121 (85.8)
**Reason for not participating**	
House not visited by team	3 (15.0)
Child absent	0 (0.0)
Vaccine safety/fear of adverse event	0 (0.0)
No felt need	0 (0.0)
Religious/cultural reasons	0 (0.0)
Political reasons	1 (5.0)
Too many rounds	0 (0.0)
Other unmet health needs	0 (0.0)
Mother refused, no reason given	0 (0.0)
Husband/head of HH refused	0 (0.0)
Child sick	1 (5.0)
No mother/cargiver consent given	2 (10.0)
Unhappy wth team	0 (0.0)
Postponed until another time	0 (0.0)
Not aware of campaign	1 (5.0)
Campaign vaccine is not as good as vaccine offered through routine health services	0 (0.0)
Don't know	7 (35.0)
Other	0 (0.0)
Missing	5 (25.0)
**Total**	**20**

Participation in SIAs in MARU LGA

	N (%)
**Participation in November 2014 SIA**	120 (85.1)
**Reason for not participating**	
House not visited by team	3 (14.3)
Child absent	1 (4.8)
Vaccine safety/fear of adverse event	1 (4.8)
No felt need	0 (0.0)
Religious/cultural reasons	0 (0.0)
Political reasons	1 (4.8)
Too many rounds	0 (0.0)
Other unmet health needs	0 (0.0)
Mother refused, no reason given	0 (0.0)
Husband/head of HH refused	1 (4.8)
Child sick	0 (0.0)
No mother/cargiver consent given	0 (0.0)
Unhappy wth team	0 (0.0)
Postponed until another time	0 (0.0)
Not aware of campaign	1 (4.8)
Campaign vaccine is not as good as vaccine offered through routine health services	0 (0.0)
Don't know	8 (38.1)
Other	0 (0.0)
Missing	5 (23.8)
**Total**	**21**

Source of information about Polio SIAs in MARU LGA

	N (%)
**Main source of information about polio campaigns**	
Health worker	22 (15.6)
Polio campaign vaccinators	4 (2.8)
Loud speaker/town announcer	59 (41.8)
Poster/banner	0 (0.0)
Radio	30 (21.3)
Television	0 (0.0)
Mobile telephone/SMS	0 (0.0)
Newspapers/magazines	-
Husband/family/neighbor/friends	0 (0.0)
Women's groups	1 (0.7)
Mosque/church	0 (0.0)
Community mobilizer/VCM	23 (16.3)
Not heard of polio campaigns	1 (0.7)
Don't know	1 (0.7)
Other	0 (0.0)
Missing	0 (0.0)
**Total**	**141**

SUMMARY: COMPARISON OF ALL PHASE 1 & PHASE 2 LGAS

BCG Routine Immunization Coverage by LGA

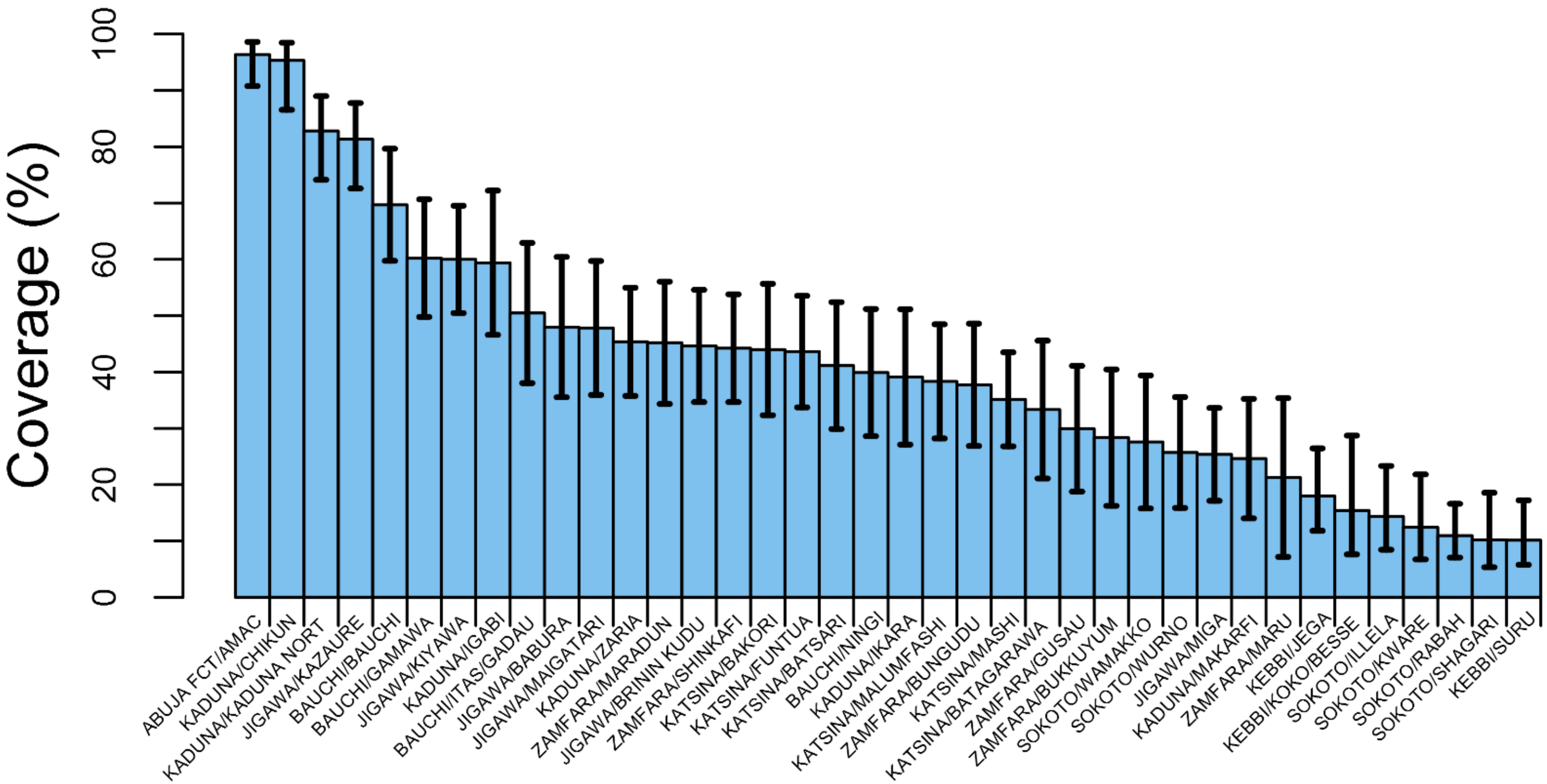

DPT1 Routine Immunization Coverage by LGA

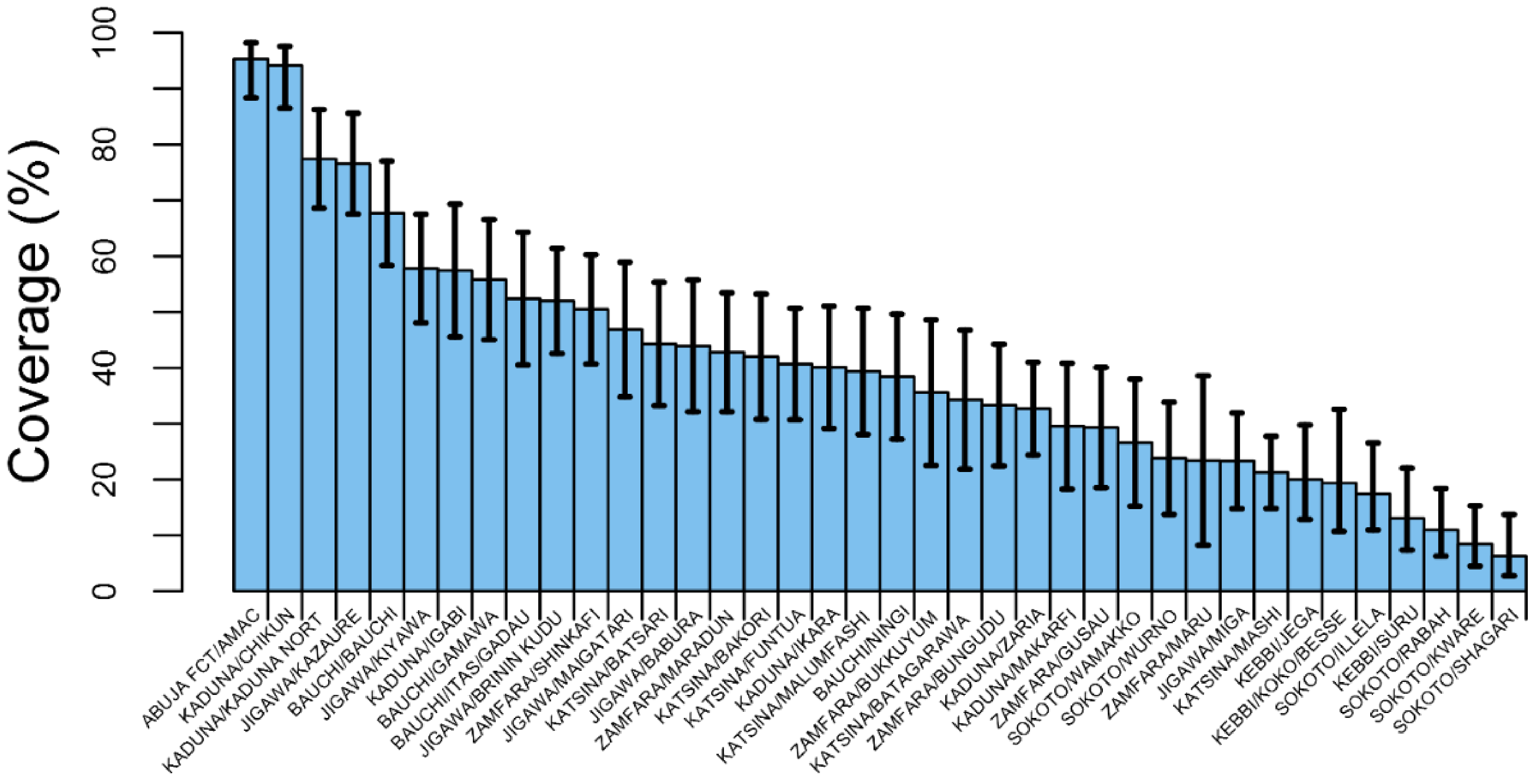

DPT3 Routine Immunization Coverage by LGA

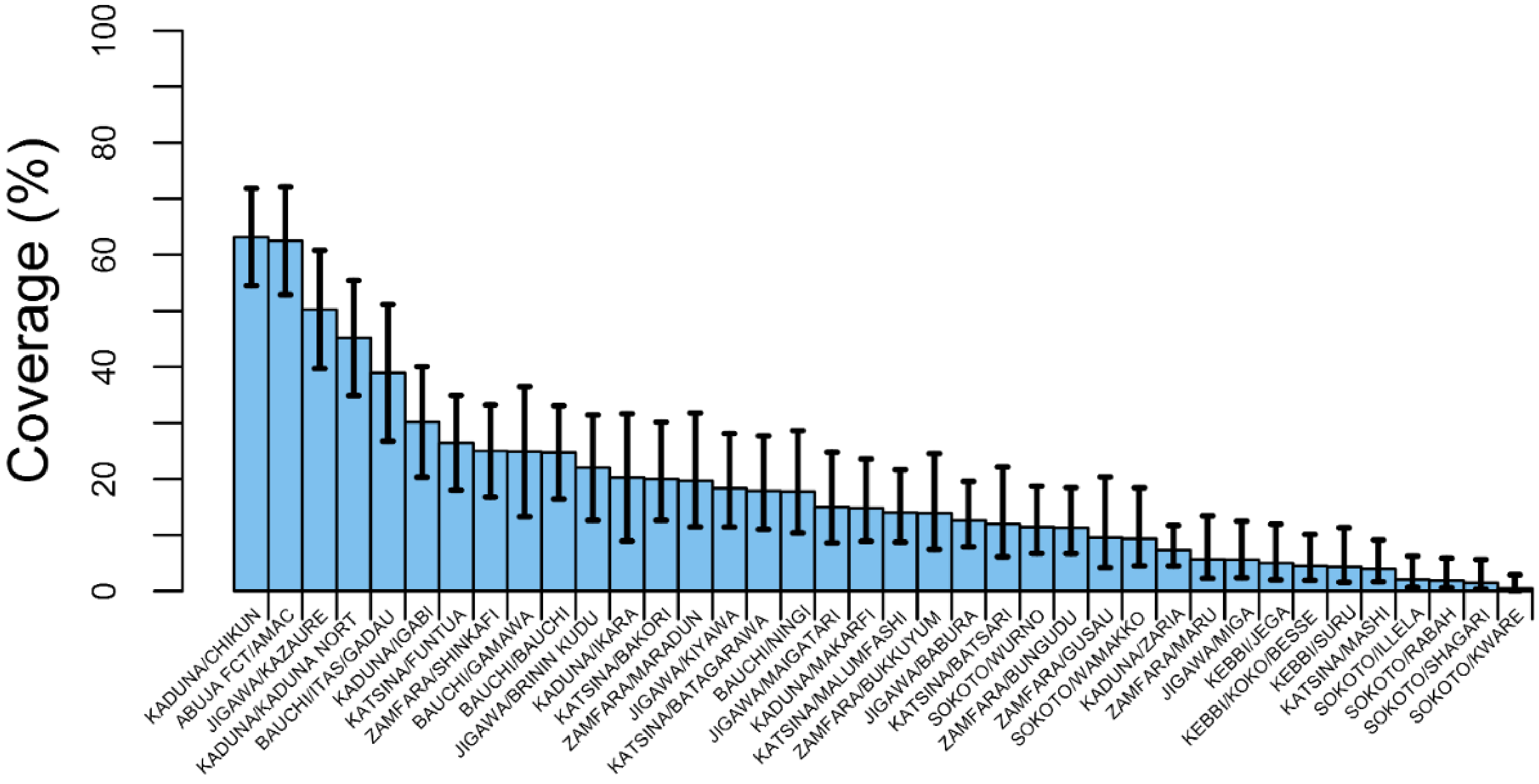

RI DPT3 Vaccination Coverage, Northern Nigeria

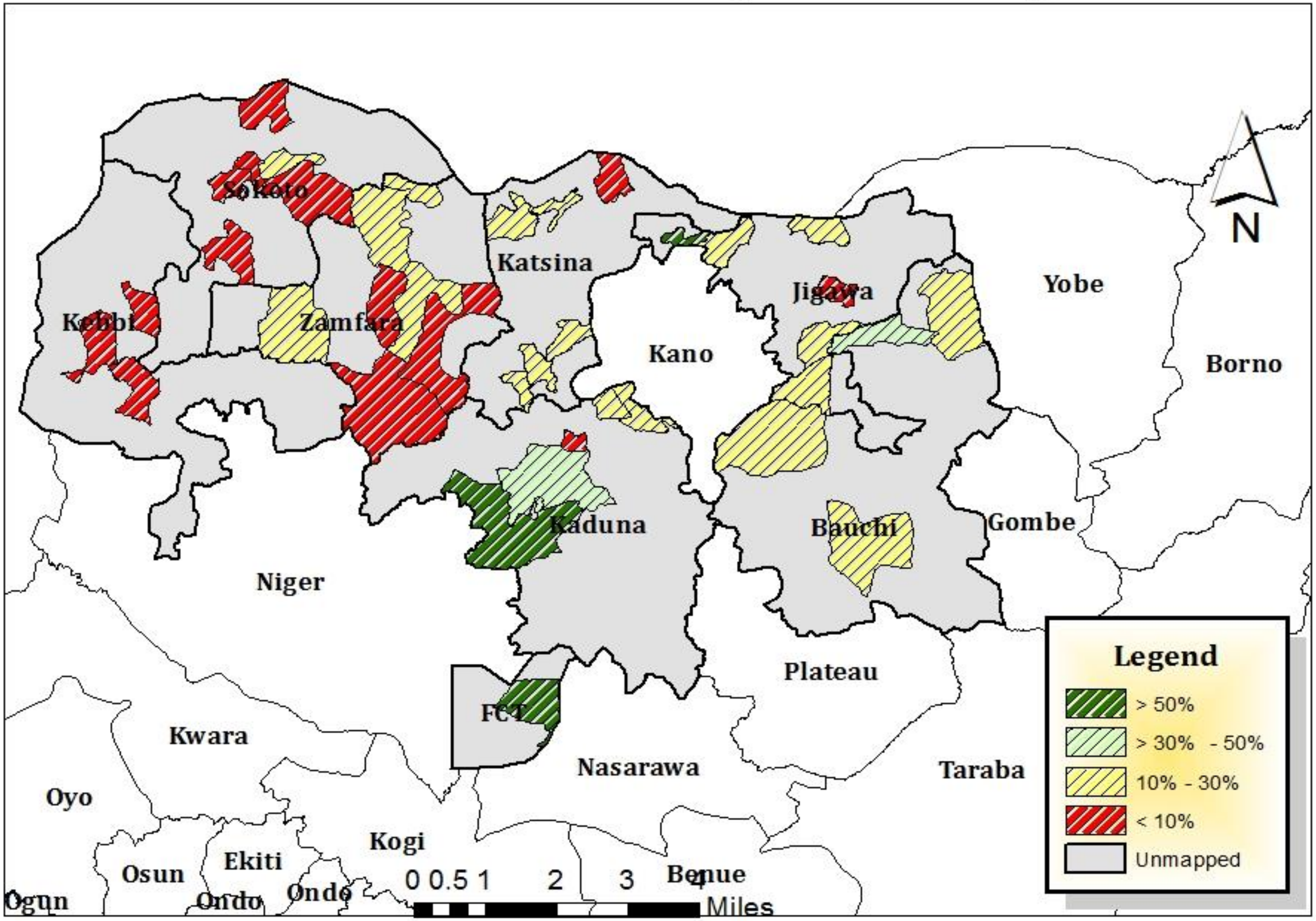

OPV0 Routine Immunization Coverage by LGA

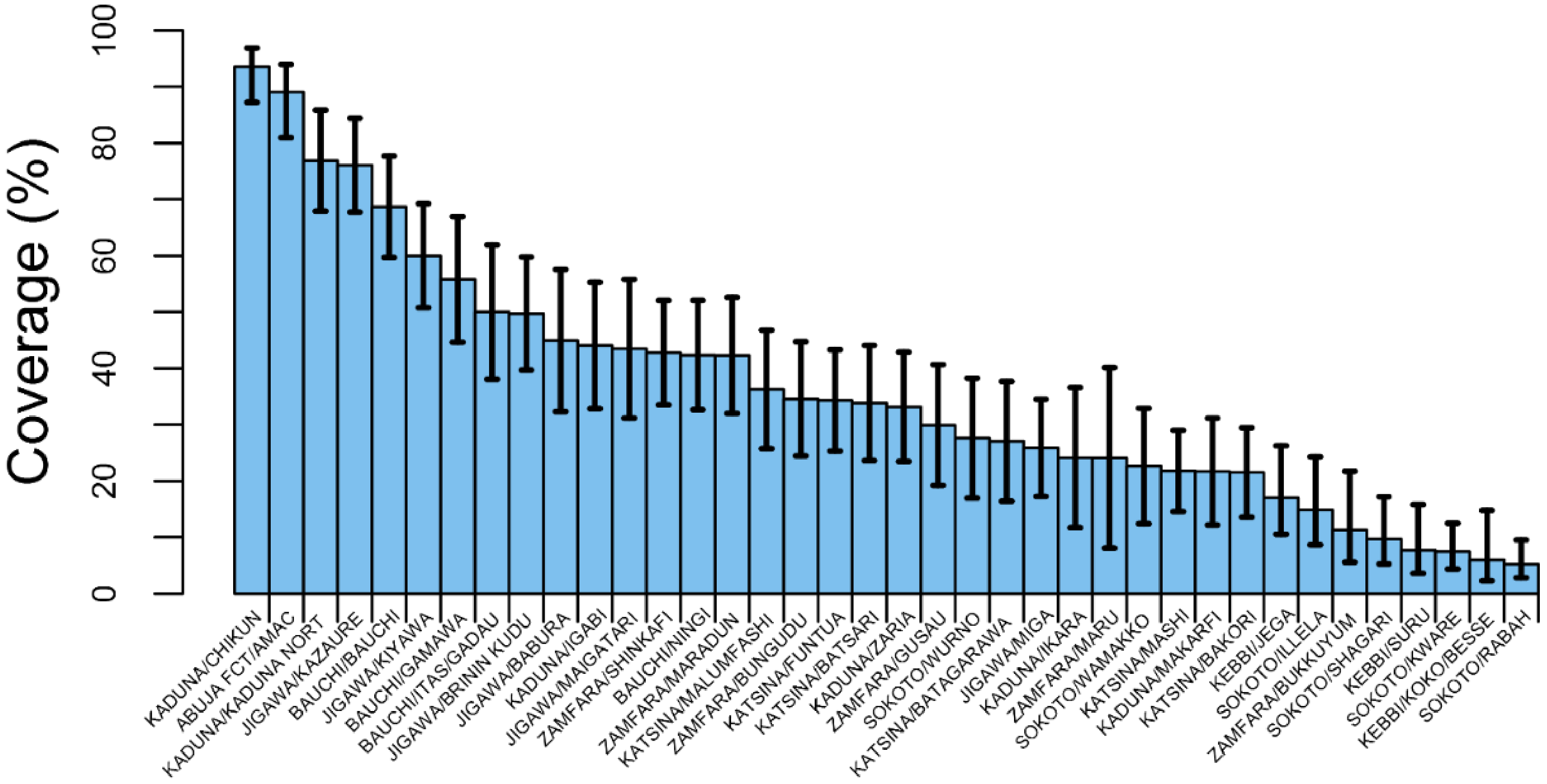

OPV1 Routine Immunization Coverage by LGA

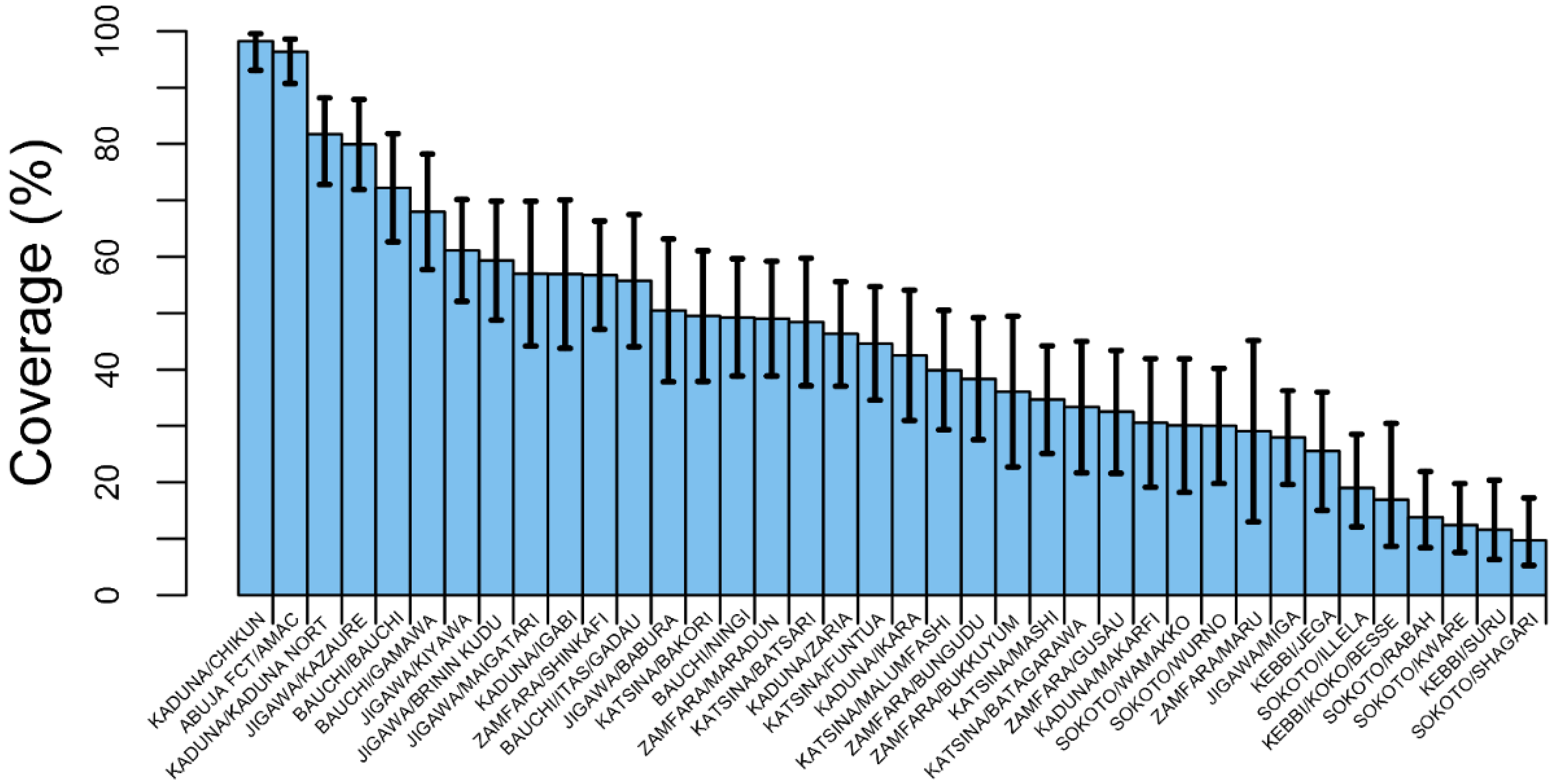

OPV3 Routine Immunization Coverage by LGA

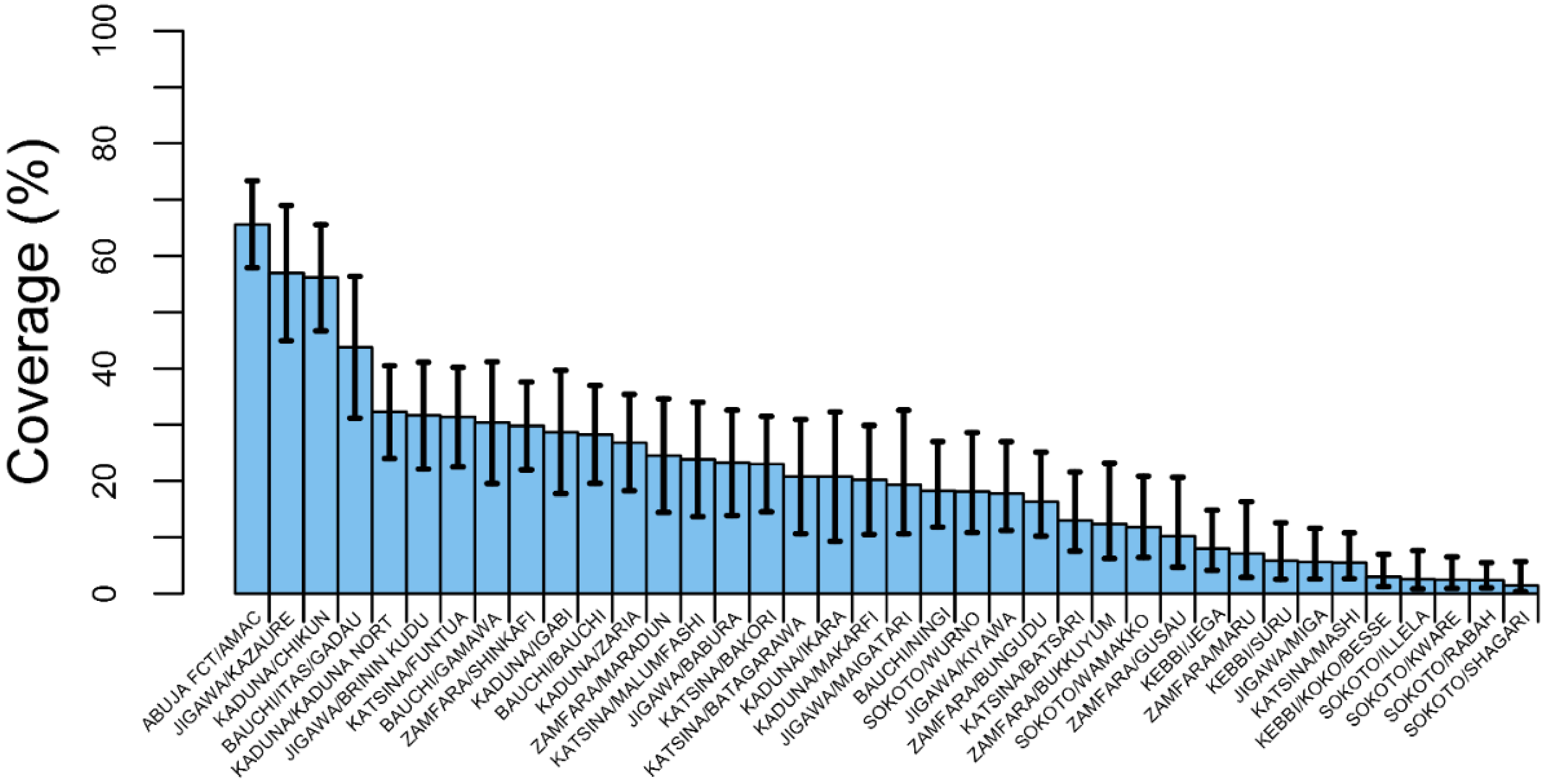

Measles Routine Immunization Coverage by LGA

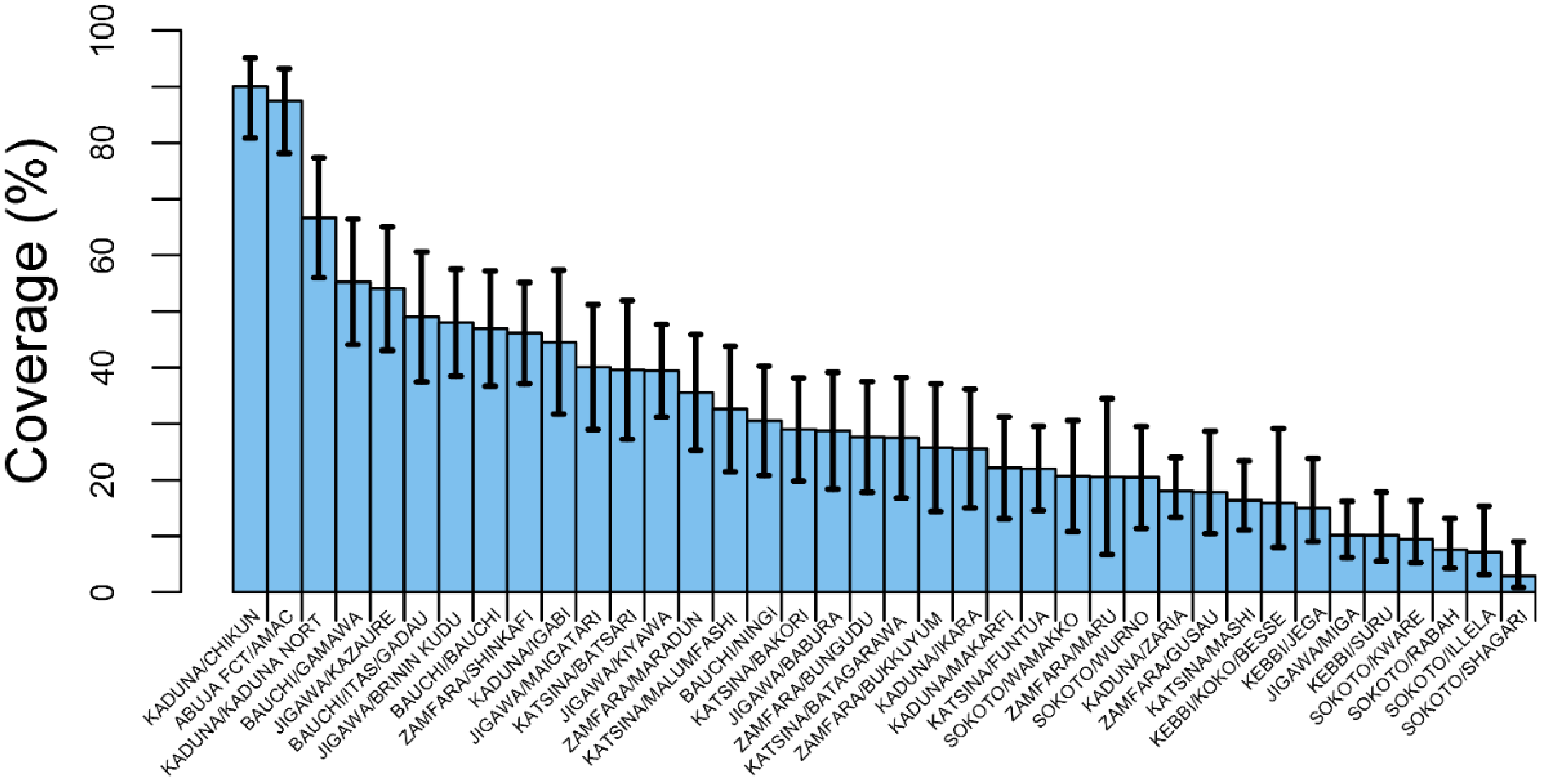

Complete Routine Immunization Coverage by LGA

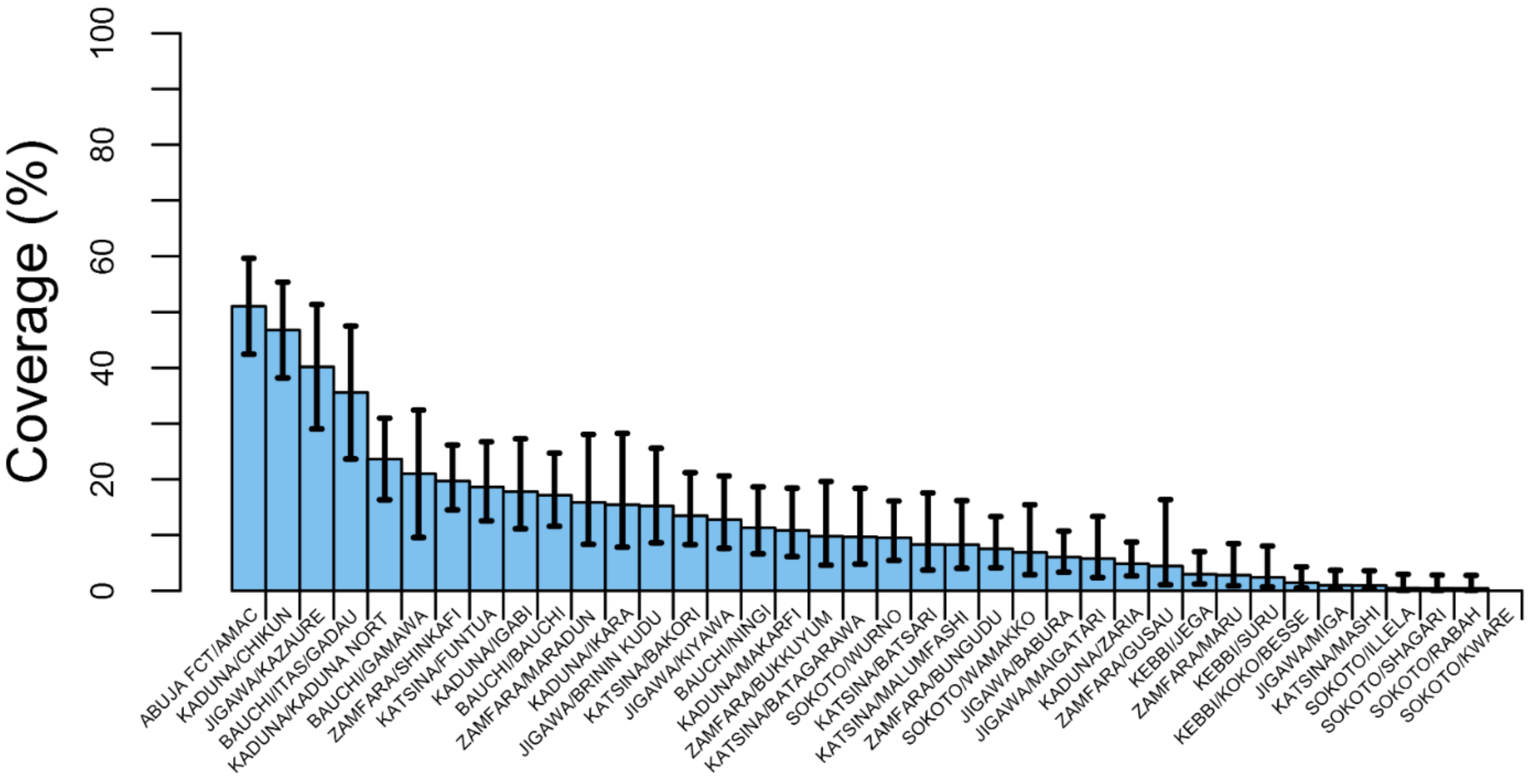

RI Complete Vaccination Coverage, Northern Nigeria

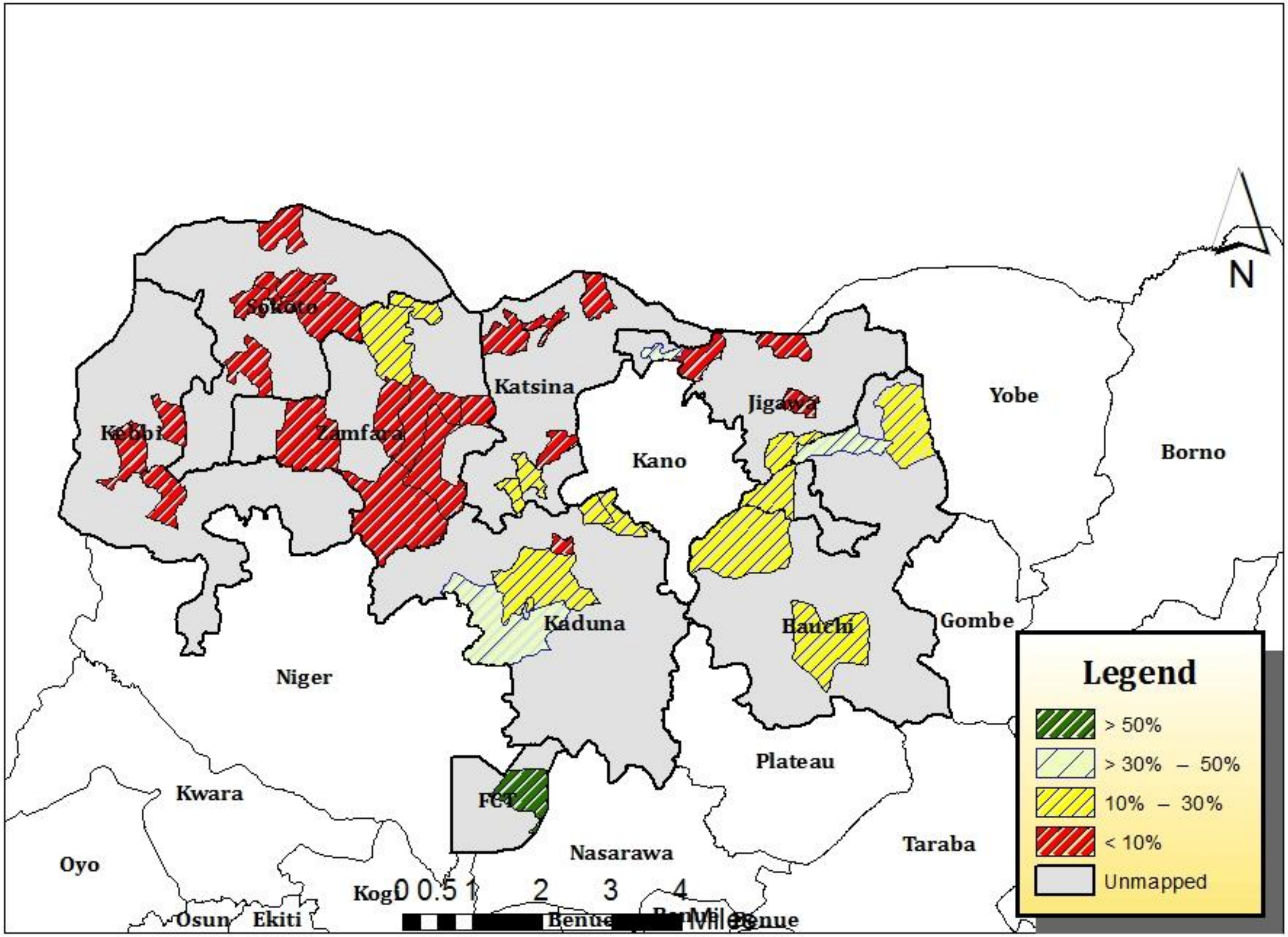

Conclusions – overall survey

- Range of RI coverage exists; low in most LGAs
- Coverage estimates similar to DHS state trends
- Variation seen between LGAs within states
- Reported SIA participation lower than LQAS in certain LGAs
- KAP questions
    - Lack of knowledge/education/access to RI most common reasons for non-vaccination
    - Lack of knowledge more predominant in low coverage rural LGAs
    - Loud speaker/town announcer/radio more common source of information in rural areas; health worker most common source in urban areas
    - Husband/HH acceptance more important in traditional, rural households
- Survey methodology
    - Survey methodology and data quality improved with each phase of survey
    - Inconsistent/missing documentation of dates of vaccines on card

Summary – MARU LGA

- Sociodemographic
  - Geography
    - Mainly rural clusters (90.1%)
  - Education
    - Highest proportion of level of maternal education is Quranic school (86.5%)
    - Highest proportion of level of household head education is Quranic school (87.2%)

Summary – MARU LGA

- Use of RI
  - Reported use of RI services sub-optimal (31.9%)
  - DPT3 coverage low (<10.0%)
  - Lack of knowledge/education most common obstacle to using RI (36.2%)
  - Safety of vaccine reported most important in decision making for vaccination (71.6%)
  - Most common source of RI information is Loud speaker/Town announcer(41.8%)

- SIAs
  - Most common reasons for non participation
    - Don't know (35%)
    - No vaccine team visit (15%)
  - Common sources of SIA information
    - Loud speaker/Town announcer (41.8%)
    - Radio (21.3%)

Survey limitations

- Only high risk polio LGAs surveyed
- ‘Empty’ clusters
  - Nomadic settlements
  - Insecurity
- Unable to analyze sub-populations due to small sample sizes
- Restricted analysis of timeliness of vaccination due to sparse vaccine card data
